# A Comprehensive and Unified Survey on Blockchain-Enabled SDN Cybersecurity: Industry Use Cases, Threat Landscapes, Defense Architectures, and Open Challenges

**DOI:** 10.3390/s26113606

**Published:** 2026-06-05

**Authors:** Deniz Dudukcu, Ali Berkay Gorgulu, Murat Karakus, Rukiye Savran Kiziltepe, Arwa Basbrain

**Affiliations:** 1 Department of Software Engineering, Ankara University, Ankara 06830, Türkiye; ddudukcu@ankara.edu.tr (D.D.); 22290421@ogrenci.ankara.edu.tr (A.B.G.); rukiyekiziltepe@ankara.edu.tr (R.S.K.); 2 Department of Computer Science, King Abdulaziz University, Jeddah 21589, Saudi Arabia; abasabreen@kau.edu.sa

**Keywords:** cybersecurity, attacks, threats, defense, blockchain, software-defined networking, SDN, AI

## Abstract

The convergence of Software-Defined Networking (SDN) and Blockchain (BC) creates a symbiotic relationship in which SDN’s programmable global visibility complements BC’s decentralized, immutable trust model to address critical cybersecurity vulnerabilities and cyber attacks. Addressing the fragmentation in the current literature, this study rigorously investigates BC and SDN (B-SDN) integration with the primary objectives of: (1) differentiating impacts across varied sectors, including the Internet of Things (IoT), Smart Grids, and Vehicular Ad Hoc Networks (VANETs) and more; (2) analyzing critical performance metrics such as energy efficiency and scalability; (3) classifying mitigation, detection, and prevention schemes for specific threats; (4) examining novel Artificial Intelligence (AI) methods; and (5) identifying open challenges and future research directions. Methodologically, this study conducts a survey of state-of-the-art B-SDN studies to investigate six key areas: Industry-specific applications, security mechanisms, defense strategies, defenses against specific attacks, AI integration, and implementation performance. The findings demonstrate that B-SDN integration shows strong potential in simulated and prototype environments to mitigate specific high-impact threats, such as Distributed Denial of Service (DDoS), Man-in-the-Middle (MiTM), and spoofing, across various domains, including IoT, 5G/6G, VANETS, and Smart Grid. Despite the benefits and advantages promised by B-SDN, several limitations continue to exist, including the latency–security trade-off inherent to consensus protocols and scalability constraints in large-scale deployments. Finally, open research challenges persist in AI-driven automation, particularly in Federated Learning (FL) and in the development of standardized interoperability protocols required to enable the transition from conceptual models to operational systems.

## 1. Introduction

Digital infrastructure faces escalating polymorphic cyber-threats that endanger global stability and render traditional centralized security architectures inadequate due to Single Points of Failure (SPoF). Software-Defined Networking (SDN) enables network programmability by decoupling the control and data planes, but introduces centralization vulnerabilities, such as saturation attacks. Blockchain (BC) technology counters this by providing a decentralized, immutable ledger system. The integration of these technologies is driven by rapid market expansion. The BC market is projected to grow by 44.3% annually through 2032, while the SDN market is estimated to reach USD 95 billion with a 17.2% growth rate [1,2]. Major vendors such as Microsoft and Cisco support this robust ecosystem.

The integration of BC and SDN (B-SDN) leverages mutual strengths to resolve inherent weaknesses. BC mitigates SDN vulnerabilities by securing controllers against tampering through decentralized trust models. Simultaneously, SDN optimizes resource management to address scalability limitations inherent to BC. This architecture provides resilient cybersecurity across diverse sectors. In the Internet of Things (IoT) and Industrial IoT (IIoT) domains, B-SDN establishes defense mechanisms for critical infrastructure [3,4]. Vehicular Ad Hoc Networks (VANETs) utilize this integration to prevent node collusion [5,6]. Furthermore, B-SDN secures Smart Grids and next-generation 5G/6G networks [7,8] often enhanced by Artificial Intelligence (AI) for real-time threat detection [9]. Despite the clear benefits, a review of the existing literature reveals specific gaps.

The existing literature often addresses specific B-SDN aspects in isolation. General surveys cover security and non-security domains [10], network protocols [11], feasibility [12], or privacy and AI [9]. IoT-focused studies concentrate on security mechanisms [3,4,13], cloud integration [14], or Network Function Virtualization (NFV) [15,16], while others catalogue attacks [17]. Domain-specific research targets Smart Homes [18], Smart Grids [7], VANETs [5,6], Unmanned Aerial Vehicles (UAVs) [19], and 5G/6G networks [8]. Additionally, threat-centric works analyze Distributed Denial of Service (DDoS) [20,21], or defense taxonomies [22]. However, a critical research gap remains: these studies are often isolated by domain or attack type. There is a lack of a unified, comprehensive survey that integrates diverse industries, specific attack vectors, defense strategies, and performance metrics into a single study.

The primary objective of this comprehensive survey is to delineate the B-SDN landscape by providing the following principal contributions:

1. Analysis of critical performance metrics for defense solutions, focusing on energy efficiency and scalability.
2. Classification of mitigation, detection, and prevention schemes tailored for specific cyber threats within the B-SDN architecture.
3. Examination of novel AI methods and their efficacy within the B-SDN paradigm.
4. Identification of open challenges and future research directions to guide subsequent studies.

**Figure 1:**
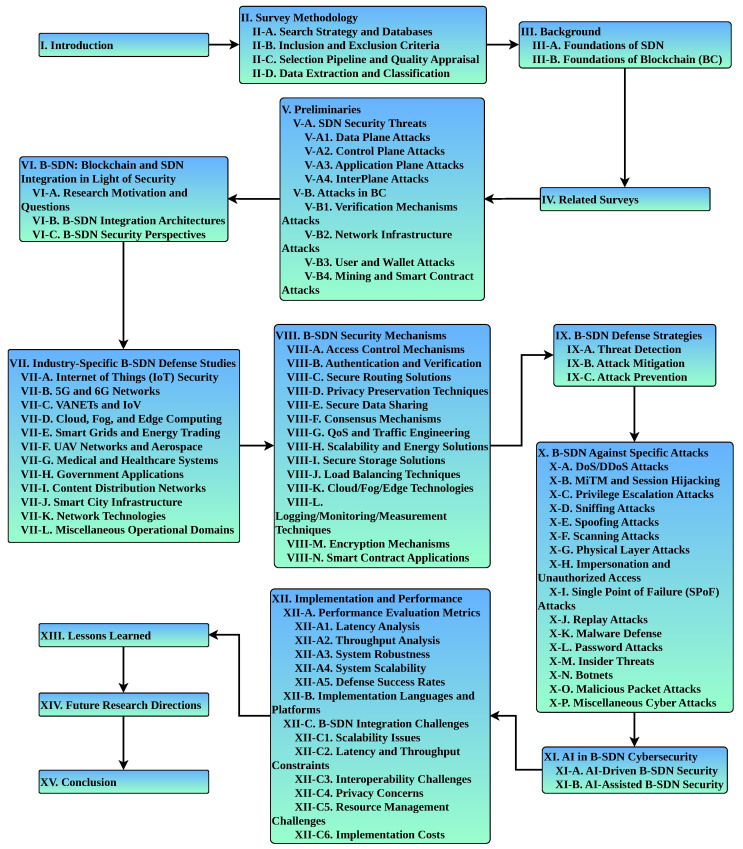
Overall structure of the survey paper and its main sections.

Figure 1 illustrates the survey structure. The remainder of this paper is organized as follows: Section 2 details the systematic review methodology and the study selection pipeline. Section 3 presents the theoretical foundations of SDN and BC, followed by an analysis of existing related surveys in Section 4. Section 5 outlines the threat landscapes of both technologies. Section 6 examines integration motivations and architectures. Section 7 investigates industry-specific defense implementations, whereas Section 8 explores security mechanisms and protocols. Subsequently, Section 9 categorizes detection, mitigation, and prevention strategies, and Section 10 details countermeasures against specific attacks. Section 11 discusses AI integration in security, and Section 12 evaluates performance metrics and integration challenges. Finally, Section 13 presents a synthesis of lessons learned, Section 14 identifies future research opportunities and trends, and Section 15 provides concluding remarks.

## 2. Survey Methodology

To ensure the reproducibility, transparency, and accuracy of this comprehensive survey, a systematic review methodology was adopted. The study selection process followed the Preferred Reporting Items for Systematic Reviews and Meta-Analyses (PRISMA) guidelines [23], providing a structured approach to identifying, screening, and including relevant literature.

### 2.1. Search Strategy and Databases

A comprehensive literature search was conducted across multiple academic databases to capture the intersection of Blockchain, SDN, and cybersecurity. The primary databases searched included IEEE Xplore, ACM Digital Library, SpringerLink, ScienceDirect, Scopus, and Web of Science (WoS). The search was bounded by a time window from January 2016 to early 2026, reflecting the period when B-SDN architectures gained academic and industrial traction. The active database search and literature retrieval phase was conducted between November 2025 and February 2026. To ensure consistency, a unified search string was constructed using Boolean operators and applied uniformly across all selected databases, targeting the title, abstract, keywords, and full manuscript fields: *(“Blockchain” OR “Distributed Ledger” OR “Smart Contract”) AND (“Software-Defined Networking” OR “SDN” OR “Programmable Network”) AND (“Security” OR “Cybersecurity” OR “Threat” OR “Attack” OR “Defense” OR “Mitigation”)*.

### 2.2. Inclusion and Exclusion Criteria

To maintain a sharp analytical focus, explicit inclusion and exclusion criteria were established.

- **Inclusion Criteria:** (1) Peer-reviewed journal articles, conference proceedings, and verified preprints directly proposing or evaluating cybersecurity solutions using integrated B-SDN architectures; (2) Studies focusing on industry-specific applications, security mechanisms, or defense strategies against specific cyber attacks in a B-SDN context; (3) Articles published in the English language.
- **Exclusion Criteria:** (1) Studies examining Blockchain or SDN in isolation without integrating both technologies; (2) Papers focusing purely on B-SDN performance or routing optimization without a clear cybersecurity or threat-mitigation context; (3) Non-peer-reviewed whitepapers, theses, or extended abstracts lacking sufficient technical depth.

### 2.3. Selection Pipeline and Quality Appraisal (PRISMA Workflow)

**Figure 2:**
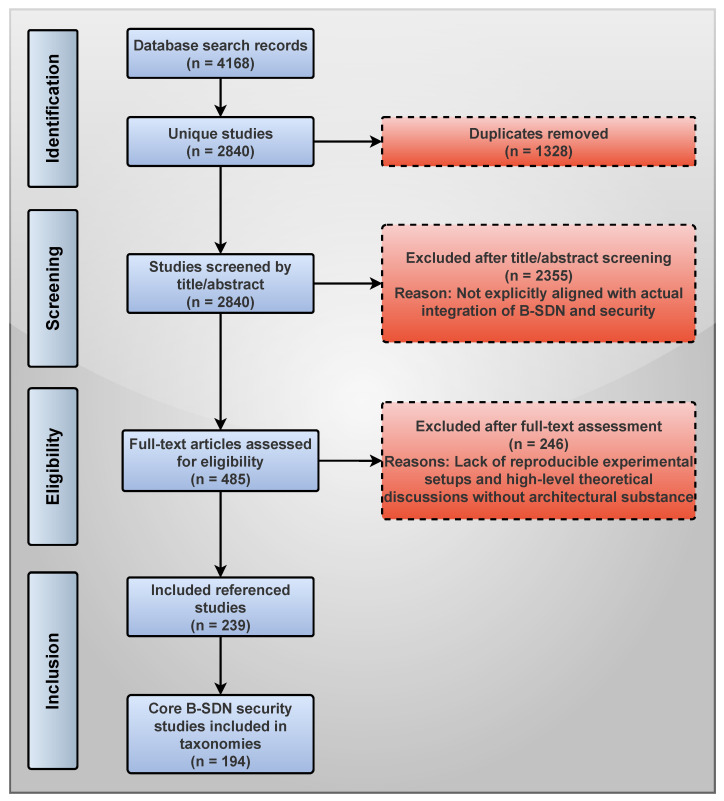
PRISMA flow diagram summarizing the systematic study selection pipeline and exclusion criteria.

The selection pipeline, as illustrated in Figure 2, consisted of three rigorous stages: identification, screening, and inclusion. Initially, the database search yielded an aggregated total of 4168 potential records across all sources. During the initial screening phase, extensive overlapping duplicate records (identified by matching titles, abstracts, and author lists) were removed, leaving 2840 unique studies. A preliminary review of titles and abstracts excluded 2355 papers that, despite triggering keyword matches, did not explicitly align with the actual integration of B-SDN and security. The remaining 485 articles underwent a full-text eligibility assessment. During this phase, quality appraisal and bias-control were enforced by two independent reviewers cross-verifying the technical depth, empirical validation, and relevance of each paper. This appraisal process was guided by the authors’ comprehensive literature knowledge and operationalized through regular, structured evaluation meetings. Specifically, “technical depth” was assessed by requiring a concrete B-SDN architectural design rather than abstract theoretical discussions; “empirical validation” was verified by checking for reproducible experimental setups, defined simulation parameters, or clear prototype metrics; and “relevance” was evaluated based on the study’s explicit focus on threat mitigation rather than general network performance. In cases of disagreement regarding a study’s eligibility, a third independent reviewer mediated the discussion during these regular meetings to reach a final consensus. Articles lacking reproducible experimental setups, those focusing on SDN or Blockchain in isolation, or those only offering high-level theoretical discussions without architectural substance were excluded. This rigorous screening process resulted in a final count of 239 referenced studies, of which 194 were core B-SDN security studies included in the detailed taxonomies and comparative analyses of this survey.

### 2.4. Data Extraction and Classification

Data extracted from the included studies was systematically coded into a multi-dimensional taxonomy. The extraction protocol categorized each paper based on: (1) the target industry or application domain (e.g., IoT, 5G/6G, IoV); (2) the specific security mechanism proposed (e.g., access control, authentication, routing); (3) the overarching defense strategy (detection, mitigation, prevention); and (4) the specific cyber attacks mitigated (e.g., DDoS, MiTM, Spoofing).

Table 1 provides an analytical macro-perspective of the 194 core studies, revealing that current B-SDN research trends are driven by the inherent constraints and vulnerabilities of the underlying technologies. IoT (93 studies) and edge computing (70) take the lead in the application domains, as their highly distributed environments critically require both SDN’s dynamic traffic management and Blockchain’s decentralized trust. Architecturally, Ethereum (65) and Hyperledger (36) are the preferred platforms—leveraging PoW (32) and PBFT (24) consensus models—due to Ethereum’s smart contract ecosystem and Hyperledger’s permissioned, high-throughput capabilities. For empirical validation, Mininet (72) coupled with OpenDaylight (24) or Ryu (19) is the standard choice; this is primarily because Mininet offers lightweight, OS-level virtualization with native OpenFlow (v1.3+) support, while ODL and Ryu provide enterprise modularity and Python-based prototyping flexibility, respectively. The overwhelming focus on mitigating DDoS (99) and spoofing (61) reflect the centralized SDN controller’s susceptibility to resource exhaustion and flow-rule poisoning, prompting a strong emphasis on proactive prevention strategies (123). Finally, the dual prioritization of throughput (107) and detection accuracy (85) as evaluation metrics highlights the primary dilemma in B-SDN design: balancing the cryptographic overhead of Blockchain integration with the stringent performance requirements of real-time network defense.

Beyond the general distribution of topics and tools, it is crucial to understand the empirical maturity of the included literature. To provide this perspective, Table 2 categorizes a representative selection of 34 core studies into four conceptual levels: conceptual frameworks, simulation-based studies, prototype implementations, and real-world deployments. As demonstrated, the majority of the current literature relies heavily on simulation tools or limited prototype setups utilizing virtual machines and constrained edge hardware. While these studies provide robust, well-supported conclusions regarding scalability and mitigation success in controlled environments, conclusions drawn from real-world, large-scale deployments—such as those utilizing commercial cloud labs or active production networks—remain scarce. This distinction explicitly delineates which conclusions in the subsequent sections are backed by physical, operational implementations versus those that remain forward-looking.

## 3. Background

This section delineates the theoretical foundations of SDN and BC, identifying the intrinsic strengths and limitations that drive their convergence. Understanding these mechanisms is a prerequisite for analyzing the economic and technical significance of integrating these technologies.

### 3.1. Foundations of Software-Defined Networking (SDN)

**Figure 3:**
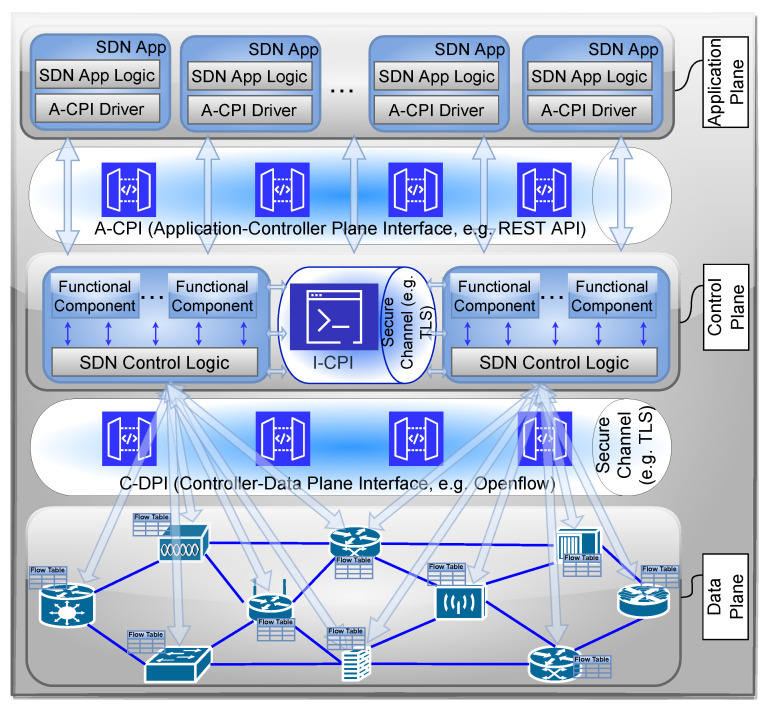
Baseline SDN architecture illustrating the application, control, and data planes interconnected via standardized interfaces (A-CPI, I-CPI, and C-DPI), forming the architectural foundation upon which B-SDN and AI-enabled security mechanisms can be integrated.

Conventional IP-based architectures face operational limitations as cloud computing and data-intensive applications expand, often resulting in complex configurations and vendor lock-in [58]. SDN addresses these constraints by decoupling control and data planes to enable programmability and dynamic resource allocation [59,60,61,62]. This centralized, vendor-agnostic architecture allows operators to optimize capacity and reduce administrative overhead by enabling autonomous software management [63]. The architecture shown in Figure 3 represents the baseline SDN design that underpins B-SDN and AI-enhanced security extensions discussed in this study.

Communication between SDN layers relies on standardized interfaces to ensure interoperability. East–West APIs facilitate information exchange between controllers [64], while Northbound APIs connect applications to the control layer for management functions. Southbound APIs, prominently the OpenFlow protocol, establish connectivity between controllers and data plane devices, serving as the industry standard for this interaction [65,66,67,68,69,70,71,72].

***Data Plane:*** The data layer comprises networking equipment such as routers and switches, where packet forwarding constitutes the fundamental operation. SDN controllers administer these devices via Controller-Data Plane Interfaces (C-DPIs), such as OpenFlow, which transmit messages over secure channels, such as TLS, to ensure connectivity and management integrity.
***Control Plane:*** The control layer consists of software-based controllers that execute forwarding logic via C-DPIs. These controllers utilize Intermediate–Controller Plane Interfaces (I-CPIs) to share state data among themselves [64] and Application–Controller Plane Interfaces (A-CPIs) to interact with network applications for security and management tasks.
***Application Plane:*** The application layer encompasses programs that interface with controllers to execute specific networking tasks using an abstract view of the network topology. These applications connect via open A-CPIs, such as REST APIs, to implement high-level policies and decision-making algorithms.

An SDN application is composed of an SDN App Logic and an A-CPI Driver. They are typically grouped based on the network services they provide, such as security, traffic engineering, and load balancing.

**Figure 4:**
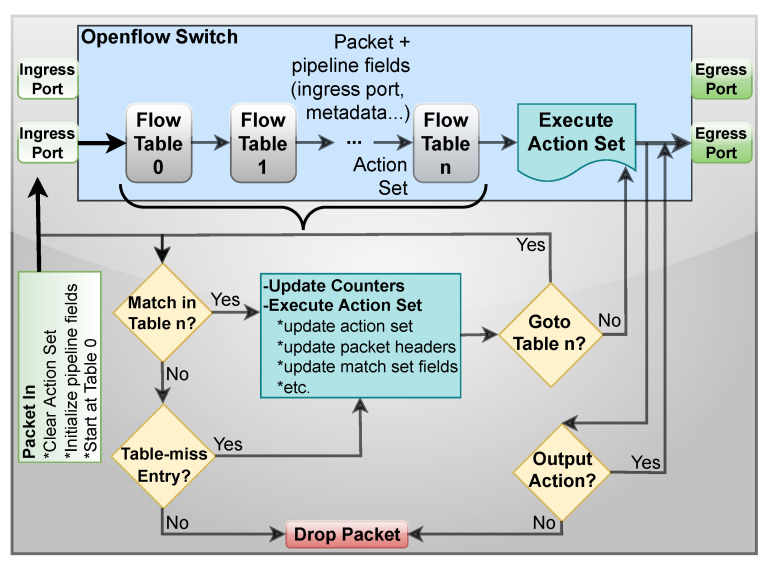
OpenFlow packet processing pipeline illustrating multi-table flow matching, action execution, and forwarding decisions within an OpenFlow switch.

The OpenFlow protocol facilitates configuration and state management through *Controller-to-Switch*, *Asynchronous*, and *Symmetric* message types [73], while packet processing is governed by a pipeline structure indexed from the first flow table (flow-table-0). Figure 4 depicts the internal packet processing workflow of an OpenFlow switch, where incoming packets enter through ingress ports and are sequentially evaluated against a pipeline of flow tables. At each table, match conditions are applied using packet header fields and pipeline metadata; upon a successful match, counters are updated and the associated action set is modified, potentially directing the packet to subsequent tables via a “goto-table” instruction. If no matching entry is found, the table-miss handler determines whether to forward the packet to the controller or drop it. After pipeline processing completes, the accumulated action set is executed to perform operations such as header modification, forwarding to egress ports, or packet dropping, thereby enabling fine-grained, programmable control over data plane behavior.

**Figure 5:**
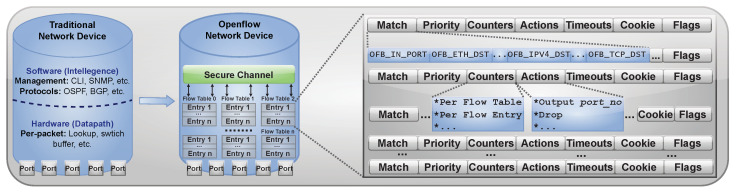
Architectural evolution from traditional network devices to OpenFlow-based SDN switches with flow-table-driven forwarding. Switch, flow table, and flow entry structures in Openflow-enabled SDN networks.

SDN consists of three core elements: OpenFlow switches, external controllers, and the OpenFlow protocol. As depicted in Figure 5, an OpenFlow device comprises flow/group tables, a secure communication channel, and an OpenFlow protocol interface. Each switch maintains multiple flow tables containing flow entries that match and process packets according to predefined rules. A flow entry includes: (i) a *Match* field specifying header attributes (44 fields in the latest specification [73]), (ii) an *Action* field defining operations applied to matching packets, and (iii) a *Stats* field tracking *counters*, *priorities*, and *timeouts* [73]. The controller manages switches over a secure channel (e.g., TLS), receiving events and issuing control commands.

### 3.2. Foundations of Blockchain

**Figure 6:**
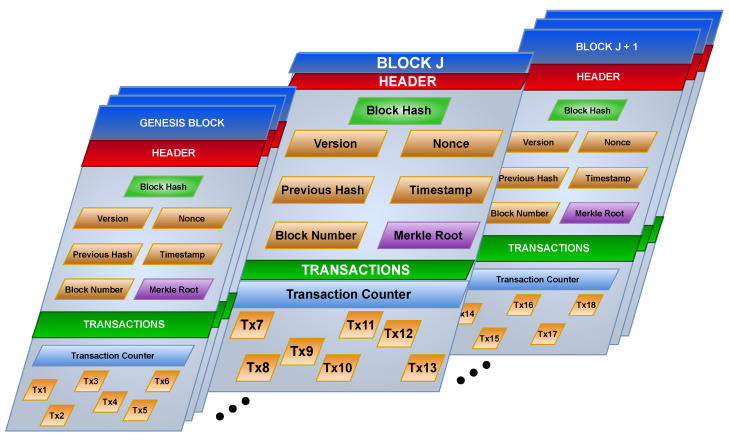
Blockchain block structure and chaining mechanism illustrating header fields and transaction linkage.

Centralized vulnerabilities necessitate the decentralized trust model provided by BC technology. BC functions as a distributed ledger that ensures data integrity and provenance by recording transactions in a cryptographically linked chain of blocks without a central authority [12]. Figure 6 illustrates the internal structure of a BC ledger, showing the genesis block followed by consecutive blocks that are cryptographically linked through hash references. Each block consists of a header containing essential metadata such as the block hash, previous block hash, version, nonce, timestamp, block number, and Merkle root, alongside a body that records a set of transactions and a transaction counter. By embedding the hash of the previous block in the header, the BC ensures immutability and tamper resistance, as any modification to a transaction propagates through the Merkle root and invalidates all subsequent blocks, thereby providing a secure and verifiable distributed ledger. The robustness of this system relies on its core components, including the Block Structure, which contains metadata and verified transaction data. This information is stored in a distributed ledger, which is a synchronized database shared across all network nodes. Cryptographic Hashing links these blocks to ensure data integrity and renders historical alteration computationally infeasible.

Crucially, the system relies on *Consensus Mechanisms*, which are protocols used to achieve agreement among nodes on the validated state of the BC. These include but are not limited to:

*Proof of Work (PoW)* [74]: Validators solve complex computational puzzles to validate transactions and create new blocks.
*Proof of Stake (PoS)* [75]: Validation rights are granted based on the number of tokens or assets a node holds.
*Delegated Proof of Stake (DPoS)* [76]: Stakeholders vote for a group of delegates who are then responsible for validation.
*Proof of Authority (PoA)* [77]: Validation rights are assigned based on a node’s verified identity, placing reputation at stake.
*Practical Byzantine Fault Tolerance (PBFT)* [78]: A consensus algorithm that ensures reliability through a three-phase validation process among primary and secondary nodes in distributed systems.

BC implementations are categorized by access model into *Public* BCs that are permissionless, *Private* BCs controlled by a single entity, *Consortium* BCs governed by organizational groups, and Hybrid models. Platforms such as Bitcoin, Ethereum, and Hyperledger determine core implementation specifics and network architectures. Where supported, a critical functional element is the smart contract, which acts as a self-executing script enforcing agreement terms directly through code. The underlying infrastructure of these systems is built using core development languages such as C++, Go, Rust, and Java, as detailed in Table 3.

The decentralized and cryptographic nature of BC provides specific security advantages for centralized infrastructures. The core security benefits include:

*Decentralization:* Eliminates SPoF, distributing trust across the network and enhancing fault tolerance and system resilience.
*Immutability:* Once a transaction or data record is added to the chain, cryptographic linking ensures it cannot be altered or deleted, which is critical for fraud prevention and forensic data proof [79].
*Trustlessness:* Removes the need for a single, trusted third party to mediate transactions, relying instead on democratic consensus mechanisms for verification.
*Transparency:* All transactions are recorded on a public ledger and are auditable by network participants, ensuring accountability and aiding in the detection of malicious activity.
*Traceability:* Provides a complete, auditable history of all actions and data changes, allowing parties to monitor progress and verify authenticity.
*Anonymity and Privacy:* Users’ identities are protected by cryptographic keys, allowing participants to engage without bias while verifying the ledger publicly.
*Distributed Storage:* Redundancy across multiple nodes prevents data loss, ensures high availability, and provides vast, cost-effective storage for system logs.
*Enhanced Security:* Consensus mechanisms prevent common cyber threats such as fraudulent transactions and double-spending.
*Interoperability:* Facilitates the secure and seamless exchange of verified data between disparate systems and organizations.

Neither SDN nor BC constitutes a singular solution despite their individual strengths. SDN offers high performance and centralized manageability, yet suffers from SPoF vulnerabilities. Conversely, BC provides decentralized trust and immutability but faces scalability and latency limitations. The integration of B-SDN creates a symbiotic relationship in which BC secures the SDN control plane and flow rule integrity, while SDN optimizes BC network performance through efficient routing. This convergence addresses the trilemma of security, scalability, and decentralization in modern networking by combining decentralized trust with programmable management.

## 4. Related Surveys

With the rapid evolution of cybersecurity, BC, and SDN, a significant body of literature has emerged to review their integration. Table 4 presents a comparative synthesis of prior survey studies on B-SDN, systematically categorized by application domain, primary thematic focus, and the specific research perspectives they address.

*General Integration and Architectures:* Foundational surveys often tackle the feasibility, protocols, and broad implications of this convergence. Both security and non-security domains of this integration are explored in [10], while the future of network security is examined through the lens of networking protocols in [11]. A strategic dimension evaluating the feasibility of deployment is added by [12]. Discussions on privacy and convergence with recent technologies, such as AI, are further extended in comprehensive reviews such as [9]. Furthermore, a rigorous security taxonomy categorizing defenses by mechanism, plane, and deployment type is proposed in [22].
*IoT, Smart Environments, and Virtualization:* A substantial portion of existing research investigates the intersection of B-SDN within the IoT. Detailed surveys on securing IoT networks through this combination are provided in [3,4], while state-of-the-art overviews are the focus of [13,14]. This scope frequently expands to include NFV and specific smart environments. Network virtualization in IoT is a theme echoed in the context of smart homes in [18]. The scope is further broadened to smart buildings and grids, synthesizing NFV, SDN, and BC into a secure IoT framework in [16].
*Critical Infrastructure: Transport, Energy, and 6G:* Beyond general IoT, specialized surveys address critical infrastructure and transportation systems. Trust management in VANETs is the primary focus of [5], while specific BC security measures for SDN-based vehicular networks are analyzed in [6]. Softwarization techniques for UAVs are reviewed in [19]. Looking toward future networks, network management for 6G, including UAV case studies, is examined in [8]. Similarly, the synergistic integration within the Internet of Energy (IoE) ecosystem-covering Smart Grids, electric vehicles, and Peer-to-Peer (P2P) trading-is reviewed in [7].
*Threat-Centric Approaches:* Finally, several surveys adopt a threat-centric perspective, particularly focusing on specific attack vectors and mitigation strategies. DDoS mitigation strategies within SDN and IoT contexts are analyzed in [17,20], respectively, while a specific overview of attack detection in SDN is provided in [21].

Existing surveys operate within isolated domains, including IoT, Smart Grids, VANETs, and UAVs, or focus on specific attack types such as DDoS. While these studies establish foundational knowledge, a deeper comparative analysis reveals critical analytical gaps that this manuscript uniquely addresses. For example, although [20] presents valuable defense taxonomies or attack coverage, it lacks a comprehensive, cross-domain attack–defense mapping that systematically links diverse cyber threats to specific B-SDN mitigation, detection, and prevention schemes. Furthermore, previous surveys consistently omit an empirical maturity assessment. Studies such as [3,10] discuss B-SDN solutions theoretically but fail to evaluate the implementation readiness of these frameworks—leaving a gap in distinguishing between conceptual frameworks, simulation-based studies, and real-world deployments. Additionally, while some reviews like [9] introduce AI integration, they do so peripherally, without a dedicated analysis of how machine learning algorithms actively optimize B-SDN security mechanisms. Finally, prior works rarely synthesize performance and implementation metrics, an omission that obscures the critical trade-offs between Blockchain cryptographic overhead and SDN routing throughput.

By directly addressing these shortcomings, this comprehensive survey distinguishes itself from prior literature. It bridges these isolated domains to delineate the complete B-SDN landscape, prioritizing a dedicated analysis of industry-specific use cases alongside a granular attack–defense mapping. Furthermore, it advances the field by providing a granular classification of mitigation, detection, and prevention schemes explicitly tailored to specific cyber threats within the B-SDN architecture. Additionally, this work offers a critical examination of novel AI methods and their comparative efficacy within the B-SDN paradigm, and concludes with a strategic identification of open challenges and future research directions to guide subsequent studies.

## 5. Preliminaries

**Figure 7:**
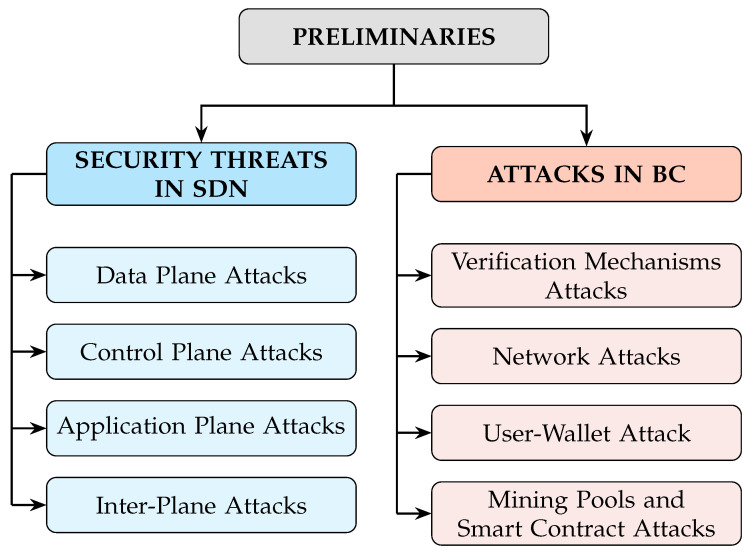
Complementary threat landscapes of SDN and BC motivating B-SDN security design.

The taxonomy shown in Figure 7 highlights how SDN and BC exhibit distinct yet complementary security vulnerabilities. SDN threats predominantly arise from plane separation and centralized control, whereas BC attacks target consensus, networking, and transaction execution layers. By presenting these domains side-by-side, the figure motivates the integration of SDN and BC into B-SDN architectures to address cross-domain attack surfaces. Table 5 presents a set of representative, large-scale cyber incidents observed in recent years, detailing the corresponding attack vectors, impacted assets, incident duration, and the associated operational disruptions and economic losses.

### 5.1. SDN Security Threats

Cybersecurity threats in SDN are typically categorized by the specific plane under attack, necessitating countermeasures such as encryption, access control, and intrusion detection systems.

#### 5.1.1. Data Plane Attacks

The SDN infrastructure is frequently targeted by data plane attacks, with the most prominent being Denial of Service (DoS) attacks and botnet campaigns. Flow tables are often overwhelmed in these scenarios by cyber-criminals sending vast amounts of packets. Network entities can be scanned using packet timing in side-channel attacks, referred to here as probing. Furthermore, non-existent links can be created by modifying data packet contents in topology poisoning, allowing adversaries to launch DoS attacks or execute Man-in-the-Middle (MiTM) attacks [88].

#### 5.1.2. Control Plane Attacks

The most critical vulnerability involves hijacking the controller, as its compromise results in the total loss of SDN control. This vulnerability can be exploited through unauthorized application plane access or DDoS flooding, leading to reduced bandwidth and significant downtime for legitimate users. Since user interfaces reside in the application layer, securing this area is essential for protecting controllers. Vulnerabilities in the application layer can facilitate the insertion of malicious rules, port monitoring, or the deployment of agent nodes to execute MiTM attacks [14].

#### 5.1.3. Application Plane Attacks

Spoofing poses a heightened risk in the application plane, particularly where third-party solutions interface with organizational branches. Network congestion can result from malicious data being fed to unsuspecting applications, while message tampering remains a significant issue due to spoofing capabilities. Furthermore, applications remain frequent targets for DDoS and botnet campaigns.

#### 5.1.4. Inter-Plane Attacks

Communication channels between the control, data, and application planes are targeted by availability attacks to disrupt or degrade interactions. Traffic flows can be altered and malicious data injected via MiTM attacks if any malicious entity is captured on a plane. Finally, inter-plane communication is significantly affected by DDoS attacks, which can affect all the previously mentioned layers [21].

### 5.2. Attacks in Blockchain

Despite its security utility, BC remains vulnerable to numerous attacks. Significant recent examples include the 2024 WazirX attack, where USD 230 million was stolen via malicious packets; the 2022 Nomad Bridge Hack, which resulted in a USD 200 million loss due to a replay attack, and the Squarespace DNS hijacking [82]. In the paragraphs below, these and other attack types are categorized and discussed.

#### 5.2.1. Verification Mechanisms Attacks

Vulnerabilities in the verification process are exploited to manipulate the ledger in this class of attacks.

*Double-Spending Attack:* Funds are spent twice by a cyber-criminal who attempts to send funds to a merchant while simultaneously sending the same funds back to themselves, creating a fraudulent BC branch to invalidate the legitimate transaction.
*Finney Attack:* A block containing a specific transaction is pre-mined but withheld by the attacker. Once a merchant accepts the transaction, the pre-mined block is released to invalidate the initial transaction branch.
*Race Attack:* Two conflicting transactions are broadcast simultaneously, with the success of the attack relying on the fraudulent transaction (sending funds to the attacker) being confirmed first by the network.
*Alternative History Attack:* A longer, alternative BC history is created for the payload to invalidate legitimate transactions, functioning similarly to a Finney attack.
*51% or Majority Attack:* Control of the BC is seized by a group of cyber-criminals gaining more than half of the network’s computing power, effectively overpowering the remaining user base.

#### 5.2.2. Network Infrastructure Attacks

The integrity of the network is targeted in this class, opening the door for further malicious activities.

*DoS Attack:* Nodes are slowed down by high-volume traffic consisting of spam transactions or invalid blocks deployed by cyber-criminals.
*Sybil Attack:* Network operation is altered by creating multiple fake nodes with different IP addresses to gain majority influence.
*Eclipse Attack:* A specific target node is isolated to control its incoming and outgoing data packets, allowing the attacker to feed it false information.
*Routing Attack:* Routing information is manipulated to delay communications and potentially facilitate double-spending, reducing overall network performance.

#### 5.2.3. User and Wallet Attacks

Social engineering and malware are primarily used in this class to steal wallet credentials.

*Dictionary Attack:* Credentials are obtained by systematically guessing from a list of commonly used passwords or phrases.
*Phishing Attack:* Credentials are collected via social engineering methods such as scam emails, malicious apps, or fake website login pages.
*Flawed Key Generation Attack:* Cryptographic keys are predicted by exploiting vulnerabilities in the key generation algorithm, a method requiring significant technical complexity.
*Attacks on Cold/Hot Wallets:* Hot wallets are targeted via malware due to constant connectivity, while cold wallets are vulnerable during brief online transfer windows.

#### 5.2.4. Mining and Smart Contract Attacks

Competition is eliminated in mining pool attacks by groups of miners combining efforts to monopolize rewards. Smart contract attacks involve manipulating code terms to exploit logic errors or alter timestamps.

*Vulnerabilities in Smart Contract Source Code:* User privacy and wallets are affected by source code vulnerabilities that allow for race conditions, function manipulation, or the exploitation of randomness within the code.

## 6. B-SDN: Blockchain and SDN Integration in Light of Security

SDN enables on-demand, real-time, elastic network management, and BC provides decentralized anonymity and network integrity. Combining these two worlds, a wide range of cybersecurity applications can be developed. These implementation rationales and research questions, summarized in Table 6, are examined in detail in the subsequent subsections.

### 6.1. Research Motivation and Questions

**Figure 8:**
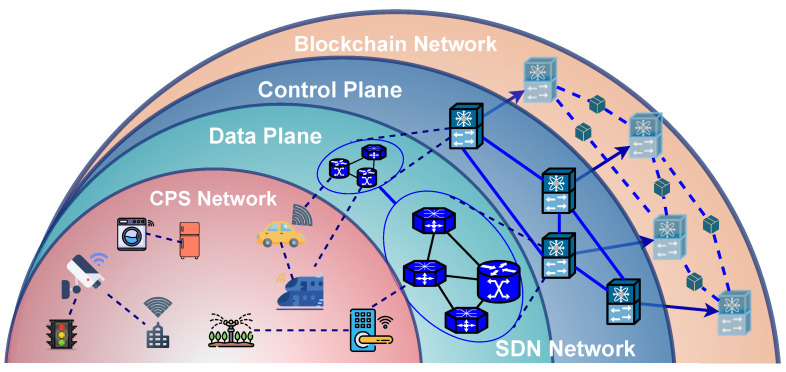
B-SDN architecture for CPS, where SDN and BC jointly act as layered security mechanisms to improve the protection and resilience of CPS devices and infrastructures.

The high-level B-SDN architecture applied to Cyber-Physical Systems (CPSs) serves as the foundational model for this study, illustrating the tight integration of cyber-physical components with programmable networking and decentralized security mechanisms. The architecture depicts the convergence of three distinct functional layers: the underlying *CPS Network*, representing physical infrastructure, cyber-physical devices, and industrial use cases; the *SDN Network*, segmented into the *Data Plane* for programmable forwarding and the *Control Plane* for centralized control logic; and the overarching *BC Network*, which provides decentralized trust, tamper-resistant data management, and auditability, as shown in Figure 8.

**Figure 9:**
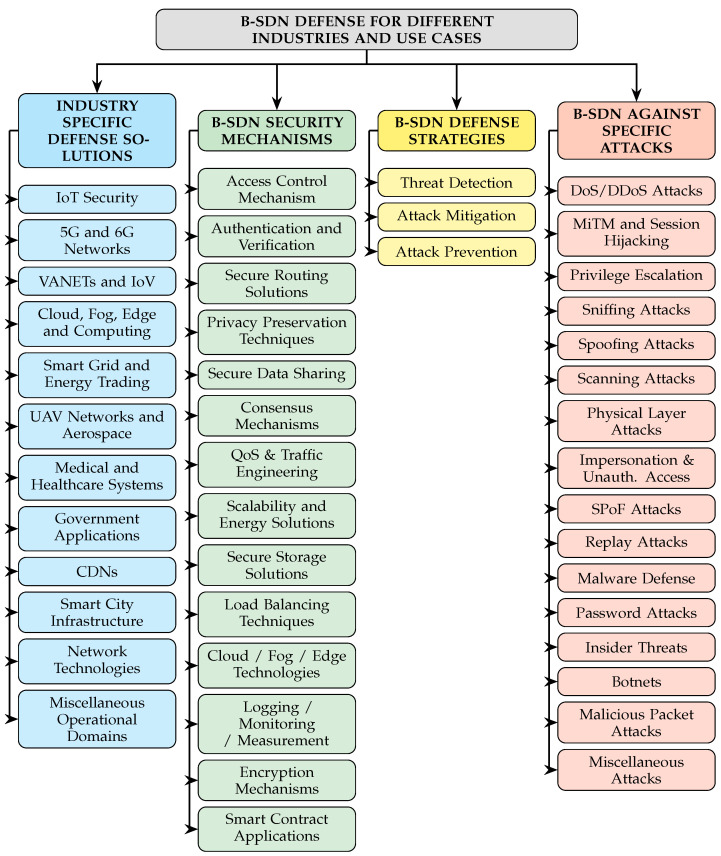
A systematic and threat-oriented taxonomy of B-SDN security and defense mechanisms, linking application domains and use cases with defence solutions, defense strategies, and the cyber attack types addressed in the literature.

The joint use of BC and SDN introduces complementary security layers for CPS and devices. While SDN enables fine-grained traffic control, dynamic policy enforcement, and rapid attack detection and mitigation, BC ensures immutable state validation, distributed authentication, and integrity of control and data exchanges. Together, these mechanisms significantly enhance the resilience, trustworthiness, and overall security of CPS environments against both cyber and cyber-physical threats. The multi-layered structure further highlights critical interaction points where attacks may emerge and where coordinated defense mechanisms can be deployed. Consequently, the following research questions are formulated to address security challenges and optimization opportunities arising from this integrated architecture. Figure 9 presents a systematic, threat-oriented taxonomy of B-SDN security and defense mechanisms, linking application domains and use cases to defense solutions, defense strategies, and the cyber-attack types identified in the literature.

**RQ1: How do the architectural patterns of B-SDN integrations enhance cybersecurity across various industries and domain-specific use cases?**
By asking this question, our survey paper aims to reach researchers interested in novel security technologies in the industry. Given the rapid pace of implementation and recent developments, academic research often attempts to keep pace with industry. Furthermore, specific architectural patterns of B-SDN solutions significantly enhance cybersecurity across various industries by leveraging the mutual strengths of both technologies. As collaborative attack-detection schemes and various cloud-oriented security deployments rise, companies may be interested in novel technologies that affect their revenue.
**RQ2: What are the primary security defense solutions and empirical evaluation practices proposed within B-SDN frameworks?**
B-SDN strives to provide scalability, privacy, and flexibility while integrating cybersecurity. This fusion can be directed to surpass current defense solutions, validated through rigorous empirical evaluation practices. By investigating these possible directions and future work, new research areas can be established. Also, as B-SDN is a relatively new area to explore, new complex problems regarding data integrity, energy, and interoperability have emerged. By presenting this survey, we aim to attract researchers to focus on cybersecurity defense solutions in this area.
**RQ3: How can attack–defense mapping in B-SDN frameworks effectively mitigate and prevent different cyber attack types?**
There are many cyber attacks that need to be addressed, and by asking this question, we aim to map and address the majority of them. This new area of B-SDN cybersecurity, with its ease of use and deployability, may prevent many cyber attacks. Given the vast impact and damage caused by cyber threats, we have systematically established an attack–defense mapping, categorizing different attacks with their respective defense strategies using B-SDN. Both industry and academia researchers can learn from this categorization, and by examining the simulation tools and measurement results in this survey, their combined effort may further deter cyber-criminals.
**RQ4: In what ways does AI-assisted security integrate with and optimize cybersecurity in the B-SDN frameworks?**
AI-assisted security can significantly enhance cybersecurity in B-SDN frameworks. As further sections will show, diverse applications such as automated incident response, improved access control and authentication schemes, network optimization, and real-time threat mitigation frameworks can be optimized with AI. Moreover, there are new technologies that have not yet gained popularity among surveyed papers; their strengths and merits could offer further insights to researchers.

To establish a rigorous analytical framework, each research question is explicitly aligned with the major sections of this survey. RQ1 is addressed in Section 7, which details industry-specific B-SDN defense studies and architectural implementations. RQ2 aligns with Section 8, Section 9 and Section 12, exploring B-SDN security mechanisms, defense strategies, and their empirical implementation and performance evaluations. RQ3 corresponds to Section 10, which systematically maps specific cyber attacks to B-SDN defense architectures. Finally, RQ4 is extensively covered in Section 11, which investigates the role of AI-assisted security within B-SDN frameworks.

### 6.2. B-SDN Integration Architectures

The industry has introduced innovative architectural models that integrate SDN with BC to effectively detect and address various attack types. While the traditional SDN architecture consists of network, control, and application layers, integration with BC frequently alters the connections between the control layer and other components. For example, decentralized architectures use BC to distribute SDN elements by leveraging fog, cloud, or edge computing. In one instance, a secure, distributed cloud architecture backed by B-SDN can address challenges related to anonymity, transparency, and immutability during collaborative cyber attack responses. Similarly, IoT devices connected to controllers in a distributed BC network can leverage smart contract modules to address scalability and energy-efficiency constraints. An architecture integrating AI for secure content distribution may also employ a layered approach for load balancing and storage accuracy, with BC ensuring confidentiality. To implement these security solutions, researchers often deploy new layers on top of the existing SDN infrastructure. The placement of these layers is implementation-specific; for example, in [89], the data and control planes were combined with BC and P4 to reduce the botnet attack surface. Alternatively, another approach proposes that BC adds multiple data planes, divided into blocks, diverging from the traditional SDN scheme [43].

### 6.3. B-SDN Security Perspectives

The combination of B-SDN presents numerous advantages and challenges from a cybersecurity perspective. First, BC provides SDN with a decentralized, immutable data communication platform, enhancing the security of inter-component communication. Within SDN, BC ensures data integrity, reducing the risk of malicious data alteration. Furthermore, the integration enhances network resilience against SPoF in SDN, thereby reducing the attack surface available to cyber-criminals. Conversely, SDN enables rapid, elastic deployment of applications on the BC network. Additionally, BC introduces features such as democratization and anonymity to SDN, expanding the range of deployment options. Collaborative, rapid-response frameworks for attack mitigation or detection are also enabled by the B-SDN fusion [90].

On the other hand, significant cybersecurity challenges persist in the B-SDN integration. First, BC networks inherently face scalability issues, particularly with high transaction volumes, which can impact overall network performance [10]. Another concern is architectural complexity. Integrating BC often expands the system, potentially increasing the number of planes and incorporating cloud-based solutions. This expansion may raise initial investment requirements and overall costs. While BC secures SDN, it also introduces its own vulnerabilities. There are numerous known attack vectors on BC, as detailed in Section 5. Interoperability issues inherent to BC also become a concern in B-SDN cybersecurity. Finally, integrating the vast array of BC applications into SDN requires specialized knowledge and skills from developers.

## 7. Industry-Specific B-SDN Defense Studies

This section explores the diverse landscape of industry-specific B-SDN defense studies, categorizing research efforts by primary operational domain. As B-SDN matures, distinct application patterns have emerged to address the specific security, scalability, and efficiency requirements of various sectors.

The subsequent analysis covers B-SDN implementations across a wide spectrum of fields, beginning with IoT security and extending to 5G/6G networks, Vehicular Networks, Internet of Vehicles (IoV), and cloud/fog/edge computing. Furthermore, the survey examines specialized use cases in Smart Grids and Energy Trading, UAV Networks and Aerospace, Medical and Healthcare Systems, and Government Applications. The discussion concludes with an overview of contributions in Content Distribution/Delivery Networks (CDNs), Smart City Infrastructure, general Network Technologies, and Miscellaneous Operational Domains, illustrating the adaptability of B-SDN architectures in securing critical infrastructure and next-generation services. Table 7 presents a taxonomy of industry-specific B-SDN defense mechanisms, categorizing existing studies across the IoT, 5G/6G, and IoV domains by targeted attack type and representative references.

### 7.1. Internet of Things (IoT) Security

The integration of B-SDN in IoT cybersecurity addresses constraints such as limited resources and reliability requirements by managing defenses through lightweight architectures. Hashing-based security in 5G environments achieves low-latency detection of replay and impersonation attacks [42], while Manufacturer Usage Description (MUD) profiles extended with MSPL enforce privacy and data provenance during bootstrapping [57].

Energy efficiency and resource management are critical in IIoT and smart city contexts. Consensus optimizations, such as PoA and PoS algorithms, reduce CPU utilization and authenticate nodes to mitigate flooding and malicious packet attacks [51,112,120]. Additionally, Virtual Network Function (VNF)-integrated frameworks facilitate energy-aware DDoS mitigation [91], and trust-based traffic filtering protects the control plane’s availability [56].

Scalability and interoperability are enhanced through sharding, encryption, and collaborative learning. Sharding techniques within consortium Practical Byzantine Fault Tolerance (PBFT) improve management efficiency [114], while McEliece encryption ensures integrity with minimal latency [93]. Decentralized collaboration using FL and private ledgers secures learning parameters against DDoS and botnets [90,92,113]. Furthermore, NFV integration detects attacks in smart residential and urban deployments [32,94,95].

Advanced data analysis via AI, digital twins, and hierarchical architectures manages high data volumes and internal threats. Digital twin technology enables packet analysis for flow balance and authentication [53,119,146]. In 6G and edge environments, hierarchical detection using VNFs and decision-driven algorithms optimizes detection speed and energy consumption [97,98,99,147]. Machine Learning (ML) models, including K-Nearest Neighbors (KNNs), Convolutional Neural Networks (CNNs), and Artificial Neural Networks (ANNs), facilitate the classification of tampering, scanning, and intrusion attempts in IIoT [100,101,102].

Specific B-SDN implementations address routing, storage, and forensic challenges. Smart contracts and decentralized identity management reinforce security for P2P and Device-to-Device (D2D) communication [105,110,116]. Hierarchical architectures utilizing PoW isolate rogue switches [96], while fog-based frameworks secure high-volume data [148,149]. Granular anomaly detection is enhanced through Isolation Forest algorithms [111]. Furthermore, specialized mechanisms secure UAV routing, energy-based identification, and forensic evidence against alteration [37,79,103,104,117,118,121,123].

### 7.2. 5G and 6G Networks

The deployment of 5G and 6G technologies requires architectures capable of simultaneously addressing security, robustness, flexibility, and processing speed while operating on lightweight hardware. B-SDN integration provides a viable solution to these multifaceted challenges through decentralized management and programmable control.

Lightweight security frameworks mitigate routing attacks and optimize resource provisioning in 6G infrastructure. Resilience is enhanced through master–slave controller configurations and greedy Lagrangian optimization, which ensure continuous network defense and efficient service management [27,41,127,134]. Furthermore, collaborative decision-making models support secure vehicle scheduling and trust coordination within 5G domains [128].

Authentication and handover management are critical for maintaining trust in multi-operator environments. Tamper-resistant frameworks leverage the immutability of BC to establish trust among SDN controllers and to address DDoS and MiTM threats [30]. Handover delays are significantly reduced by using consensus mechanisms, such as Raft, to validate flow legitimacy during network roaming [125,131]. Additionally, zero-trust models and cellular provisioning architectures embed BC clients to facilitate secure authentication and auditable packet logging [129,132].

Threat detection and mitigation rely on the synergy between B-SDN and NFV to identify suspicious nodes and manage malicious traffic while adhering to latency standards [32]. Security is strengthened by multi-layer Intrusion Detection System (IDS) designs that utilize lightweight algorithms to protect flow rules against overloading [133]. Finally, network resilience and content delivery are improved through VNF-based frameworks that filter traffic to prevent spoofing and alteration attacks while managing trust across disparate regions [97,126,130].

### 7.3. VANETs and IoV

The evolution of transportation systems necessitates prioritizing energy efficiency and security in VANETs. B-SDN architectures provide the programmable control and decentralized trust required to secure dynamic interactions within complex deployment scenarios.

Secure data sharing and traffic coordination utilize 6G technology and distributed consensus to manage data flows. Frameworks employing dual-mode BC and Advanced Encryption Standard (AES)-256 encryption effectively mitigate eavesdropping and spoofing while incentivizing valid data sharing [135,142]. Moreover, these integrations demonstrate significant energy efficiency improvements across diverse vehicular settings [140]. Resiliency in high-density communication is enhanced through distributed fog and edge computing architectures that address SPoF vulnerabilities [38,141,145]. Trust management systems further utilize centralized B-SDN and PoS consensus to enforce penalties for traffic violations and detect tampering in cloud-based reporting [128].

Resource allocation in autonomous and electric VANETs is optimized using VNF and edge computing. These technologies maintain secure connectivity for autonomous units and enable private bidirectional energy transfer between vehicles and Smart Grids [127,136]. AI-driven optimization and metaheuristic algorithms enhance routing efficiency and consensus stability. These approaches prevent Sybil, DoS, and collusion attacks while supporting evidence preservation through cryptographic hashing [137,143,144]. Finally, smart city infrastructures leverage dual-layer BC and bio-inspired trust management to detect malicious traffic and reduce latency in dynamic environments [138,139].

### 7.4. Cloud, Fog, and Edge Computing

The integration of B-SDN within distributed computing environments addresses the critical need for secure resource orchestration and decentralized data management. Table 8 presents an industry-oriented taxonomy of predominant security threats mitigated within B-SDN architectures across the cloud computing, Smart Grid, and UAV sectors, accompanied by representative research contributions from the existing literature.

Cloud security architectures leverage B-SDN to integrate edge and cloud technologies through distributed validation and immutable authentication frameworks. These systems facilitate robust attack detection and ensure data integrity across distributed layers while outperforming traditional OpenFlow-based models in throughput and latency [147,151]. Furthermore, the integration supports digital forensics by providing secure evidence management and predicting privacy leaks in cloud infrastructure [48].

Decentralized access control and resilience against flooding attacks are achieved through multi-controller synchronization and consensus mechanisms. Architectures utilizing PoW and PoS consensus secure control plane functions and fog nodes against DDoS, Address Resolution Protocol (ARP) poisoning, and Internet Control Message Protocol (ICMP) flooding [149,150]. Additionally, Controller-Block models enforce authentication integrity across domains to mitigate SPoF vulnerabilities and privacy concerns inherent in centralized management [153].

In Mobile Edge Computing (MEC) and IoT contexts, B-SDN addresses static security limitations through adaptive resource scheduling and decision consistency. Distributed controllers optimize energy costs and latency for firewall and IDS deployment in resource-constrained environments [152]. Similarly, lightweight security schemes in collaborative defense frameworks ensure efficient operation within cloud-based 5G IoT networks [42].

### 7.5. Smart Grids and Energy Trading

The integration of B-SDN within smart electrical grid infrastructures provides a decentralized framework for managing complex interactions and ensuring the integrity of energy transactions. Secure energy trading relies on this integration to prevent theft and billing manipulation, utilizing automated miner selection and immutable logging for traceability [140]. Privacy-oriented schemes further democratize the grid by establishing BC layers over distributed SDN bases to preserve anonymity, while load balancing is optimized through trusted BC interactions and SDN-based monitoring [155,157].

Network resilience and anomaly detection are enhanced through the synergy of B-SDN, AI, and digital twin technologies. Private BC implementations in smart energy IoT mitigate algorithmic fork problems and SPoF vulnerabilities [39], while digital twins with hierarchical SDN optimization reduce routing latency and facilitate attack detection [119]. Additionally, demand response management utilizing PBFT consensus ensures energy-efficient security, effectively mitigating DDoS, MiTM, and ARP spoofing attacks in simulated environments [136,154].

Comprehensive defense frameworks for critical infrastructure leverage B-SDN and NFV to enable decentralized authentication and automated access control. Multi-layered architectures utilize Ethereum BC for synchronization and SDN for connectivity to connect diverse grid components [31], while integrated NFV solutions ensure flexible threat response [156]. Furthermore, holistic architectural models combine risk assessment, certification, and Federated Learning (FL)-based intrusion prevention to secure management policies and energy transactions against evolving cyber threats [24].

### 7.6. UAV Networks and Aerospace

Securing UAV environments necessitates decentralized trust and programmable control to manage dynamic interactions and ensure operational robustness. B-SDN architectures address these requirements by securing routing and trust management through policy-based forwarding, utilizing distributed ledgers to verify data transmission and mitigate black hole, hijacking, and spoofing attacks [27,37].

Threat detection and privacy preservation are enhanced by integrating AI-driven flow analysis with B-SDN. Centralized controllers manage logging operations while private BC ensures anonymity, enabling the accurate identification of outliers and eavesdropped signals to defend against replay, ransomware, and MiTM threats [27,159].

Architectural resiliency is maintained through fault-tolerant mechanisms that eliminate SPoF vulnerabilities. Master–slave controller configurations utilize BC for secure metadata management to ensure rapid recovery and efficient packet filtering, extending these benefits to broader urban traffic management systems [36,158].

### 7.7. Medical and Healthcare Systems

The healthcare sector utilizes B-SDN to enforce privacy and data integrity across distributed cloud, edge, and Wireless Body Area Networks (WBANs) environments. Architectures integrating hybrid ledgers, sharding, and smart contracts facilitate secure electronic health record management and efficient task offloading, ensuring resilience against sniffing and DDoS threats while maintaining high throughput and traceability [35,160,161]. Furthermore, decentralized authentication schemes utilizing private Ethereum BC protect distributed physiological monitoring devices from replay and spoofing attacks, significantly reducing the operational overhead associated with traditional access control [47]. Table 9 provides a structured overview of the principal security attacks and associated threat categories observed in B-SDN architectures. The information is organized by industry sector, namely, healthcare, government, and CDNs, and is complemented by representative studies that exemplify each identified threat.

### 7.8. Government Applications

The implementation of B-SDN within government sectors enhances the resilience of democratic processes and municipal services. Electronic voting infrastructures leverage decentralized ledgers and network-level monitoring to mitigate physical impersonation risks and guarantee vote transparency, providing a secure foundation for updating legacy systems [33]. Concurrently, smart municipal security architectures utilize hybrid ledgers for encrypted authentication and policy enforcement, where empirical evaluations demonstrate significant throughput improvements and effective mitigation of data forging, replay, and spoofing attacks [162].

### 7.9. Content Distribution/Delivery Networks (CDNs)

CDNs leverage B-SDN to enhance operational trust and secure dissemination through decentralized control. Architectures integrating PBFT consensus and smart contracts facilitate reliable routing, utilizing flexible controllers to optimize latency and mitigate SPoF and malicious packet injection [130]. Furthermore, these frameworks enable secure monetization and equitable resource allocation, ensuring verified trust evaluations and reduced operational overhead [25,130].

### 7.10. Smart City Infrastructure

The integration of B-SDN within smart city ecosystems facilitates resilient urban management by securing traffic optimization, urban sensing, and dynamic resource allocation. Intelligent monitoring schemes utilize decentralized ledgers and SDN controllers to minimize transmission delays and protect data dissemination against unauthorized access and hijacking [36,163]. Furthermore, energy-aware frameworks integrate NFV and edge-cloud interplay to ensure transparency and eliminate SPoF vulnerabilities while efficiently detecting DDoS attacks in 6G environments [91,134]. Table 10 categorizes application domains employing B-SDN, mapping Smart City, Network, and Miscellaneous use cases to the corresponding security attack types identified in the literature.

Specialized environments within the smart city, ranging from agricultural networks to residential infrastructures, leverage these architectures to optimize data exchange and Quality of Service (QoS). Secure packet processing and sensor management in agricultural and residential settings are achieved through BC-as-a-Service (BaaS) and NFV, which protect control planes from flooding attacks and enforce decentralized confidentiality [94,95,164,166].

Advanced intrusion detection and latency management are addressed through the integration of AI and Deep Learning (DL) algorithms within B-SDN frameworks. Hybrid models, including Bi-LSTM and bio-inspired algorithms, enhance network awareness and data integrity, allowing for cost-effective privacy assurance and automated attack logging across diverse IoT domains [122,146,165].

### 7.11. Network Technologies

Specific network technologies leverage B-SDN to enhance internal security and control plane resiliency through decentralized trust models and immutable logging. Trust frameworks enforcing access control at the application–controller interface effectively reduce computational overhead and latency [50], while specialized algorithms optimize anonymity and forwarding efficiency within privacy-preserving networks [173]. Furthermore, control plane consistency is maintained via private PoA or hybrid BC protocols that manage synchronization and operational metrics, providing robust recovery mechanisms against MiTM, spoofing, and flow table flooding [167,169,171].

Distributed collaboration and secure inter-domain connectivity rely on multi-controller architectures utilizing DLT and PBFT consensus to synchronize authorization decisions. These frameworks enable decentralized ABAC logic without centralized authorities, ensuring the non-repudiation of network policies and secure modular intrusion detection [55,168,170]. Additionally, integrated authorization layers fortify controller APIs against attacks targeting centralized structures, thereby establishing a resilient defense against unauthorized policy modifications [168,172].

### 7.12. Miscellaneous Operational Domains

B-SDN implementations address unique operational requirements across wireless, edge, and tactical environments by combining programmable orchestration with decentralized trust layers. Architectures utilizing DPoS and Ethereum ledgers facilitate low-latency data offloading and IoT traffic segmentation, while PoS mechanisms optimize RFID security and FWA performance through encrypted privacy enforcement [26,46,129,176]. Furthermore, resiliency in industrial and military CPS is enhanced through automated switch registration and DL-based anomaly detection, which effectively mitigate DDoS and saturation attacks in resource-constrained settings [146,174,175].

In the sectors of finance and digital forensics, B-SDN enables intelligent evidence collection and anomaly monitoring through Reinforcement Learning (RL)-based routing and secure chain of custody management. Financial business activities are secured by SDN-based communication monitoring that triggers alarms upon identifying malicious nodes [148]. Similarly, cloud-based forensic architectures leverage these frameworks to intercept suspicious traffic, while the BC ledger ensures the integrity of collected evidence throughout the legal chain of custody [178].

Emerging environments such as the Metaverse and microservice architectures utilize B-SDN for request orchestration and decentralized authentication. The integration of AI structures, including CNNs and multi-layer filtering, with Hyperledger enables flexible storage and secure updates to flow rules, thereby maintaining confidentiality and integrity [177,179]. These frameworks effectively prevent tampering, MiTM, and repudiation attacks, allowing peers to access virtualized services with enhanced reliability.

## 8. B-SDN Security Mechanisms

While SDN offers global network visibility and programmable control, its centralized nature can introduce vulnerabilities such as SPoF. BC counteracts these weaknesses by establishing a decentralized, immutable ledger for trust management, auditability, and verifiable consensus. This section explores the diverse security mechanisms within this integrated ecosystem, ranging from fundamental access control and secure routing to advanced applications such as smart contracts and privacy preservation.

### 8.1. Access Control Mechanisms

Restricting access to critical network elements is a central theme in B-SDN integration. A robust scheme combining distributed SDN data forwarding with PBFT verification and attribute-based encryption proves effective against DoS, MiTM, and SPoF risks [29], while a subsequent upgrade incorporating certificate-based access further improves defenses against impersonation and privilege escalation [180]. Decentralized interface security is similarly achieved by offloading authentication to a private Hyperledger BC using chaincode and Crash Fault-Tolerant (CFT) consensus [181]. A structured overview of the threat landscape is presented in Table 11, which correlates critical vulnerabilities with the specific B-SDN countermeasures proposed in recent studies regarding access, verification, and routing.

Immutable user authentication can leverage smart contracts, with SDN handling attributes and Hyperledger RAFT ensuring auditability [186], a concept also relevant to digital twins [53]. For dynamic filtering, P4 integration allows distinct token encapsulation within a Hyperledger consortium BC to prevent malicious terminal attacks [182]. In IoT domains, trackable policy management is facilitated by a private PoA Ethereum BC [187], while latency is reduced by separating authorization from the call process using a lightweight BC [183]. Additionally, device bootstrapping security is enhanced by enforcing extended MUD profiles via Hyperledger Fabric [57].

Addressing IoV scalability, BC sub-networks are deployed in geographically restricted areas to optimize certificate and request handling [188]. Furthermore, AI at the cloud-edge specifically facilitates attribute-based management to mitigate DDoS attacks [184]. Recent adaptive frameworks include Distributed Attribute-Based Access Control for Software-Defined Wide Area Network (SD-WAN), which uses smart contracts for decentralized policy enforcement [168], and cross-domain mechanisms specifically designed to secure SDN APIs [172]. Other specialized contexts include Smart Grids [24], storage-integrated authentication [45], UAV systems [27], and cloud-based schemes for DDoS mitigation [150,185].

### 8.2. Authentication and Verification

Verification management becomes tamper-proof by using B-SDN in tandem. Low-latency verification for smart cities and IoT utilizes “VeidBlock,” where SDN manages dynamic flow rules and BC maintains anonymity via tamper-proof IDs [26]. This system leverages NFV for identity tasks to prevent impersonation, a goal shared by digital twin technologies in IoT [53]. Similarly, cloud data security is enhanced by the “SAUSA” framework, using SDN for forwarding and a private Ethereum PoW BC for integrity to mitigate collusion and DoS [45].

Privacy can be preserved by moving verification off-chain, where SDN handles forwarding and a Hyperledger consortium BC ensures transparency [189]. In 5G contexts, authentication handover risks are mitigated using a private hybrid BC for validation and SDN for network monitoring [125]. This approach integrates DL to determine mitigation strategies against replay and DDoS attacks, utilizing advanced algorithmic optimization. Multi-domain trust in 5G/6G is further addressed by the EDISON framework, which implements a zero-trust model using embedded BC clients for secure key exchanges and smart contracts for Service Level Agreement (SLA) validation [132].

In IIoT sectors, security by contract and MUD profiles govern access, using a private Ethereum BC for immutable data storage and SDN for connectivity to detect phishing and botnets [191]. For data plane security, the DPSec protocol employs a consortium Hyperledger BC to authenticate switch–host communications against spoofing [190]. A refined iteration of this scheme subsequently optimized transaction timing between switch and host components to further enhance performance [198].

Scalability in IoV authentication is achieved by deploying interconnected BC subnetworks in specific geographical areas rather than at a global scale [188]. Related architectures are detailed in separate sections regarding IoT [42], DDoS [126], and load balancing [49]. Finally, smart municipal city frameworks utilize SDN for forwarding within verification systems [162], while distributed SDN environments employ consortium BC smart contracts to establish node identity and data integrity [192].

### 8.3. Secure Routing Solutions

Routing calculation requires carefully designed architectures to manage the influx of entities in modern ecosystems. In IIoT sectors, flexible routing combining SDN and NFV with a private BC ensures trust and tamper resistance against sinkhole, on-off, and selfish attacks [34]. To provide resilience against link failures and DoS attacks, smart contracts can be organized to synchronize distributed SDN management and perform reliable path calculations [193]. For programmable data planes, the PathSec framework integrates P4 to ensure packets adhere to specified routes, using smart contracts to prevent path deviation and forwarding inconsistencies [196].

Scalability and decentralization are addressed through Artificial Neural Network (ANN)-based anti-DDoS schemes where a private BC optimizes traffic payload across multiple SDNs [102]. Similarly, intelligent recovery frameworks utilize ANN for real-time DDoS detection alongside the TOPSIS method for optimal path selection, ensuring rapid link recovery in SD-IoT networks [194].

Energy-efficient architectures combat malicious IoT devices by using DL for detection and meta-heuristic algorithms, such as Spider Monkey Optimization (SMO), for routing [118]. Other domain-specific solutions focus on QoS-related trust [40] and routing security for UAVs [37].

Finally, routing security and load balancing are boosted by monitoring systems where SDN processes information and a hybrid BC ensures robust storage [195]. Further efficiency is gained by combining hierarchical SDN flow updates with BC-based verification to decrease network overhead [197].

### 8.4. Privacy Preservation Techniques

Digital forensics and evidence integrity in cloud environments are secured by integrating B-SDN, where DL handles encryption to ensure accountability and facilitate forensic analysis [48]. To bridge privacy gaps between these technologies, geographically distributed SDNs can leverage the Tangle BC framework to route information securely while preserving privacy [199]. Table 12 synthesizes prior work by organizing reported BC attack vectors into component-level categories and industrial use cases, namely, privacy, data sharing, and consensus mechanisms, enabling a comparative analysis of security challenges across industrial deployments.

Safety in wireless networks is ensured by a Delegated PoS public Ethereum BC, where SDN orchestrates mitigation via smart contracts [200]. This model rewards efficient use of computing resources and addresses a wide range of privacy needs, effectively preventing impersonation, MiTM, and DoS attacks. Similarly, monitoring systems can enhance privacy by utilizing a hybrid BC for robust storage and SDN for processing [195].

Beyond core architectures, domain-specific studies address unique privacy requirements. Smart Grid and electric vehicle privacy is explored in [24,136], while distributed energy trading schemes implement anonymous transactions to hide buyer and seller identities [157]. In mobile networks, privacy solutions are discussed in [26]. Finally, IoV frameworks protect real-time cloud-based video reporting by employing forged digital signatures and symmetric encryption to conceal the identities of uploaders [128].

### 8.5. Secure Data Sharing

Addressing privacy and security concerns in data sharing is essential for ensuring the confidentiality of the entire system. Such solutions must be lightweight and efficient—qualities that the integration of B-SDN can achieve simultaneously. Privacy-sensitive data sharing at edge nodes is secured by the BCDS-SDN framework, where SDN filters management tasks and a Hyperledger public PoW BC handles authentication against DDoS and MiTM attacks [201]. Similarly, D2D communication utilizes pervasive edge computing with a Hyperledger Consortium B-SDN to manage flow forwarding, guaranteeing privacy via cryptographic exchange [202].

In VANETs, secure exchange is facilitated by Natural Language Processing (NLP) driven by RL to filter signals, combined with voting consensus to protect location and identity [142]. For collaborative defense, DDoS mitigation across multiple SDNs is achieved by managing suspect lists via BC smart contracts [203]. This concept extends to Cyber Threat Intelligence (CTI) sharing, where permissioned Hyperledger Fabric BC provides a tamper-proof mechanism for exchanging intelligence to defend against zero-day exploits [204].

### 8.6. Consensus Mechanisms

A gap exists for consensus solutions specifically tailored for B-SDN cybersecurity. To address this, a modified PBFT mechanism is proposed for IoV, facilitating democratic leader election and evidence collection while offering strong platform tractability [143]. In multi-controller environments, secure inter-domain connectivity can be similarly facilitated by Byzantine Fault Tolerance (BFT), where SDN controllers act as validator nodes to ensure data integrity without a central authority [170].

Controller integrity is further maintained through intelligent strategies that operate under attack. A master–slave controller switching strategy can resist SPoF and rule injection attacks through a novel consensus approach, though real-world validation remains limited [28]. Alternatively, the BMC-SDN framework addresses topology inconsistency via a voting-based consensus on a private MultiChain BC [205]. This system integrates a reputation mechanism to adaptively rate and penalize malicious controllers, ensuring flow consistency superior to traditional deployments.

For IoT efficiency, malicious node detection is optimized using novel consensus algorithms that achieve high DDoS detection rates while outperforming traditional PoW and PoS in throughput [32]. Resource consumption at the edge is further minimized by a specialized PoA algorithm that eliminates resource-intensive mining, thereby reducing CPU utilization [112].

To enhance scalability in VNF architectures, prioritized DPoS can be embedded to actively respond to threats, ensuring network availability against malicious nodes [97]. A similar focus on reliability is found in the modified DPoS mechanism, where SDN handles coordination via smart contracts and the consensus protocol implements an election process to remove untrusted SDNs [200]. This effectively mitigates impersonation and MiTM attacks while maintaining an efficient, scalable consensus loop.

### 8.7. QoS and Traffic Engineering

QoS and traffic engineering are critical for secure B-SDN operations. A privacy-prioritized monetization scheme for Internet Service Providers (ISPs) and users can be established where SDN ensures minimal jitter and latency, while a public PoW Ethereum BC guarantees the transparency of smart contracts [25]. This architecture employs a game-theoretic approach to mitigate malicious behaviors, such as tampering and repudiation, effectively. Table 13 presents a systematic mapping of security challenges to critical infrastructure performance, detailing how B-SDN solutions mitigate attacks such as DoS and tampering within the specific contexts of QoS, network scalability, and storage systems.

In vehicular environments, secure V2V transactions are improved by edge-computing frameworks that leverage SDN to enforce global policy and a public BC to ensure trust through reliable value calculation [38]. Similarly, AI-integrated schemes in VANETs enhance QoS by using SDN for energy-efficient bandwidth optimization and PoW BC for fast routing [137]. This optimization specifically targets Sybil and DoS attacks by managing system parameters in a QoS-aware manner. Furthermore, SDN can organize packet processing to gather information from neighboring edge nodes for fast verification, significantly improving execution time against data forging [145].

To secure end-to-end service quality, traffic can be routed based on domain verification results and trust scores [40]. In this setup, SDN handles coordination while a private Ethereum BC manages storage and immutability. This approach is particularly effective against BC-specific threats, successfully preventing Sybil attacks, ballot stuffing, and bad mouthing. Finally, specific QoS considerations for Smart Grids are detailed in [24].

### 8.8. Scalability and Energy Solutions

VANETs face significant challenges in terms of energy and cost. To address this, authentication and access control can be managed through a hierarchical SDN structure where private Hyperledger BC sub-networks ensure flexible scalability regarding CPU utilization and latency [188]. Similarly, general network resource constraints are addressed by scalable architectures in which SDN performs virtualization, and a private Hyperledger BC mitigates transaction loss and SLA attacks [207].

Diverse technologies can be combined to optimize performance. For instance, the configurable nature of P4 and IOTA is integrated to detect attacks, utilizing SDN for packet dissemination and an Ethereum hybrid PoS BC for secure reporting to ensure low-power consumption [206]. In IoT networks, energy and security are optimized by placing a hybrid PoW Ethereum BC between SDN controllers and devices to handle authentication and reduce delay [123], while energy-efficient trust-based classifiers reduce harmful traffic [120].

For smart condominiums and cities, efficiency is managed via cluster head selection algorithms that prioritize high-energy nodes, while NFV reduces physical demands to mitigate DDoS attacks with low CPU usage [94,95]. Finally, domain-specific solutions include Smart Grid scalability [24] and energy reduction via Directed Acyclic Graph (DAG) PoA BC combined with SDN flow rule load balancing [197].

### 8.9. Secure Storage Solutions

Spreading files across a network of nodes offers a powerful tool against cyber attacks by ensuring privacy and scalability. Beyond reducing throughput bottlenecks and authentication handovers, this approach achieves a high level of decentralization. Security in cloud storage utilizes centralized SDN for optimization and BC for trusted access control to specifically mitigate DDoS attacks [185]. For distributed authentication and role-based rights management, data uplink storage employs SDN for network management and a Hyperledger private PoW BC to prevent tampering and MiTM attacks [208]. Similarly, database frameworks secure edge nodes by using SDN for filtering and a Hyperledger public PoW BC to ensure confidentiality in data sharing [201]. The security of data sharing among database servers was validated on an OpenDaylight and Mininet testbed.

Computational resource management and storage are further addressed by scalable frameworks utilizing smart contracts to automate secure allocation and ensure data integrity [209]. Other domain-specific storage applications include Smart Grids [24], authentication mechanisms [45], and transparent trust management logs [117].

### 8.10. Load Balancing Techniques

Load balancing in 6G can be managed by NFV-assisted B-SDN architectures where a DAG BC with PoS consensus handles network slicing and secure handovers [126]. This approach integrates Generative Adversarial Networks (GANs) for slicing, hybrid neural decision trees for packet classification, and Markov decision processes to mitigate Replay and DDoS attacks. Similarly, load balancing can be combined with honeypot mechanisms using Honey Badger optimization, where packet validation at edge servers detects vulnerabilities, and RL mitigates threats such as spoofing and impersonation [49]. Table 14 outlines the threat landscape across advanced network architectures, correlating specific vulnerabilities, such as DoS, Sybil, and tampering, with B-SDN defense strategies in load balancing, distributed cloud environments, and forensic logging.

In smart city environments, the polymerization of NFV and IoT leverages distributed SDN for low-cost management, while BC handles security workloads to mitigate DoS attacks [95]. More recently, secure vehicular communication employs a dual-layer BC architecture with SDN and NFV, using an unsupervised Isolation Forest algorithm at the edge to detect malicious traffic and minimize latency [138].

Complex environments such as Software-Defined Wireless Sensor Network (SDWSN) benefit from energy-efficient load balancing using DAG PoA BC, where hierarchical SDN updates flow rules and RL algorithms perform dynamic routing to prevent impersonation [197]. Finally, specific layer applications include mobile network optimization [26], securing SDN control-data plane interactions to prevent DDoS [43], cloud-based mitigation strategies [185], and flow updates based on Jaccard similarity coefficients [155].

### 8.11. Cloud/Fog/Edge Technologies

Cloud, fog, and edge solutions often form the backbone of B-SDN security. For instance, smart contract security can be implemented using OpenStack cloud integration, where SDN automates forwarding and a private Ethereum BC ensures availability against password attacks [52]. Similarly, secure cloud management with autonomous bandwidth alteration can be achieved via multi-SDN data planes secured by Ethereum BCs [211].

Integrating fog, edge, and AI significantly enhances attack detection in IoT. In such models, SDN analyzes traffic while fog/edge layers reduce complexity, allowing AI to learn detection models via DL to mitigate flooding attacks [210]. This stratification can be further refined by having fog nodes detect attacks and edge nodes mitigate them, using hierarchical SDN for monitoring and a private Ethereum PoS BC for identification [98].

Cloud and edge combinations also detect complex anomalies. AI can be trained to recognize intricate patterns, while attribute-based access management identifies anomalies; in this setup, SDN handles packet processing, and Hyperledger BC manages reputation to prevent impersonation and DDoS attacks [184]. Alternatively, detection can occur at the cloud level with mitigation at the edge, leveraging SDN for flow redirection [147].

Finally, specialized roles for these technologies include using edge for statistical packet access in collaborative DDoS mitigation [203], or for secure manufacturer fingerprinting in IIoT [191]. Cloud storage networks benefit from SDN-based load balancing for reliable accessibility [185], while geographically diverse DoS alert systems utilize cloud and P4 technologies [206], and IoT edges facilitate task placement for DL-driven architectures [215].

### 8.12. Logging/Monitoring/Measurement Techniques

Logging is a critical defense against cybercriminals who attempt to conceal evidence by altering log files. To prevent this, secure network forensics frameworks utilizing decentralized SDN and private Hyperledger BCs have been developed to ensure immutable traceability with low CPU utilization [213]. Forensic security is further enhanced by dynamic packet parsing, in which flow table rules initiate the process and a neural-fuzzy algorithm performs classification [79]. In this architecture, a Bitcoin PoW BC secures storage and verification, facilitating faster processing and accurate detection of forged packet injections.

Securing the SDN northbound interface is equally critical. Authentication and monitoring solutions backed by Hyperledger CFT BCs can be deployed to guarantee immutable decentralization, protecting controllers against malicious applications and mitigating risks such as SPoF, repudiation, DoS, and privilege escalation [212].

To address the high communication overhead of fine-grained monitoring, traffic measurement approaches can employ the ARIMA model for forecasting [103]. By intelligently deciding when to collect statistics, these systems reduce signaling overhead while using the BC as a tamper-proof ledger to record flow statistics, effectively solving measurement inefficiency.

In multi-controller deployments, flow control inconsistency and accountability are addressed by the B2-C2 framework, which integrates BC with digital signatures to ensure secure synchronization of flow rules [214]. This architecture guarantees controller–state consistency and can rapidly replace unresponsive controllers to protect against compromised nodes. Finally, specific monitoring mechanisms for detecting electrical power theft are integrated into broader prevention schemes in [24].

### 8.13. Encryption Mechanisms

While encryption often supports privacy in B-SDN research, it can also serve as the central component for evidence provenance in cloud environments. For instance, evidence can be preserved using SHA-256 hashing optimized by a harmony search metaheuristic algorithm, while data transfer is secured via the Elliptic Curve Integrated Encryption Scheme (ECIES) [216]. In this architecture, a centralized SDN manages authentication and a private PoW BC with smart contracts handles storage, utilizing a Logical Graph of Evidences mechanism to facilitate reporting. Similarly, SHA-256 encryption can be combined with Serpent-based Ethereum smart contracts to address cloud security issues [52]. Table 15 summarizes these findings, classifying the proposed defense mechanisms into encryption-based and smart contract-based solutions against major security attacks.

In vehicular settings, privacy is enhanced by lightweight protocols that leverage elastic decoupled SDN to manage scheduling with the assistance of a modified PBFT consensus [143]. By integrating Elliptic Curve Cryptography (ECC) and SHA-256, this system achieves high levels of protection against MiTM, impersonation, Sybil, and RF jamming attacks. Furthermore, VANETs security can be upgraded using the AES-256 standard to combat DoS, spying, and masquerading [135]. This approach significantly improves data reliability and reduces energy consumption while maintaining throughput.

In IoT frameworks, faster encryption is achieved using the SHA-256 and McEliece algorithms, which are specifically used to increase speed, reduce CPU utilization, and minimize step sizes [93]. In SDWSN environments, unwanted flows can be halted by adopting the Camellia Encryption Algorithm within a DAG PoA BC architecture that also uses AI for dynamic routing based on network status [197]. Finally, attribute-based encryption provides flexible authentication to ensure tamper resistance and prevent unauthorized resource calls, supporting a secure, delay-free authorization model [183].

### 8.14. Smart Contract Applications

Smart contracts often serve as the primary mechanism for enhancing SDN scalability and reliability. For instance, authorized access and DoS protection can be achieved by embedding smart contract solutions directly into SDN rules within a private Hyperledger BC [217]. Network security automation can be achieved by combining smart contracts with intent-driven networks [218]. This approach uses a FISCO consortium PBFT BC to store snapshots and secure the infrastructure, with smart contracts facilitating update messages to switches to minimize consensus time and policy delay.

In IIoT, the SDN control layer is secured against DDoS by uploading detected attack instances to smart contracts using tri-entropy information theory [104]. While SDN decides dynamic flow updates, Ethereum-based Solidity smart contracts provide secure storage for attack records. Manufacturer cybersecurity is enforced by verifying device compliance with usage description policies via smart contracts [191]. This framework focuses on mitigating phishing, impersonation, and botnets by governing access through a private Ethereum BC while SDN ensures connectivity.

Controller coordination and integrity are major focus areas. Insider compromises can be prevented by creating a private controller space via smart contracts using PBFT consensus to reduce overhead [169], a concept related to collaborative intrusion detection [55]. To minimize controller load, data sharing and Certificate Authority verification can be mediated by smart contracts, which hold public keys for gateways and switches [190]. A refined version binds hosts to specific addresses to further reduce delay [198]. Lifecycle management of controllers is automated by architectures that register authentic controllers and blacklist malicious ones via Solidity smart contracts [222]. Similarly, cross-network trust is enforced by smart contracts that transform trust issues into jointly maintained ledgers, enabling cross-chain execution for resource management [221].

Smart contracts significantly bolster collaborative defense capabilities. They secure control and data layer communications against MiTM attacks by transparently examining traffic [30], facilitate the sharing of DDoS attack information [220], and manage scheduling between SDNs to prevent malicious terminal attacks [182,203]. Broader collaboration features such as trust and transparency support AI-based mitigation strategies [219], while other architectures use smart contracts to manage transactions within complex B-SDN-enhanced fog, edge, and AI environments [210].

Finally, smart contracts are deployed for specialized domain functions. Border Gateway Protocol (BGP) security, including hijacking prevention, is conducted by validating data in a Dendrimer tree structure where smart contracts implement route export verification rules [223]. In Smart Grids, smart contracts manage secure energy transactions and data communications [136], or serve as auxiliary tools for parsing network metadata [155]. Other applications include defining access rules and penalties in IoT [187], enhancing routing resilience by uploading topology information [193], automating 6G vehicular payments [127], accelerating terminal access control [186], and bolstering privacy via SHA-256 encryption in Serpent-language contracts [52].

## 9. B-SDN Defense Strategies

The B-SDN provides a robust framework for fortifying network security through decentralized trust and programmable control. This section systematically categorizes B-SDN defense strategies into three distinct operational domains: threat detection, attack mitigation, and attack prevention. By leveraging SDN’s global visibility for real-time monitoring and BC’s immutable ledger for secure accountability, these strategies offer comprehensive solutions to identify anomalies, neutralize active threats, and proactively harden infrastructure against evolving cyber risks.

However, when evaluating the efficacy of these defense strategies, it is crucial to consider their empirical maturity. As highlighted in the subsequent comparative analysis, the vast majority of the current literature validates these mechanisms through simulation environments or constrained prototype testbeds. While these studies demonstrate robust potential against complex threats, large-scale, real-world deployments remain exceptionally scarce. To make this distinction explicitly clear, the current empirical maturity of each proposed defense is categorized directly within our analysis.

The following subsections analyze the recent literature within this taxonomy, evaluating the architectural mechanisms and performance efficacy of these integrated defenses. Table 16 gives a comprehensive comparison of security threats, attack types, and corresponding detection, prevention, and mitigation mechanisms in B-SDN, highlighting the supported security properties, SDN functionalities, validation maturity, and additional system-level aspects addressed in the literature.

### 9.1. Threat Detection

Threat detection within B-SDN architectures leverages the global visibility of SDN and the immutable record-keeping of BC to create robust monitoring frameworks. ML and FL are frequently integrated to enhance detection accuracy across critical infrastructures by narrowing the threat search spectrum. For Smart Grids, hybrid solutions combine FL for intrusion detection with ML-based malware analysis to facilitate risk assessment, certification, and security training [24]. Security performance in these environments is further enhanced by load-balancing schemes that incorporate specialized DDoS detection mechanisms to maintain stability under attack [155].

In the realm of IoT and IIoT, decentralized collaboration helps mitigate the resource constraints of edge devices while ensuring data integrity. Frameworks such as FedChain-Hunter utilize HyperLedger and FL at local edge servers to monitor and filter threats such as botnets and MiTM across multiple organizations [92]. Decoupling SDN from secure BC channels enables early DDoS detection through granular network traffic analysis in IoT devices [99]. Furthermore, honeypot mechanisms employing capsule neural networks and optimization algorithms validate suspicious packets to identify malicious flows with high accuracy [49].

Advanced algorithmic approaches optimize anomaly detection and pattern recognition to address complex attack vectors. DL classifiers deployed in fog–edge architectures identify attacks and store hash values on BC for off-chain assessment [98]. ANN processes network traffic features to distinguish malicious behavior from legitimate flows during data preprocessing and training phases [102]. Similarly, AI models trained on intricate network patterns manage CPU utilization during impersonation and DDoS events [184]. Random Forest algorithms, supported by Principal Component Analysis (PCA) for data reduction, construct effective DDoS detection models in cyber-physical environments [224]. To address subtle attacks, double-layered defenses use Isolation Forests to secure flow tables via BC [111]. Real-time adaptability is achieved by combining CNNs for spatial feature extraction and Recurrent Neural Networks (RNNs) for sequence learning, enabling the system to autonomously adapt to evolving vectors [227].

Structural and protocol-specific innovations also enhance threat visibility. Inserting an intermediate plane between the control and data layers enables the calculation of flow-suspect rates, which are then shared via smart contracts [203]. Tri-entropy theory provides an alternative theoretical basis for similar detection schemes [104]. In the domain of routing security, algorithms such as Dual Agent-based Twin Actor Twin Delayed Deep Deterministic Policy Gradient (DA-T2TD3) secure BGP by identifying anomalies such as duplicate announcements or irregular hop counts [223].

### 9.2. Attack Mitigation

Mitigation strategies in B-SDN architectures employ diverse technologies to distribute defense mechanisms and minimize impact. Collaborative frameworks leverage smart contracts to coordinate DDoS mitigation across multiple domains without reliance on centralized authorities [220]. These decentralized mechanisms facilitate the dynamic exchange of attack intelligence and enable entities to manage their participation in defense schemes flexibly [90]. To enhance synchronization, architectures that use P4 and the public Ethereum BC share attack fingerprints to automate filtering across organizations while minimizing CPU and memory overhead [89]. Furthermore, immutable ledgers enable the rapid distribution of CTI, allowing SDNs to proactively enforce defense policies against large-scale distributed attacks [204]. Specific implementations use smart contracts to disseminate suspect lists across domains, triggering automatic flow table updates for immediate policy enforcement [203].

Advanced mitigation strategies integrate AI and consensus protocols to refine response accuracy and adaptability. FL frameworks built upon distributed SDNs enable the collective training of IDS models to mitigate emerging threats while preserving data privacy by retaining raw data locally [219]. Similarly, ANN-based systems automate the update of SDN switch rules based on real-time detection analysis to neutralize threats [102]. Consensus mechanisms enable the probabilistic filtering of packets to mitigate malicious nodes and DoS attempts effectively [97]. In fog–edge architectures, mitigation responsibilities are offloaded to edge nodes, which activate blocking protocols solely upon detection alerts from fog layers, thereby optimizing traffic flow and limiting resource consumption during normal operations [98].

### 9.3. Attack Prevention

IDS and Intrusion Prevention System (IPS) within B-SDN architectures function, respectively, as passive monitors and active defense mechanisms, leveraging decentralized ledgers and programmable controls to enhance network security. Collaborative IDS frameworks leverage distributed SDN to establish FL foundations, enabling peer SDN to gather intelligence on Remote-to-Local (R2L), User-to-Root (U2R), and probe attacks without exposing private data [219]. To ensure the integrity of this shared threat intelligence, Collaborative IDS leverages BC’s tamper-proof nature to aggregate local alarms, thereby preventing malicious nodes from injecting false reports or manipulating logs [228]. Cross-domain coordinated defense is further achieved through private PBFT Ethereum BC, ensuring synchronization among SDN controllers across geographical boundaries [55].

In specific industrial applications such as Smart Grids, hybrid systems incorporate FL for intrusion detection and ML for malware analysis to monitor TCP/IP flows and application-layer protocols [24]. Similarly, IIoT security benefits from embedding ensemble learning techniques such as Random Subspace Learning (RSL) and KNN to improve attack classification accuracy on industrial control datasets [100]. Broader IIoT frameworks employ CNNs within SDN-based IDS, using BC at the application and network layers to secure training datasets against command or rule injection attacks [101]. Neural networks are also applied to scan global network traffic over extended periods to learn and detect infrequent attack patterns [226].

For specialized environments such as UAV networks, B-SDN facilitates secure data dissemination while AI-driven flow analysis distinguishes legitimate packets from malicious surveillance or eavesdropping attempts, subsequently blacklisting illegitimate traffic [159]. Trust-based traffic filtration mechanisms protect BC nodes from flooding attacks by verifying traffic legitimacy [56]. Additionally, packet classifiers that use graylist and whitelist strategies effectively mitigate MAC flooding attacks in B-SDN infrastructures [120].

## 10. B-SDN Against Specific Attacks

B-SDN combines SDN programmability with BC’s decentralization, immutability, and verifiability to mitigate threats that exploit centralized control and trust limitations in conventional SDN. This section reviews how B-SDN counters specific attack classes—from DoS/DDoS to MiTM, spoofing, insider threats, and botnets—highlighting domain-driven design choices and trade-offs across networking, IoT, 5G/6G, vehicular, Smart Grids, and UAV settings, and complementing the taxonomy in Table 17. The table provides the most comprehensive industry-wise taxonomy of BC-enabled and B-SDN-based solutions, systematically mapping key design objectives (e.g., security, performance, scalability, privacy, resilience, and integrity) to representative studies across diverse domains, including networking, IoT, smart cities, 5G/6G, vehicular networks, IoE/Smart Grids, and UAV systems. The table is organized into three distinct columns to facilitate a cross-referencing of the literature: *Industry*, which classifies the studies into broader sectors such as Networking, IoT, Smart Cities, and 5G/6G; *Goals*, which delineates the specific performance metrics or security attributes targeted by the authors, such as resilience, privacy, scalability, and speed; and *Reference*, which associates these targeted improvements with the corresponding works.

### 10.1. DoS/DDoS Attacks

Mitigation strategies using reputation assessment and immutable PoA Ethereum reduce complexity while maintaining low cost across worldwide AWS instances [54]. Similar reputation-based task offloading strategies are applicable to WBANs [160].

Switch-level threat prevention is achieved by embedding PoW-based BC security into data and control plane interaction channels, ensuring resiliency and privacy [43]. Similar switch-level or master-slave strategies utilizing smart contracts are explored in [28,217]. Secure SDN can also act as a firewall to filter traffic for Multichain applications [229].

Low CPU and memory utilization mitigation schemes are possible using P4 and edge networks, where public Ethereum assists with filtering and VNF creates attack fingerprints [89]. Attack detection at the packet parsing level can be achieved via P4 integration, showing high accuracy against probe attacks [230]. NFV technologies are further applied to DDoS defense in [49,126], while low-power P4 integration for alert systems is proposed in [206].

Dynamic policy pushing without third parties is enabled by collaborative mitigation, in which private Ethereum handles storage and smart contracts manage attack information [220]. In IoT environments, cost-efficient service coordination is achieved through Hybrid Ethereum and Solidity smart contracts [90]. Privacy-preserving FL allows SDNs to collaboratively train detection models for R2L and U2R attacks without sharing private data [219]. Control and data plane solutions that use smart contracts for suspect list sharing and flow table alteration exhibit low CPU overhead [203]. Similar multi-layer attack mitigation strategies are observed in IIoT settings [101]. Cross-domain collaborative defense is further refined by hierarchical architectures that distinguish between intra-domain private BCs and inter-domain public threat intelligence sharing [233].

Early detection in IoT is facilitated by decoupling SDN and using decentralized BC secure channels for traffic analysis [99], with similar approaches seen in [32]. In IIoT, tri-entropy information theory combined with smart contracts offers inexpensive and secure storage for attack recording [104], while broader prevention of flow table overflows is discussed in [100]. Mobile fog and edge computing can employ DL for decentralized attack identification [98]. Rapid response times against ARP poisoning and various flooding attacks are achievable in IoT frameworks [149].

In Smart Grids, workshops address small-scale DDoS attacks on prosumers and aggregators [154], while broader detection performance is examined in [155] and general Smart Grid security in [24]. Maximum efficiency in secure communication is achieved by combining digital twins and AI to detect TCP, User Datagram Protocol (UDP), and SYN flooding [119].

A privacy-preserving defense using BC sharding and Residual Networks (ResNet) leverages distinct datasets for current and legacy traffic to maintain high F1-scores [232]. UAV inter-communication security is examined in [27]. DL on NSL-KDD datasets within a fog/edge architecture ensures accurate detection of TCP and ICMP flooding [210]. Cloud–edge integration using AI pattern detection and attribute-based access control prevents DDoS [184]. True positive rates in detection can be increased by tuning Deep Neural Network (DNN) classifier weights via the Poaching Raptor heuristic algorithm [225]. Random Forest algorithms combined with PCA at the edge effectively construct detection models for cyber-physical environments [224].

In VANETs, Q-learning optimization and sidechain-based BC improve preventive success against Sybil and Smurf attacks by selecting optimal routing paths [137]. AES-256 encryption within the B-SDN framework effectively thwarts attacks [135]. Trust-based models detect malicious activities targeting SPoF [141,202]. A multi-layered defense using CNN, Long Short-Term Memory (LSTM), and Ant Colony Optimization reduces packet loss by 10% and achieves 98% accuracy in preventing flooding and masquerading threats [139]. Optimal routing strategies for general prevention are also noted in [34].

Spoofing attacks are mitigated by decoupling planes and implementing secret-key generation for authentication tasks [29], with random-access restrictions further preventing DDoS [180]. Network staging structures that monitor activity and isolate verified hosts aim to mitigate threats at the handshake stage [190], while optimized versions offer reduced delay through specific MAC binding [198].

Handover schemes in 5G utilize Hidden Markov Models and fuzzy algorithms to detect load-increasing activities [125]. Quick restoration of switching paths via primary and backup path optimization prevents service denial [193]. Secure orchestration in resource-limited wireless environments is achieved without compromising privacy [200]. 6G network resiliency is enhanced by focusing on fast recovery and low loss rates [97].

Intent-driven mechanisms combined with secure storage reduce packet loss rates during flooding attacks [218]. Database-related environments benefit from detection performance focused on data sharing [201]. In cloud storage, privacy-related mitigations ensure efficient CPU utilization [185]. Broader framework applications by the same authors address bandwidth measurement in condominiums [94], low-cost network management [95], stable decentralized load balancing [150], and energy consumption optimization [96]. Smart city architectures combining NFV minimize CPU utilization while preventing SPoF [91]. Real-time detection of malicious traffic in cloud networks is enabled by a Controller-Block model that decentralizes authentication, outperforming traditional models in resilience [153]. Finally, in containerized environments, the AMRA framework integrates the Elastic Stack to identify SYN floods and automate mitigation via REST APIs [237].

### 10.2. MiTM and Session Hijacking

Comprehensive mitigation and detection strategies for wide-scale Smart Grid settings are established in [24]. Protection against hijacking extends to drone communication [27,36], vehicular sessions [38,142], and cellular networks [129]. To counter poisoned SDN elements, application layer behavior can be monitored using trust values that dynamically grant or revoke access [50].

In VANETs, resilience against known MiTM vectors is validated using the AVISPA tool, which secures login phases and secret key generation [127], while vehicle private keys are protected explicitly in [143]. For UAV networks, unauthorized third-party access is prevented by requiring authorized signatures generated within strictly limited time windows [159].

Access control is strengthened through three distinct layers of checkpoint verification, preventing unauthorized entry even with compromised credentials [29,180]. Traceability and authentication between the SDN control and data layers are maintained via smart contracts that audit data to detect interference [30]. Similarly, switch-controller integrity is preserved by storing only legitimate flow rules on the BC, effectively preventing injection attacks [100].

Security in resource-limited mobile environments is optimized through orchestrated B-SDN mechanisms [200]. Confidentiality and integrity in data-sharing environments, such as databases, are addressed in [201]. Technical implementation details and code examples are available in workshop reports [154], while specific implications for microservice security are discussed in [177].

### 10.3. Privilege Escalation Attacks

Research addressing privilege escalation within B-SDN architectures remains limited. However, preventive mechanisms specifically designed for Smart Grid infrastructures have been implemented to mitigate unauthorized access elevation [24]. While the threat of privilege escalation is acknowledged in other QoS-focused studies, comprehensive cybersecurity solutions that address this specific attack vector have not yet been fully developed [25].

### 10.4. Sniffing Attacks

Sniffing attacks focus on intercepting and monitoring network traffic. Mitigation is achieved through data traceability and a layered, energy-efficient edge–cloud cooperative structure that excels in task offloading [35]. Similarly, low-latency mobile environments developed in the Go language effectively secure cloud interactions against sniffing attempts [46]. In Smart Grid settings, shielding against sniffing is often integrated into broader security systems [24,50].

In the domain of aerial vehicles, communication upgrades using B-SDN have been proposed to secure UAV networks [27]. For ground transportation, eavesdropping in Vehicle-to-Vehicle (V2V) or vehicle-to-roadside communication is countered by implementing AES-256 encryption standards [135].

In IIoT environments, eavesdropping and phishing attacks are prevented by using edge and fog computing resources where MUD govern access and network authentication. By disabling unauthorized communication via secure gateways, these attacks can be effectively stopped [191]. For foundational knowledge, workshop reports detailing environmental setups and power-grid attack basics provide a practical entry point for understanding sniffing threats [154].

### 10.5. Spoofing Attacks

Spoofing attacks involve falsifying data to impersonate entities. While architectural enhancements often address message integrity [211] and access control [186], inexpensive upgrades to existing B-SDN systems can also effectively mitigate risks [44]. Specific application domains require tailored defenses: privacy-focused monetization schemes target spoofing in fair trade [25], while low-latency environments utilize robust authentication [26].

In the vehicular domain, mitigation strategies focus on energy trading in automated systems [140] and securing communication to prevent traffic collusion [135,142]. Other specialized examinations extend to the medical sector [47] and cellular networks [129], while cyber-physical environments are discussed in [114,238].

Advanced architectural defenses include three-layer checkpoint verification systems where transactions across all planes are validated by controller-formed blocks [29,180]. Similarly, certificate-based access control validates identity using unique signatures to prevent repudiation and information disclosure, and relies on trust schemes to detect over-privileged entities [181]. For high-performance defense, deploying P4 onto the BC layer provides decentralized security against malicious SDN terminals [182], while integrating NFV and AI with Soft Actor-Critic algorithms allows SDN to predict spoofing outcomes by classifying intruder packets [49,126].

Securing SDN components directly involves enforcing credential immutability on controllers via BC monitoring to stop malicious applications from accessing northbound interfaces [212]. At the switch level, IP and MAC spoofing are prevented by Consortium Hyperledger BCs that bind hosts to specific addresses and ensure data arrive from trusted ports [190,198]. Finally, smart city frameworks utilize Hybrid Multichain BCs to secure notarization and authorization, with SDN handling forwarding authentication to stop data forging [145,162].

### 10.6. Scanning Attacks

Attack detection via packet parsing can be effectively achieved using P4 combined with B-SDN, where the SDN controller handles threat detection and a public BC ensures fast decentralization. Vivado simulations confirm efficient resource allocation, while high detection accuracy and low false alert rates are reported for scanning, DoS, R2L, and U2R attacks [230]. Scanning entities can also be mitigated by monitoring application-layer behaviors via trust scores, which dynamically enable or disable applications based on their operational patterns. This mechanism effectively repels scanning attempts by detecting and isolating unnecessary entity actions before malicious purposes are realized [50].

For foundational knowledge, technical details and code examples regarding scanning attacks in power grid environments are available in specific workshop reports [154]. Finally, reconnaissance attacks in IIoT settings are fully mitigated through attack grouping and joint mitigation strategies within B-SDN frameworks. In addition to scanning, this architecture achieves high detection accuracy for DoS and injection attacks [101].

### 10.7. Physical Layer Attacks

Cyber-physical systems, where physical sensors are most vulnerable, represent a critical area for B-SDN integration. Network virtualization in these environments can be enhanced by learning system attributes via Deep Reinforcement Learning (DRL) to simulate real network environments, thereby achieving high performance and revenue metrics [238]. The security of physical and digital assets in IoT is strengthened through digital twin technology, which increases overall system reliability. In this framework, SDN is equipped with feature extractors, authentication modules, and packet analyzers that assign unique digital signatures to packet headers [53].

Voting fraud is combated through a proposed system that derives physical security from the ballots themselves, while voter records are securely stored within a B-SDN scheme [33]. Finally, the security of physical and digital body data in the medical sector is addressed through task offloading and BC sharding, which specifically target the protection of vulnerable digital assets [160].

### 10.8. Impersonation and Unauthorized Access

Impersonation involves attackers disguising themselves as legitimate entities. In 5G IoT and 6G vehicular environments, multi-factor authentication halts disguised attackers [42,127]. Access control can be secured via bi-variate polynomial functions and pre-generated secret keys [180].

Voting fraud is prevented by stopping impersonation through B-SDN verification [33]. In IIoT, secure gateways governed by MUD disable unauthorized communication [191], while internal threats are mitigated using ML algorithms [100]. Routing dynamics utilize AI and the Camellia Encryption Algorithm to halt unwanted flows [197]. Furthermore, vehicular BC checks prevent Sybil attacks [143], and UAV impersonation is deflected by requiring authorized signatures [159].

Tampering involves harmful data alteration. Defenses include privacy-preserving monetization schemes [25] and preventive actions against collusion [45]. Symmetric encryption and use-time analysis allow abnormal tokens to be discarded to avoid forgery [183]. In medical sectors, BC-based blacklists resist tampering with body data [160], while resource-limited mobile environments achieve resistance without compromising lightweight security [200].

In IoV, message encryption allows malicious alterations to be monitored via BC [143]. The robustness of permission management is enhanced by tamper-resistant BC storage [208]. False Data Injection in multi-controller architectures is mitigated by immutable controller communication [205]. Similarly, routing path integrity is secured by cryptographic signatures and tamper-proof audit records via smart contracts [196]. IIoT tampering is further examined using KNN and RSL algorithms [100].

Unauthorized Access is mitigated by embedding smart contracts into SDN rules to monitor transaction logs [217], or by defining rules and penalties for access detection [187]. Decentralized networks prevent entry by authenticating SDN controllers via BC before communication [31], while command injection is prevented by logging actions on a MultiChain ledger [113]. Finally, unauthorized node access in smart cities is combated using the Secure Hash Algorithm (SHA)-256 and McEliece encryption to reveal attacker locations via topological control checks [93].

### 10.9. Single Point of Failure (SPoF) Attacks

SPoF refers to a critical system vulnerability in which the failure of a single component halts the entire system. Foundational discussions on securing these vulnerabilities within B-SDN architectures are provided in [141,202]. Virtual network embedding effectively detects SPoF while lowering CPU utilization and bandwidth requirements. Simulations indicate that this approach achieves superior fault tolerance compared to non-BC solutions [231].

Secure SDN control planes in IoT settings can be achieved using an energy-efficient Ethereum hybrid BC that optimizes delay, throughput, and energy consumption for access control and authentication [116]. Similarly, sharding techniques are proposed to mitigate SPoF [114], with real-world experiments validating the efficacy of BC-based sharding at the SDN control layer [160]. A two-way master–slave switching strategy aims to mitigate the SPoF by activating a secondary subsystem during attacks, while simultaneously securing rule injection via controller consensus [28]. Alternative switching methods that utilize digital twin technology also offer robust countermeasures [53].

Malicious flow rule modification is detected through hash mismatches in an immutable ledger within a BaaS framework. By leveraging PoS consensus, in which the controller stakes its network topology, this architecture prevents control plane hijacking and mitigates SPoF vulnerabilities in smart city applications [166]. The northbound interface is secured against SPoF, repudiation, and information disclosure by a monitoring system that identifies vulnerable resources and blocks malicious application access via BC [212]. Finally, in CDNs, response times and consensus overhead are minimized through a trust evaluation framework designed to mitigate SPoF risks [130].

### 10.10. Replay Attacks

Replay attacks involve resending intercepted information to gain unauthorized access. While predominantly focused on spoofing, WBAN replay attack dynamics are also discussed in [47]. Secure password protection against replay attacks is achieved via a lightweight, hashing-based multi-factor authentication scheme that uses PoW Ethereum for storage [42]. Alternatively, intruder packet classification using NFV and DAG BC with PoS effectively detects replay attempts [126]. Furthermore, handover authentication combined with edge-based honeypot mechanisms significantly increases the difficulty of conducting replay attacks [49].

Access control schemes that use random number generation and system timestamps enable comparing incoming requests with real-time data, effectively identifying replay attempts [29]. In 5G environments, flow validation and legitimacy checks are enforced by a private hybrid BC, where the SqueezeNet-Fuzzy algorithm detects attacks based on learned packet features [125]. Resource-limited wireless and mobile environments benefit from orchestrated B-SDN mechanisms that mitigate vulnerabilities while preserving privacy, demonstrating superior delay and throughput performance compared to other lightweight schemes [200].

For UAV operations, replay attacks are prevented by requiring timestamped messages to carry authorized signatures, thereby blocking unauthorized broadcasting [159]. Similarly, in IoV settings, message duplication is rendered impossible through the implementation of BC timestamps and threshold concepts [143].

### 10.11. Malware Defense

Research specifically addressing malware within B-SDN remains limited, making it a critical area for future investigation. However, specific detection mechanisms for Smart Grid environments have been established [24]. Additionally, secure communication schemes that use BC to prevent data modification during transfer effectively mitigate the risk of malware injection [164].

### 10.12. Password Attacks

Secure password protection against brute-force and social-engineering threats is achieved via a lightweight, hashing-based multi-factor authentication scheme. In this architecture, packet contents are simultaneously examined using fuzzy neural networks to ensure comprehensive security [42]. Smart contracts and encryption mechanisms effectively combat password attacks, with extensive experimental validation demonstrating the handling of encrypted files and access matrices. These implementations provide detailed code examples for secure file transfer and access control within B-SDN environments [52].

### 10.13. Insider Threats

Insider threats remain challenging, but exposure can be mitigated by employing a trusted third party for entity confirmation and deleting information immediately after registration to minimize compromise [180]. Secure coordination between distant controllers can be similarly established via smart contracts that create a private controller space resistant to internal compromise [169], with specific detection schemes further developed in [55].

In IoV environments, insider Sybil attacks are prevented through dynamic task management and privacy-oriented BC mechanisms that restrict malicious nodes from acquiring complete task information [144]. At the switch level, unauthorized flows and spoofing are practically eliminated through strict MAC and IP verification combined with the isolation of SDN switches from external BC interactions [190,198]. Finally, betrayal attacks by trusted nodes are mitigated using a BC-based trust management model that monitors expert nodes and automatically isolates those sharing untruthful alarms [228].

### 10.14. Botnets

Botnets are collaborative networks of compromised devices under the control of cyber-criminals. Collaborative defense against Mirai-like botnets in IIoT is achieved via a decentralized platform where SDN handles data monitoring and BC facilitates secure contribution among clients. This scheme secures participant identities through encryption and utilizes FL to analyze gathered attack information [92].

A distributed defense shield is formed by synchronizing multiple organizations to capture attack fingerprints using P4-enabled B-SDN. In this framework, P4 handles packet filtering while BC ensures secure synchronization across the participating entities [89]. Feasibility and flexibility in IoT botnet mitigation are enhanced through a collaborative service coordination model using Private Ethereum and SDN. Testbed results confirm that attack source information can be shared between collaborators with reasonable gas consumption and scalability [235].

In IIoT settings, botnet attacks are prevented by enforcing MUD to govern access and network authentication. By disabling unauthorized communication via secure gateways, the system effectively isolates compromised devices [191]. Finally, the threat of botnets in IoV environments is mitigated by implementing strict authentication protocols that require vehicles to verify their identity before entering specific network segments [143].

### 10.15. Malicious Packet Attacks

Defense against malicious packets requires balancing inspection depth with latency. In IIoT, energy-efficient routing utilizes a distributed PoA Vechain ledger to ensure rule enforcement and detect malicious packets [51], while false flow rule injection is prevented using Multichain BC for controller authentication [236]. Robustness is further enhanced by digital twin implementations where packet analyzers examine injected features [53], and by CNN algorithms trained on industrial datasets to identify rule injection and scanning threats [101]. Collaborative defense in edge–cloud frameworks similarly identifies malicious packets by leveraging neighboring IoT devices to reveal unconfirmed nodes [147].

In IoV, injections are neutralized via locational access control that restricts authentication to specific geographic areas [188]. For high-bandwidth video reporting, protection against forgery and malicious scoring is achieved through majority voting, digital certificate cross-checks, and fixed-identity blacklisting [128].

UAV security relies on rapid validation windows and AI flow analysis to deflect message alteration [159], while CDNs employ trust evaluation and proxy selection to mitigate malicious nodes [130]. Conversely, Smart Grid schemes acknowledge vulnerabilities posed by malicious active nodes [157]. Specific concerns in cellular and financial contexts are addressed in [129,148].

### 10.16. Miscellaneous Cyber Attacks

BGP hijacking can be detected using DL models, while SDN ensures optimal routing, and a DAG-hybrid BC facilitates interoperability [223]. This framework integrates Blowfish and ECC to achieve low packet drop ratios. Routing security is further improved by connecting separately operated SDNs via private Hyperledger BC to support dynamic flow rule updates [234], or by frameworks that prioritize bandwidth and jitter optimization over scalability [41].

Broader defense strategies address consistency among controller instances to prevent malicious activity [171]. Finally, zero-day attacks are identified through anomaly-based recognition utilizing ML, where unsupervised learning establishes a traffic normality baseline to detect novel deviations [226].

## 11. AI in B-SDN Cybersecurity

Undeniably, AI has become a focal point in B-SDN cybersecurity, as evidenced by the literature reviewed in the previous sections. The surveyed research indicates that nearly all proposed frameworks incorporate an AI/ML solution as a core component of their methodology. This section explains the specific impact of AI on cybersecurity within this context and presents a brief discussion on the distinct use cases of AI in B-SDN integration.

**Figure 10:**
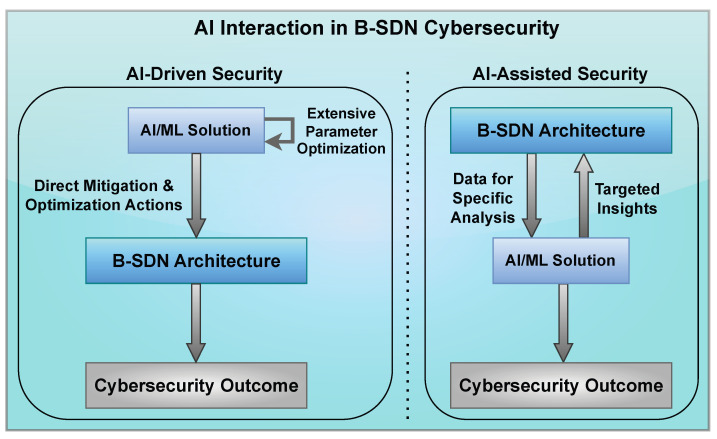
High-level taxonomy of AI interaction in B-SDN cybersecurity, distinguishing AI-driven enforcement mechanisms from AI-assisted analytical frameworks and their respective roles in achieving robust security outcomes.

AI integration in B-SDN cybersecurity can be realized through two complementary paradigms, namely, *AI-driven* and *AI-assisted* security, each differing in the level of autonomy and control over network operations. In the AI-driven approach, AI/ML models are tightly coupled with the B-SDN control plane, enabling direct mitigation actions, dynamic policy enforcement, and continuous optimization of security and network parameters. Conversely, the AI-assisted paradigm primarily leverages AI/ML for targeted analysis and insight generation while the B-SDN architecture provides relevant data to support informed decision-making without direct enforcement. Together, these interaction modes illustrate how AI can operate either within or alongside the B-SDN control loop to enhance cybersecurity outcomes across diverse operational contexts, as illustrated in Figure 10.

For instance, in [239], typical SDN traffic management is offloaded to DL models, specifically CNNs. In this approach, much of the optimization effort focuses on regularization parameters. The system’s cybersecurity performance is evaluated using learning metrics such as F1-scores and precision, with further development conditioned on improving the DL model’s performance. Similarly, in the IIoT sector, ref. [100] centers its discussion around KNN and RSL. The mitigation of various attack types, such as tampering, impersonation, misrouting, or overflow, depends on the performance of these algorithms. Moreover, the authors’ training on the Industrial Control System Cyber Attack Dataset shifts the primary research focus toward optimizing the deployment of these AI solutions. In another example, ref. [118] employs the SMO heuristic algorithm specifically to enhance the AI model, rather than directly addressing a specific B-SDN architectural problem.

Conversely, other papers implement AI as a targeted supporting tool. For example, ref. [98] employs DL specifically to precisely identify attacks and minimize false negatives in DDoS detection. This paper focuses on the detection scheme deployed at the fog and edge computing layers, where SDN serves as a monitor and traffic analyzer, and BC addresses failure issues and attack identification. Another example of the supporting use of AI is found in [142], where AI is primarily used to remove unnecessary data via NLP rather than directly executing cybersecurity defense logic. Further details on these specific implementations can be found in the preceding section.

Despite the significant advantages of integrating AI into B-SDN frameworks, several critical challenges regarding model robustness and lifecycle management must be addressed. Many surveyed studies rely on static, historical datasets (e.g., NSL-KDD), raising concerns about dataset bias and limited model generalization when deployed in diverse, real-world network environments. Furthermore, the centralized nature of SDN controllers and the distributed training environments of Federated Learning expose these AI models to sophisticated adversarial threats. These include adversarial examples designed to evade detection, poisoning attacks aimed at corrupting training data, and inference attacks targeting data privacy. As network environments continuously evolve, deployed models are also highly susceptible to concept drift, requiring dynamic retraining mechanisms to maintain their detection efficacy over time. Finally, the inherent “black-box” nature of complex models like Deep Learning underscores a pressing need for explainability (XAI), ensuring that automated B-SDN security responses remain transparent, auditable, and verifiable by network administrators.

Table 18 provides a comprehensive overview of the AI methodologies reviewed in this section. The table is structured by *Domain* and introduces a classification of *AI Interaction*, distinguishing between fully AI-Driven and AI-Assisted approaches. For each entry, the specific *AI Technique* is listed alongside the corresponding *Ref.*, illustrating the methodological diversity-spanning DL, RL, and hybrid models used to address domain-specific security constraints.

### 11.1. AI-Driven B-SDN Security

The integration of ML and DL into B-SDN architectures significantly enhances security mechanisms. For instance, flow entry verification and traffic categorization in HyperLedger private BFT BCs can be automated using AI to resolve trust measurements. Specifically, DL models using CNNs can verify whether SDN traffic flows are legitimate or malicious, although challenges regarding model overfitting and traffic fluctuations remain [239].

In VANETs, QoS and security can be simultaneously improved through Q-learning-based optimization, thereby enabling energy-efficient bandwidth management. Preventive success against Sybil, Smurf, and DoS attacks is achievable by having ML iteratively select optimal routing paths, maintaining network performance via RL [137].

IIoT environments benefit from similar AI integrations. Forged commands and misrouting attacks targeting industrial systems can be mitigated using KNN and RSL trained on industrial control datasets, utilizing centralized SDN for forwarding and private BC for integrity [100]. Furthermore, Dual Fuzzy Neural Networks (DFNN) enable effective inspection of packet content and headers to detect replay and impersonation attacks while maintaining low latency [42]. Collaborative cyberthreat detection with ensured data privacy can also be established via FL approaches, protecting the aggregation of industrial models [92].

Regarding DDoS defense, a combination of BC sharding and ResNet effectively secures SDN environments while preserving privacy. This approach utilizes distinct datasets for current attack scenarios and legacy traffic to maintain high F1, accuracy, and precision scores [232]. Alternatively, zero-day attack detection is feasible through unsupervised learning that scans network traffic over extended periods to identify anomalies within decentralized Ethereum-based architectures [226].

Collaborative defense mechanisms are further enhanced by FL, allowing SDNs to extract features and train detection models without sharing private data. By utilizing Ethereum smart contracts for integrity, models can be collaboratively built to detect R2L, U2R, and probe attacks with high efficiency [219]. Additionally, the Poaching Raptor heuristic algorithm can tune DNN classifier weights to significantly increase true positive rates in DDoS detection, utilizing Ethereum for efficient access control [225]. For authentication, diverse AI schemes offer robust solutions. GANs for network slicing and hybrid neural decision trees facilitate secure authentication handovers [126]. Vulnerabilities in authentication honeypots can be mitigated using DL-based packet validation and RL at edge servers [49]. In terms of mitigation, ANN-based models can effectively detect attacker nodes and optimize traffic payloads by learning from historical IoT attack data. In this architecture, private BC enhances scalability and fault tolerance, while the distributed SDN functions as a firewall [102].

Smart grid infrastructure also relies on these technologies. Hierarchical SDN routing combined with Ethereum BC ensures integrity, where DL-based intrusion detection using self-attention mechanisms identifies flooding and botnet attacks [119]. Similarly, for smart cities, a hybrid framework integrating Squeeze-Excitation Bidirectional LSTM (Bi-LSTM) models with the Honey Badger Algorithm optimizes traffic control and threat detection [165]. In IoV environments, DRL enables reliable task receiver selection and dynamic adjustment of the consensus algorithm to prevent Sybil and collusion attacks. This hierarchical approach supports dynamic task allocation and resource management [144]. IDS trained via CNNs on Industrial Control Systems datasets can effectively identify injection, scanning, and MiTM attacks in IIoT frameworks [101]. Additionally, anomalous switch requests in industrial networks can be identified by Deep Boltzmann Machine flow analyzers validated through voting-based consensus [146]. Microservice management can be secured using a three-layer AI structure (CNN, probability, and filter) within a Consortium BC to prevent tampering [177]. In the domain of cloud forensics, Graph Neural Network (GNN)-based smart contracts automate evidence collection and track data activities using secure hashing algorithms [178]. Energy-aware task scheduling and offloading in IoT are optimized via learning agents that maximize action-based rewards, ensuring encrypted communication for resource reservation and load balancing [215].

In cyber-physical environments, DDoS detection models can be constructed using Random Forest algorithms, utilizing PCA at the edge to reduce data complexity [224]. RL can also be applied to learn virtual system attributes for application in real-world environments [238]. Specific protocol security, such as BGP hijacking detection and route validation, is enhanced through DRL, which reduces the complexity of security measures [223]. Similarly, malicious UAVs involved in eavesdropping or message alteration can be detected through AI-driven flow analysis combined with secure BC data dissemination [159]. Attack mitigation decisions regarding replay, DDoS, or spoofing can be automated using DL combined with heuristic algorithms such as chaotic radial movement optimization [125]. Furthermore, malicious IoT devices can be identified by analyzing energy characteristics with DL models, utilizing SMO for weight tuning [118]. Scalability in big data and finance sectors is improved by using RL to process routing information and learn from route construction processes, showing high performance against flooding attacks [148]. Energy-efficient routing and trading-based load balancing can be achieved through RL algorithms and Stackelberg Game theory, which dynamically learn the current network state [197]. Cloud–edge attack identification is facilitated by AI pattern detection, while access management relies on a popularity-based democracy scheme to prevent unapproved access [184]. Latency and scalability issues in smart cities can be mitigated using DL-based cloud layers trained on smart energy datasets [146].

Security deployment and resource allocation in mobile edge computing are optimized using a Multi-Agent Deep Deterministic Policy Gradient (MADDPG) algorithm, which dynamically schedules security functions. This approach effectively balances security requirements with latency and energy constraints [152]. Finally, sophisticated cyber threats in Industry 4.0 can be detected with high accuracy using TensorFlow-based CNNs and RNNs within a unified platform, utilizing RL to adapt to evolving attack vectors [227].

### 11.2. AI-Assisted B-SDN Security

While many frameworks position AI as the core driver of security, others use ML as a supporting tool in B-SDN architectures. For example, intrusion detection and malware prevention in Smart Grid settings are effectively managed using FL [24]. To counter eavesdropping in UAV inter-communication, outlier signals can be identified using the K-means clustering algorithm [27]. Although rare in this domain, NLP has been applied to filter unnecessary data shared between vehicles during driving operations [142]. In the context of 6G service virtualization for vehicles, support is provided by FL, deep optimization, and RL [127]. Furthermore, automatic intrusion detection in SDN systems relies heavily on DL dependencies [121]. Similarly, accurate identification of DDoS attacks within fog and edge computing frameworks is achieved through DL implementations [98].

A four-layer architecture that combines authentication and flow-rule verification effectively mitigates attacks in SDN/NFV-enabled clouds. This approach utilizes the Prince algorithm for lightweight authentication and Selective Load Optimization (SLO) to prevent flow table overloading. Furthermore, flow feature classification at the control plane is handled by RNN, while suspicious packets are inspected at the application layer using a Multinomial Naïve Bayes classifier to achieve high precision [133].

The integration of fog and edge computing simplifies and improves bandwidth efficiency in IoT attack detection frameworks. In such systems, SDN monitors traffic, Ethereum-based smart contracts handle mitigation and decentralization, and DL models trained on the NSL-KDD dataset perform the actual attack detection [210].

Security during link failure recovery in SD-IoT environments is enhanced by deploying ANN for immediate DDoS detection and SLO for selecting optimal forwarding switches. This ML-driven approach prevents the exploitation of vulnerabilities during recovery, while the BC layer ensures immutable verification of flow rules [194].

## 12. Implementation and Performance

**Figure 11:**
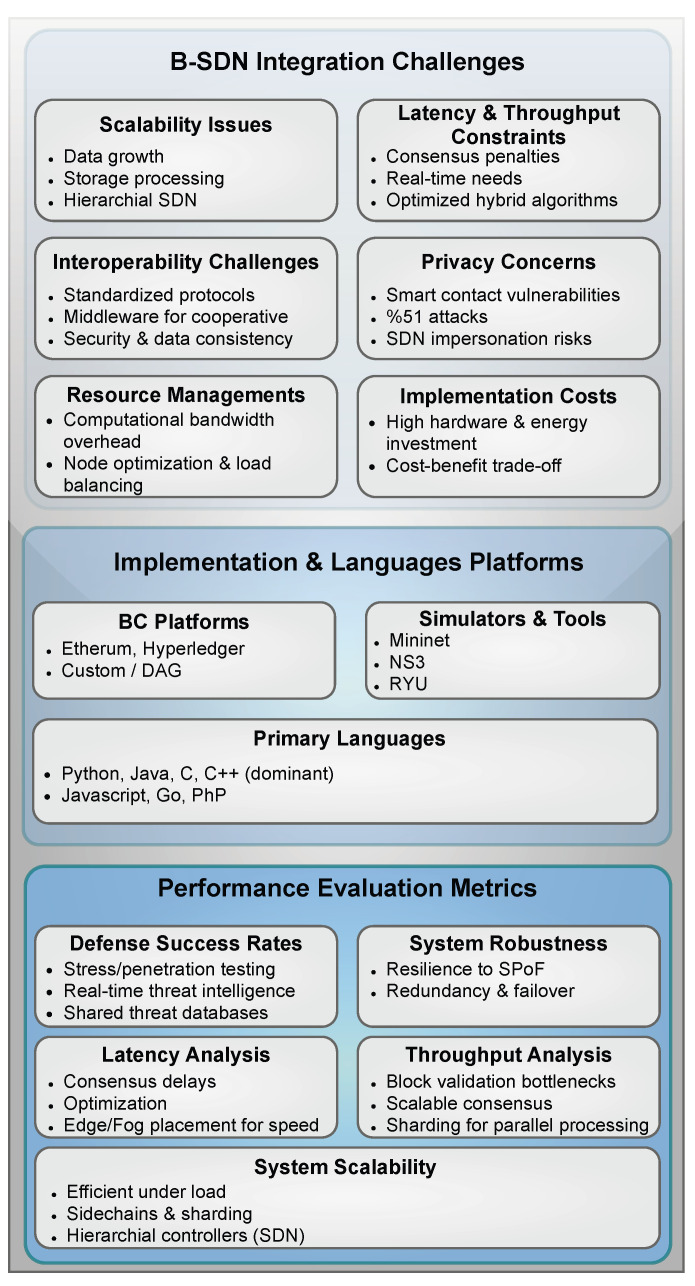
Overview of B-SDN integration challenges, implementation considerations, and performance evaluation metrics.

This section evaluates the cybersecurity performance metrics of B-SDN architectures. While various methodologies exist for system measurement, the networking perspective prioritizes threat elimination efficiency, response time, and resource expenditure. The following subsections analyze these performance metrics, implementation environments, and integration challenges. Figure 11 presents a structured view of the key challenges and considerations involved in integrating B-SDN. The figure highlights significant integration challenges, including scalability limitations, latency and throughput constraints, interoperability issues, privacy concerns, complex resource management, and implementation costs. It further outlines practical implementation aspects, including programming languages, BC platforms, and simulation tools commonly used in B-SDN development. Finally, the figure summarizes performance evaluation metrics—covering defense effectiveness, robustness, latency, throughput, and scalability—that are essential for assessing the efficiency and resilience of B-SDN architectures in real-world deployments.

### 12.1. Performance Evaluation Metrics

Evaluating the effectiveness of B-SDN architectures requires a multi-dimensional performance assessment framework that captures both networking efficiency and security resilience. Unlike conventional SDN environments, B-SDN systems must account for the additional computational and communication overhead introduced by Blockchain-based consensus and ledger synchronization processes. Therefore, performance evaluation extends beyond traditional network metrics and incorporates security-oriented and architectural indicators.

To provide a concrete empirical baseline, Table 19 summarizes an evidence-grounded comparison of representative B-SDN studies across *Ref.*, *Controller*, *BC*, *Consensus*, *Setup*, *Attack*, *Data*, *Delay*, *Throughput*, *Detection*, *Overhead*, and *Limitation*. The *Controller* and *BC* columns show that many works use generic SDN models and heterogeneous ledger designs, while *Consensus* ranges from PoW and M-DPOS to mechanisms that are only discussed or not reported. The *Setup*, *Attack*, and *Data* columns indicate a strong reliance on simulation/emulation, synthetic workloads, and limited public datasets such as NSL-KDD. The *Delay*, *Throughput*, *Detection*, and *Overhead* columns further show uneven metric reporting, with some studies providing numerical values and others requiring N/R or N/A. Finally, *Limitation* captures the main comparability issues, including missing implementation details, simulation-only validation, absent public datasets, and incomplete performance reporting.

The following subsections present the key evaluation dimensions—latency, throughput, robustness, scalability, and defense success rates—that collectively determine the operational viability of B-SDN deployments in real-world and high-demand network scenarios.

#### 12.1.1. Latency Analysis

The integration of B-SDN introduces latency constraints primarily driven by consensus mechanisms required for ledger validation. These delays can impede the real-time responsiveness essential for SDN operations. To mitigate this, optimizing consensus protocols is critical; for instance, modified DPoS has been adopted to balance security with execution speed [200]. Furthermore, strategically placing BC nodes within edge or fog computing layers reduces transmission latency by processing attack-mitigation logic closer to the data source [98]. Controller synchronization must be designed to accommodate these transactional delays to ensure timely policy updates for threat shielding.

#### 12.1.2. Throughput Analysis

Throughput is a critical determinant of the efficiency of attack interception and data processing. The validation latency and propagation delays inherent to block generation can create bottlenecks in high-velocity network environments. Scalable consensus mechanisms are therefore employed to enhance the rate of cyber attack response [200]. Additionally, architectural optimizations such as sharding facilitate parallel transaction processing, significantly improving the system’s capacity to handle high data volumes without degrading SDN performance [114].

#### 12.1.3. System Robustness

Robustness defines the system’s resilience against failures and targeted disruptions. The decentralized architecture of B-SDN inherently mitigates risks associated with SPoF [231]. However, centralizing the SDN controller requires specific protective measures. Redundancy and failover mechanisms, such as two-way switching strategies, are implemented to maintain operational continuity during active cyber threats [28].

#### 12.1.4. System Scalability

Scalability ensures that the B-SDN framework remains efficient as network load increases. Traditional BC platforms such as Bitcoin and Ethereum often present throughput limitations that hinder scalability. To address this, solutions utilizing sidechains and sharding are implemented to distribute the computational load [137]. Concurrently, SDN architectures employ hierarchical or decentralized controller designs to scale dynamically with network demands, ensuring efficient interaction between the ledger and network management planes.

#### 12.1.5. Defense Success Rates

The efficacy of B-SDN integrations is quantified through stress testing, penetration testing, and simulation scenarios. Collaborative frameworks leverage BC to enhance authentication and access control, thereby reducing the risk of identity spoofing. Moreover, integrating real-time threat intelligence enables comprehensive forensic analysis and a deeper understanding of potential vulnerabilities [79]. Continuous updates to shared threat databases enable the system to proactively adapt to emerging vectors, thereby improving overall prevention and mitigation scores.

### 12.2. Implementation Languages and Platforms

A review of the literature indicates a predominance of Python as the primary implementation language, evidenced by its extensive use in pseudo-code and experimental setups. Java and C/C++ are secondary choices, while languages such as JavaScript, Go, and PHP are used less frequently.

Regarding BC platforms, Ethereum is the most widely adopted solution for B-SDN security, followed by Hyperledger. Bitcoin is generally considered unsuitable for real-time cybersecurity implementations due to its scalability constraints, prompting the development of custom BC solutions and DAG-based architectures to meet specific performance requirements. Solidity remains the dominant language for smart contract development, surpassing Serpent by a significant margin.

Simulation environments rely heavily on Mininet, which is used in approximately one-third of the surveyed studies. NS3 and Ryu function as prominent alternatives. Researchers also employ diverse combinations of tools, including Floodlight, OpenFlow, and ONOS, as well as custom simulators developed in MATLAB or Java.

### 12.3. B-SDN Integration Challenges

Integrating B-SDN for cybersecurity introduces distinct technical and operational challenges that require systematic architectural consideration.

#### 12.3.1. Scalability Issues

The exponential growth of data within B-SDN frameworks necessitates robust storage and processing solutions. Techniques such as sharding, sidechains, and off-chain storage are essential to prevent performance bottlenecks on the BC layer. On the SDN side, hierarchical controller deployments are required to manage large-scale networks effectively.

#### 12.3.2. Latency and Throughput Constraints

Consensus mechanisms often impose latency penalties that conflict with the low-latency requirements of real-time threat mitigation. The selection of optimized or hybrid consensus algorithms is requisite to minimize delays and maintain high throughput during active defense operations.

#### 12.3.3. Interoperability Challenges

Establishing standardized communication protocols is vital to the seamless operation of B-SDN components. Developing robust middleware solutions is necessary to facilitate cooperative DDoS security schemes and ensure data consistency across heterogeneous IIoT environments.

#### 12.3.4. Privacy Concerns

While decentralization enhances anonymity, B-SDN architectures remain susceptible to privacy-targeted attacks. Securing smart contracts against vulnerabilities and mitigating risks such as 51% attacks are critical. Furthermore, SDN layers must be hardened against impersonation and unauthorized access to preserve system integrity.

#### 12.3.5. Resource Management Challenges

The combined computational and bandwidth overhead of B-SDN presents a significant management challenge. Efficient resource allocation strategies, including node optimization and dynamic load balancing, are essential to prevent system degradation.

#### 12.3.6. Implementation Costs

The deployment and maintenance of integrated B-SDN systems entail substantial financial investment in hardware and energy. Demonstrating a favorable cost–benefit ratio through enhanced security efficacy is necessary to justify the infrastructure expenditure.

## 13. Lessons Learned

The comprehensive analysis of B-SDN integration for cybersecurity reveals that this convergence offers a formidable defense mechanism against varied cyber threats, particularly through the symbiotic relationship between SDN’s global network visibility and BC’s immutable record-keeping. The survey demonstrates that B-SDN shows strong potential in simulated and constrained prototype environments to mitigate attacks such as DDoS, spoofing, and MitM by decentralizing authentication and ensuring the integrity of flow rules. This integration facilitates rapid, automated threat mitigation while significantly reducing the SPoF inherent in traditional centralized controllers. Furthermore, the proposed application of B-SDN across diverse sectors, from securing resource-constrained IoT devices to managing complex Smart Grid transactions, highlights its versatility as a foundational cybersecurity architecture.

However, the practical deployment of these systems reveals that while B-SDN significantly enhances security postures, it is not a panacea. The synthesis of programmable networking with decentralized ledgers fundamentally alters the threat landscape, yet it introduces distinct operational paradoxes that require careful calibration. The successful utilization of B-SDN relies heavily on balancing the computational overhead of consensus protocols with the low-latency demands of real-time network traffic. Consequently, future architectural designs must navigate these trade-offs to transition from theoretical frameworks to deployment-ready solutions.

**The Latency–Security Trade-off:** A primary lesson derived from the surveyed literature is the inherent tension between the real-time requirements of SDN and the latency induced by BC consensus mechanisms. While the decentralized nature of BC provides robust protection against data tampering and SPoF, traditional consensus algorithms, such as PoW, introduce unacceptable delays for time-sensitive network management tasks, such as flow rule updates and intrusion mitigation. Consequently, the adoption of lightweight consensus mechanisms—specifically, PoA and DPoS—has emerged as a necessary compromise to balance immutable security with the millisecond-level responsiveness required by modern networks.
**Scalability as an Architectural Imperative:** Scalability cannot be treated as a peripheral concern; it must be central to the B-SDN design philosophy. The exponential growth of flow table entries and transaction logs in large-scale IoT and 5G environments rapidly saturates standard BC implementations. Effective frameworks effectively mitigate this by decoupling storage from computation, utilizing off-chain storage solutions (e.g., IPFS) for bulk data while reserving the ledger solely for critical metadata and hashes. Furthermore, the deployment of hierarchical SDN controller architectures is essential for managing distributed load, ensuring that the integration scales linearly with network expansion rather than creating processing bottlenecks.
**The Necessity of AI Automation:** The complexity of managing B-SDN infrastructures exceeds the capacity of static rule-based systems. AI and ML have transitioned from optional enhancements to critical components for anomaly detection and traffic optimization. Specifically, FL has demonstrated exceptional utility in B-SDN environments, enabling distinct network domains to collaboratively train threat-detection models without exposing sensitive local data. This synergy not only preserves privacy but also ensures that defense mechanisms evolve dynamically alongside polymorphic cyber threats.
**Interoperability and Standardization Gaps:** A significant barrier to widespread adoption is the lack of standardized protocols for B-SDN interaction. The current landscape is fragmented, with disparate custom implementations preventing seamless collaboration across multi-domain environments. The development of universal APIs and cross-chain interoperability standards is critical to enabling cohesive defense strategies that span heterogeneous networks, cloud providers, and industrial sectors. Without such standardization, B-SDN security remains isolated within specific silos, limiting its potential for global threat intelligence sharing.
**Resource Constraints in Edge Environments:** Implementing B-SDN cybersecurity in resource-constrained environments, such as IIoT and MEC, demands rigorous resource optimization. The computational overhead of encryption and ledger synchronization can deplete edge device battery life and bandwidth. Successful implementations demonstrate that offloading heavy computational tasks to fog or cloud layers, while retaining lightweight verification functions at the edge, is the optimal strategy. This hierarchical resource management ensures that security protocols do not degrade the operational efficiency of the underlying physical infrastructure.

## 14. Future Research Directions

This section delineates future research prospects across critical sectors, evaluating the probability of positive outcomes and potential setbacks. The discussion highlights select challenges to provide a concise overview of the field.

**Figure 12:**
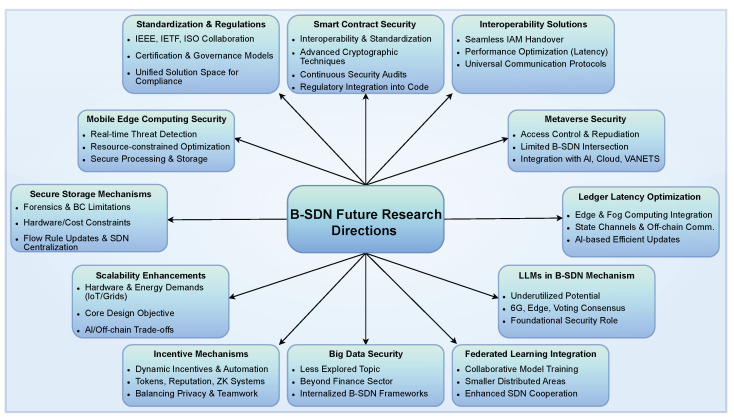
Taxonomy of future research directions for B-SDN.

As illustrated in Figure 12, the future landscape of B-SDN cybersecurity research is multifaceted, spanning architectural optimizations, integration with emerging technologies, and policy standardization. Key architectural challenges include enhancing smart contract security, optimizing ledger latency, and ensuring robust scalability to meet industrial demands. Simultaneously, the convergence with cutting-edge domains such as the Metaverse, Big Data, and MEC presents novel opportunities for decentralized defense. Furthermore, the adoption of advanced AI methodologies such as FL and LLMs, alongside the development of robust incentive mechanisms and global standardization protocols, constitutes the strategic frontier for next-generation B-SDN ecosystems.

1. **Smart Contract Security:** Surveys of the B-SDN literature indicate that a significant portion of research incorporates smart contracts, a trend that inevitably attracts malicious actors. Ensuring the privacy and resilience of these mechanisms requires establishing interoperability and standardization across diverse BC platforms. Future research must prioritize advanced cryptographic techniques and continuous security audits to mitigate emerging vulnerabilities. Furthermore, integrating regulatory requirements directly into smart contract code enables automated compliance and enhances the infrastructure’s security posture.
2. **Interoperability Solutions:** B-SDN cybersecurity systems face significant interoperability hurdles, exacerbating underlying security concerns. Since these issues are inherent to BC, any solution must address the complex web of interconnected dependencies. Identity and access management systems require seamless handover schemes to protect personal information as B-SDN solutions expand into sensitive areas. Future research must prioritize performance optimization to ensure that B-SDN integration operates with minimal latency. As indicated by the surveyed literature, the primary need is the development of robust, universally agreed-upon communication protocols to effectively combat malicious packet activity.
3. **Secure Storage Mechanisms:** Unlike the previously discussed challenges, storage capacity and data management represent critical vulnerabilities, particularly in the context of buffer attacks. Forensics management schemes that rely on BC for communication face inherent storage limitations that complicate the retention of valuable attack data. Increasing hardware requirements and costs further impede the steadiness of cybersecurity responses. Research into optimizing flow rule updates and addressing the centralization of SDN control is vital to managing the storage constraints inherent in the fusion of these technologies. Upgrades addressing the internal mechanics of B-SDN storage are essential for industrial advancement.
4. **Ledger Latency Optimization:** Optimizing ledger updates and latency is crucial to the efficacy of B-SDN cybersecurity solutions. Integrating edge and fog computing principles within B-SDN architectures significantly decreases response times. The deployment of state channels and off-chain communications on SDN offers further latency reductions. AI solutions capable of learning efficient update mechanisms represent a key area for future research, ensuring that B-SDN responses remain competitive with other industrial solutions.
5. **Scalability Enhancements:** Scalability represents a primary challenge, as the hardware and energy demands of B-SDN cybersecurity grow disproportionately compared to traditional methods. Solutions for IoT and Smart Grids often face these constraints. While AI integration offers potential optimizations, it often shifts energy burdens to the training phase, concealing the underlying problem. Similarly, off-chain and edge computing solutions distribute hardware costs across diverse locations but may obscure communication overheads. Future research must centralize scalability as a core design objective rather than treating it as a peripheral concern.
6. **Metaverse Security:** Despite the significant investment interest in the Metaverse, research into its intersection with B-SDN cybersecurity remains limited. Current efforts primarily address access control schemes to securely connect the Metaverse and mitigate repudiation attacks [179]. The technology has not yet been extensively combined with AI, cloud, or VANETs within the B-SDN context. Consequently, the field lacks substantial research value relative to the sunk costs of its cybersecurity implementation.
7. **Big Data Security:** Big Data remains a less-explored topic in B-SDN cybersecurity, despite the direct relevance of security applications to its use cases and infrastructure [148]. Current deployments predominantly focus on the finance sector, leaving a significant research gap regarding the securing of Big Data internalized within B-SDN frameworks across other industrial sectors.
8. **Mobile Edge Computing Security:** The convergence of MEC with B-SDN promises to revolutionize real-time threat detection and response. Although the current literature is sparse, the potential for secure data processing, storage, and maintenance without excessive computational overhead is significant [46,98]. Given the resource-constrained nature of mobile environments, optimizing B-SDN cybersecurity for deployment on mobile infrastructure requires focused investigation.
9. **Federated Learning Integration:** Analysis of the surveyed literature indicates that FL has emerged as a predominant AI approach, offering high reliability for collaborative model training across multiple attack vectors. Research has primarily focused on large-scale industrial settings and grids. Future investigations should explore the application of FL in smaller, distributed operational areas to further decentralize security efforts. Furthermore, enhancing SDN cooperation schemes and network packet dissemination frameworks is essential to optimize the underlying infrastructure for FL-based cybersecurity.
10. **Incentive Mechanisms:** The development of robust, deployable incentive mechanisms is essential to fostering collaboration in B-SDN cybersecurity. Dynamic incentives coupled with novel tools facilitate adaptive cooperation among network participants [200]. Research explores automation through transparent token distribution, reputation algorithms, and zero-knowledge systems to enhance trust and participation [44,183,184]. Leveraging SDN virtualization and flexibility to support these mechanisms presents a promising avenue for future work. The primary challenge lies in balancing privacy-preserving techniques with the need to encourage effective teamwork within the network.
11. **LLMs in B-SDN Security:** LLMs are currently underutilized in B-SDN security, with limited applications found in 6G technologies, edge nodes, and voting consensus for VANETs data sharing [142]. The potential of LLMs to enhance cybersecurity solutions in B-SDN architectures warrants increased attention. Similar to other emerging approaches, LLMs offer a broad range of options to play a foundational role in future security frameworks.
12. **Standardization and Regulations:** Standardization and regulatory frameworks are pivotal for the widespread adoption of B-SDN cybersecurity. Organizations such as IEEE, IETF, and ISO must collaborate to establish robust security standards. Continued support for certification and governance models involves coordination between industry stakeholders and regulatory bodies. Achieving a unified solution space for standardization is critical to resolving the compliance and regulatory challenges that currently hinder the advancement of B-SDN security solutions.

## 15. Conclusions

Traditional centralized security and standalone SDN implementations are increasingly vulnerable to threats such as SPoF, DDoS, MiTM, and insider attacks. The integration of B-SDN addresses these flaws by coupling SDN’s programmable visibility with BC’s decentralized trust. This convergence is a critical response to the expanding digital market, providing a necessary defense framework against an array of sniffing, spoofing, and malicious packet attacks where infrastructure integrity is important.

Our findings show the applicability of B-SDN across diverse sectors. In IoT/IIoT, it shows improvement in securing devices against tampering, though energy limitations persist. For 5G/6G, it enhances handover and slicing security but faces latency constraints from consensus overhead. VANETs and IoV benefit from improved trust management despite the challenges posed by high mobility. Smart Grids gain secure P2P trading and data integrity, while cloud/edge environments leverage decentralized storage and robust access control. The framework further ensures resilient links in UAV networks, enforces granular access to electronic health records in Healthcare, and secures public records in Government applications. Additionally, B-SDN mitigates DDoS in CDNs, protects smart city ecosystems, and improves core routing integrity for BGP/DNS.

However, critical implications and limitations remain. The latency–security trade-off is a primary challenge; heavy consensus mechanisms such as PoW conflict with real-time SDN requirements, necessitating lightweight alternatives such as PoA or DPoS. Storage strain is another concern, as growing ledgers tax controller nodes requiring off-chain solutions. Energy efficiency also conflicts with the limited battery life of edge devices, while BC’s inherent transparency often clashes with privacy regulations such as GDPR. Furthermore, dual-architecture complexity increases administrative burdens, and a lack of unified protocols hinders interoperability. Finally, incorporating AI, specifically FL, is essential to automate detection and optimize resources within these complex systems.

In conclusion, B-SDN represents a transformative step toward resilient, self-defending infrastructures. Future research must optimize the cost–benefit ratio by standardizing and automating with AI. We affirm that the combined capabilities of B-SDN will be pivotal in securing interconnected global networks, transitioning security from reactive response to proactive, immutable defense.

## Figures and Tables

**Table 1:** Quantitative distribution of the 194 core B-SDN cybersecurity studies categorized by domain, platform, consensus mechanism, simulation tools, targeted attacks, defense strategies, and evaluation metrics.

Category	Attribute	Study Count
**Domain/Use Case**	IoT	93
	Cloud/Fog/Edge	70
	Network Technologies	44
	Smart Cities	25
	VANETs/IoV	20
**Blockchain Platform**	Ethereum	65
	Hyperledger	36
	Custom	34
	MultiChain	9
**Consensus Mechanism**	PoW	32
	PBFT	24
	PoS	19
	Custom	15
	PoA	12
**SDN Controller**	OpenDaylight	24
	Ryu	19
	ONOS	13
	Floodlight	11
	POX	7
**Simulator**	Mininet	72
	NS-3	18
	MATLAB	5
	OMNeT++	4
**Attack Category**	DDoS/DoS	99
	Spoofing	61
	MiTM	40
	Privilege Escalation/Unauthorized Access	21
	Insider Threats/Collusion	21
**Defense Strategy**	Prevention	123
	Detection	107
	Mitigation	73
**Evaluation Metric**	Throughput/Bandwidth	107
	Detection Accuracy/Success Rate	85
	Latency/Processing Time	81
	Resource Utilization (CPU/Memory)	23

**Table 2:** Classification of representative B-SDN cybersecurity studies by their empirical maturity, distinguishing between theoretical frameworks, simulations, prototypes, and real-world deployments.

Conceptual Level	References
**Conceptual Frameworks **	[24,25,26,27,28,29,30,31,32,33]
**Simulation-based Studies**	[34,35,36,37,38,39,40,41,42,43]
**Prototype Implementations**	[44,45,46,47,48,49,50,51,52,53]
**Real-world Deployments**	[54,55,56,57]

**Table 3:** Comparison of widely used BC platforms in terms of participation model, consensus mechanism, implementation language, and smart contract support.

BC Platform	Participation Model	Consensus Mechanism	BC Language	Smart Contract
Bitcoin	Public	PoW	C++	No
Hyperledger	Private	Pluggable (Raft, PBFT, PoA)	Go, Java, Node.js	Yes
Ethereum	Public	PoS/PoA	Go, Rust, C++	Yes
Multichain	Private	Round Robin/PBFT	C++	Limited
Ripple	Consortium	RPCA/FBA	C++	No
IOTA	Public	DAG (FPC)	Rust, Go	Yes

**Table 4:** Comparative overview of prior survey studies on the B-SDN, categorized by application domains, focus areas, and addressed research perspectives. (Dimensions: Ind. Scope = Industry Scope; Att. Cov. = Attack Coverage; Def. Tax. = Defense Taxonomy; AI Int. = AI Integration; Perf. Analysis = Performance Analysis; Impl. Cons. = Implementation Considerations; Sec. Mech. = Security Mechanisms). Green color + check mark and red color + cross mark are used for inclusion and exclusion of dimensions in the studies, respectively.

Ref.	Year	Category	Ind. Scope	Att. Cov.	Def. Tax.	AI Int.	Perf. Analysis	Impl. Cons.	Sec. Mech.
[12]	2020	General Integration	×	×	×	×	×	✓	✓
[10]	2021		×	×	×	×	×	×	✓
[9]	2022		×	×	×	✓	×	×	✓
[11]	2023		×	×	×	×	×	×	×
[22]	2024		×	×	✓	×	×	×	✓
[14]	2019	IoT & Virtualization	✓	×	×	×	×	×	×
[4]	2021		✓	×	×	×	×	×	✓
[18]	2022		✓	×	×	×	×	×	×
[3]	2023		✓	×	×	×	×	×	×
[16]	2023		✓	×	×	×	×	×	×
[13]	2024		✓	×	×	×	×	×	✓
[19]	2020	Critical Infrastructure	✓	×	×	×	×	×	×
[6]	2023		✓	×	×	×	×	×	✓
[5]	2023		✓	×	×	×	×	×	✓
[8]	2023		✓	×	×	×	×	×	×
[7]	2024		✓	×	×	×	×	×	×
[21]	2022	Threat-Centric Approaches	×	✓	×	×	×	×	✓
[20]	2023		✓	✓	×	×	×	×	✓
[17]	2023		✓	✓	×	×	×	×	✓
**This survey**	**2026**	**Industry-Threat Unified**	✓	✓	✓	✓	✓	✓	✓

**Table 5:** Selected representative real-world large-scale cyber incidents reported in recent years, highlighting attack types, affected targets, duration, and resulting operational and economic damages.

Ref.	Year	Attack Name	Attack Type	Targets	Duration	Damage
[80]	2016	Mirai Botnet	Botnet, DDoS	Dyn	-	IP cameras and routers mainly affected, which then started DDoS attacks on sites such as CNN media and Netflix.
[81]	2022	AcidRain	Access Violation	Viasat Satellite internet	1 week	To permanently disable routers at satellite internet service caused the halt of wind turbines in Germany.
[82]	2024	WazirX cyber attack	Malicious Packet Attack	WazirX, Liminal	-	USD 230 million worth of asset was stolen; where WazirX had to platform shut down withdrawals.
[83]	2022	BNB Chain	Tampering, Malicious data	Cosmos, BSC Token Hub	1 day	USD 570 million worth of damage inflicted where Binance suspended deposits and withdrawals.
[84]	2022	Nomad Bridge Hack	Replay Attack	Nomad Crosschain	1 day	USD 200 million stolen because of error at the project’s main smart contract services.
[85]	2024	JAVS Cyber Attack	Malware	US Legal System	1 month	More than 10,000 US courtrooms, jails, and prisons affected with malware at JAVS network.
[86]	2024	Mercku cyber attack	Phishing	Canadian, European ISP’s	-	URL shortener abused by the attacker, attacks have been thwarted.
[87]	2024	Squarespace cyber attack	DNS Hijack, MiTM	Celer Network, Compound Finance	-	Attackers redirect visitors to phishing sites hosting wallet drainers which use Squarespace.

**Table 6:** Research questions guiding the analysis of B-SDN for cybersecurity, including discussion focus, and targeted impact areas.

Research Questions (RQ)	Discussion	Impact/Aim
**RQ1: How do the architectural patterns of B-SDN integrations enhance cybersecurity across various industries and domain-specific use cases?**	BC and SDN leverage mutual strengths to address unique security challenges and architectural patterns across different sectors.	Targets readers in various industries interested in architectural patterns and novel technologies that affect revenue and security.
**RQ2: What are the primary security defense solutions and empirical evaluation practices proposed within B-SDN frameworks?**	This fusion aims to surpass current defense standards regarding scalability, privacy, and flexibility, validated through empirical evaluation.	Targets the academic community to identify research gaps, empirical evaluation practices, and new complex integration problems.
**RQ3: How can attack–defense mapping in B-SDN frameworks effectively mitigate and prevent different cyber attack types?**	The integration offers theoretical and prototype-backed solutions and attack–defense mapping to mitigate and potentially prevent a vast array of specific cyber attacks.	Provides a comprehensive attack–defense categorization and mapping of attacks and defense strategies for industry and academia.
**RQ4: In what ways does AI-assisted security integrate with and optimize cybersecurity in the B-SDN frameworks?**	AI-assisted security automates incident response and optimizes security frameworks within the B-SDN ecosystem.	Analyzes how AI-assisted security enhances security characteristics and offers insights into novel technologies.

**Table 7:** A taxonomy of industry-specific B-SDN defense mechanisms, categorizing existing studies across IoT, 5G/6G, and IoV domains according to the targeted attack types and representative references.

Industry	Attack	Ref.
IoT	DoS/DDoS	[56,57,90,91,92,93,94,95,96,97,98,99,100,101,102,103,104,105,106,107,108,109,110,111,112,113]
	Spoofing	[37,42,57,105,114]
	MiTM	[42,92,101,115]
	SPoF	[91,103,114,116]
	Password	[42,100]
	Replay	[42]
	Impersonation	[42,117]
	Malicious Packet Attacks	[51,56,101,118,119]
	Flooding	[93,94,98,107,110,112,119,120]
	Botnet	[90,119]
	Scanning	[119]
	Flow Table Manipulation	[53,96]
	Topology Attack	[96]
	Miscellaneous	[37,57,79,100,101,102,113,115,121,122,123,124]
5G/6G	DoS/DDoS	[30,32,97,125,126]
	Spoofing	[125,126,127,128,129]
	Replay	[126,127]
	MiTM	[30,127,129]
	SPoF	[130]
	Malicious Packet Attacks	[129,130]
	Miscellaneous	[30,41,127,128,131,132,133,134]
IoV	DoS/DDoS	[135,136,137,138,139]
	Spoofing	[127,128,135,137,139,140,141]
	MiTM	[38,136,140,142,143]
	Sniffing	[127,128,135]
	SPoF	[141,142]
	Sybil Attack	[137,139,143,144]
	Replay Attack	[136,143]
	Impersonation	[136,143]
	Flooding	[138,139]
	Botnet	[143]
	Miscellaneous	[127,128,137,139,143,144,145]

**Table 8:** Industry-wise classification of major security attacks addressed in B-SDN systems across cloud computing, Smart Grid, and UAV domains, along with representative studies from the literature.

Industry	Attack	Ref.
Cloud	DDoS/DoS	[149,150,151,152,153]
	MiTM	[38,42,127,142]
	Spoofing	[42,44,127]
	Tampering	[150,152]
	Flooding	[149,151]
	Impersonation	[42,127]
	Replay	[42,127]
	Miscellaneous	[42,48,127,142,147,149,150,152,153]
Smart Grid	DDoS/DoS	[24,136,154,155,156]
	MiTM	[24,136,140,154]
	Spoofing	[31,39,140,154]
	Malware	[24,119,156]
	Sniffing	[24,31]
	Privilege Escalation	[24]
	Scanning	[119,154]
	Flooding	[119,157]
	Miscellaneous	[119,136,156]
UAV	DoS/DDoS	[27,158]
	MiTM	[27,159]
	Spoofing	[27,37]
	Sniffing	[27,159]
	UAV Hijacking	[27,36,159]
	Miscellaneous	[37,158,159]

**Table 9:** Overview of major security attacks and threat categories identified in B-SDN architectures, categorized by industry domain (healthcare, government, and CDN), along with representative studies.

Industry	Attack	Ref.
Healthcare	DoS/DDoS	[47,160]
	Spoofing	[47]
	Sniffing	[35]
	Replay	[47]
	Tampering	[47,160,161]
	Miscellaneous	[47,160,161]
Government	Spoofing	[162]
	Impersonation	[33]
	Replay	[162]
	Miscellaneous	[33,162]
CDN	DoS/DDoS	[25]
	Spoofing	[25]
	SPoF	[130]
	Miscellaneous	[25,130]

**Table 10:** Categorization of application domains employing B-SDN, mapping Smart City, Network, and Miscellaneous use cases to the corresponding security attack types addressed in the literature.

Industry	Attack	Ref.
Smart City	DoS/DDoS	[91,94,95,164,165,166]
	Spoofing	[164]
	Flooding	[94]
	SPoF	[91]
	Miscellaneous	[36,122,146,165,166]
Network	Tampering	[167,168]
	Insider Threats	[55,169,170]
	Spoofing	[50,171]
	MiTM	[50,171]
	Sniffing	[50,171]
	Unauthorized Access	[168,172]
	Miscellaneous	[50,170,172,173]
Miscellaneous	DoS/DDoS	[146,174,175]
	Spoofing	[26,129,148,176]
	MiTM	[129,177]
	Flooding Attacks	[148,174,175]
	Tampering	[177,178]
	Miscellaneous	[46,129,146,178,179]

**Table 11:** Mapping of major security attacks to defense mechanisms in access control, authentication/verification, and routing solutions, with representative B-SDN studies.

Industry	Attack	Ref.
Access Control	DoS/DDoS	[27,29,57,180,181,182,183,184,185]
	Spoofing	[27,29,57,180,181,182,186]
	MiTM	[27,29,180,183]
	Unauthorized Access	[57,168,172,187]
	Replay	[29,180,183]
	Privilege	[180,181,183]
	Tampering	[168,183]
	Impersonation	[180,184]
	Malicious Packet	[172,182,188]
	SPoF	[29,180]
	Miscellaneous	[27,53,181,188]
Authentication/Verification	Spoofing	[26,42,49,125,126,162,189,190]
	DoS/DDoS	[49,125,126,189,190]
	Replay	[42,49,125,126,162]
	MiTM	[42,49]
	Impersonation	[42,191]
	Sybil	[126,132]
	Tampering	[132,162,192]
	Unauthorized Access	[132,192]
	Miscellaneous	[42,188,190,191]
Routing Solutions	DoS/DDoS	[34,102,193,194]
	Link Failure	[193,194]
	Spoofing	[40,195]
	Tampering	[195,196]
	Miscellaneous	[34,40,102,118,196,197]

**Table 12:** Defense-oriented taxonomy of B-SDN studies, categorizing privacy preservation, secure data sharing, and consensus mechanisms according to the types of attacks they address, with representative references from the literature.

Industry	Attack	Ref.
Privacy	Spoofing	[26,128,195,200]
	Tampering	[128,195,200]
	DoS/DDoS	[136,200]
	MiTM	[136,200]
	Impersonation	[136,200]
	Replay	[136,200]
	Miscellaneous	[48,128,157,199,200]
Data Sharing	DoS/DDoS	[201,202,203,204]
	MiTM	[142,201]
	SPoF	[142,202]
	Miscellaneous	[142,201,204]
Consensus Mechanisms	DoS/DDoS	[28,32,97,112,200]
	MiTM	[143,200]
	Impersonation	[143,200]
	Replay	[143,200]
	Tampering	[143,200]
	Insider Threats	[170,200]
	Miscellaneous	[28,112,143,170,200,205]

**Table 13:** Representative studies employing B-SDN mechanisms to mitigate different classes of attacks across QoS and traffic engineering, scalability and energy efficiency, and storage solutions.

Industry	Attack	Ref.
QoS & Traffic Engineering	DoS/DDoS	[24,25,137]
	Spoofing	[25,40,137]
	Sybil	[40,137]
	MiTM	[24,38]
	Tampering	[25,145]
	Miscellaneous	[24,25,40,137]
Scalability and Energy Solutions	DoS/DDoS	[24,94,95,206]
	Flooding	[94,206]
	Impersonation	[197]
	Miscellaneous	[24,123,188,207]
Storage Solutions	DoS/DDoS	[24,45,185,201]
	MiTM	[24,201,208]
	Tampering	[45,208,209]
	Spoofing	[45,201]
	Impersonation	[117]
	Miscellaneous	[24,209]

**Table 14:** Major security threats and corresponding B-SDN defense mechanisms applied to load balancing, cloud/fog/edge, and logging/monitoring/measurement architectures.

Industry	Attack	Ref.
Load Balancing	DoS/DDoS	[43,49,95,126,155,185]
	Spoofing	[26,49,126]
	Replay	[49,126]
	MiTM	[49]
	Impersonation	[197]
	Sybil	[126]
Cloud/Fog/Edge Technologies	DoS/DDoS	[98,184,206,210]
	Flooding	[98,206,210]
	Spoofing	[211]
	Impersonation	[184]
	Miscellaneous	[52,147]
Logging/Monitoring/Measurement	DoS/DDoS	[24,212]
	Forensics	[79,213]
	Tampering	[103,214]
	SPoF	[103,212]
	Miscellaneous	[24,103,212,214]

**Table 15:** Security attacks addressed by encryption techniques and smart contract–based mechanisms in B-SDN systems.

Industry	Attack	Ref.
Encryption Techniques	MiTM	[143,183]
	Impersonation	[143,197]
	Replay	[143,183]
	Tampering	[143,183]
	DoS/DDoS	[135,183]
	Spoofing	[135]
	Miscellaneous	[52,135,143,183,216]
Smart Contracts	DoS/DDoS	[30,104,136,155,190,193,198,217,218,219,220,221]
	Spoofing	[186,190,198,220]
	MiTM	[30,136]
	Flooding	[30,218]
	Insider Threats	[169,190,198]
	Unauthorized Access	[187,217,222]
	Impersonation	[136,191]
	Replay	[136]
	Scanning	[219]
	Miscellaneous	[52,191,193,221,222,223]

**Table 16:** Comprehensive comparison of security threats, attack types, and corresponding detection, prevention, and mitigation mechanisms in B-SDN, highlighting the supported security properties, SDN functionalities, validation maturity (Conceptual Framework (Theor.), Simulation (Sim.), or Prototype (Proto.)), and additional system-level aspects addressed in the literature.

Ref.	Threat	Security	Validation	Energy Efficient	Decentralization	Immutability	Traceability	Transparency	Trust	Anonymity	Privacy	Flow Rule Update	Forwarding	Automation	Virtualization	Monitoring	Decoupling	Dynamic Management	Other Aspects
[24]	DDoS	Detection	Theor.	X	X						X	X	X	X			X		Wide Architecture
[155]			Sim.							X			X				X		Bandwidth
[99]					X	X										X	X		Early Detection
[224]					X	X	X							X		X	X		Performance
[203]		Detection Mitigation	Sim.			X	X						X			X	X	X	Performance
[98]					X				X								X		Response Time
[102]				X	X	X	X	X				X						X	Performance
[111]					X	X		X				X	X	X		X			Double-Layered, ML
[43]		Prevention	Sim.			X			X		X							X	Performance
[225]			Theor.		X			X	X									X	Performance
[220]		Mitigation	Sim.		X				X							X		X	Collaborative
[89]	DDoS Botnet	Mitigation	Sim.		X	X		X	X					X			X		Performance
[90]					X														Collaborative
[223]	BGP Attacks	Detection Prevention	Sim.	X	X								X			X		X	Flexible
[92]	DDoS, Botnet MiTM	Detection	Sim.		X	X	X	X		X			X			X		X	Collaborative
[49]	DDoS, MiTM Spoofing	Detection	Sim.	X		X											X		Honeypot
[219]	DDoS, Scanning	Mitigation	Sim.		X	X		X	X										Collaborative
[56]	DDoS Spoofing	Detection Mitigation	Proto.		X	X			X				X			X			Traffic Filtration
[120]	DoS, Intrusion	Detection	Sim.	X	X				X							X			Delay, Energy
[97]	DoS, Malicious Packets	Mitigation Prevention	Sim.	X	X								X					X	Response Time
[101]	Injection, MiTM Intrusion	Detection	Sim.		X	X	X	X					X					X	Performance
[55]	Intrusion	Detection	Proto.		X	X			X					X	X			X	Collaborative
[100]	MiTM, Spoofing Intrusion, Tampering	Detection Prevention	Sim.			X	X					X	X						Performance
[159]	Scanning, MiTM Injection, Malware	Detection Prevention	Sim.						X	X						X		X	Performance
[226]	Zero Day	Detection	Theor.		X														Performance

**Table 17:** Industry-wise categorization of consolidated B-SDN solutions, mapping key design goals (e.g., security, performance, scalability, privacy, resilience, and integrity) to representative studies across multiple domains, including networking, IoT, smart cities, 5G/6G, vehicular networks, IoE/Smart Grids, and UAV systems.

Industry	Goals	Ref.
Networking	Security	[25,29,41,50,89,99,160,177,180,190,193,196,198,200,203,211,212,217,220,225,228,229,230,231]
	Performance	[25,28,43,44,89,169,180,182,184,186,190,198,200,201,212,218,226,231,232]
	Speed	[182,184,217,225]
	Resilience	[193]
	Privacy	[52,201]
	Scalability	[233,234]
	Trust	[228,233]
	Lightweight	[41,50]
	Collaboration	[228]
	Flexible	[223]
	Low Complexity	[223]
	Latency	[234]
	Consistency	[171]
	Integrity	[196]
IoT	Security	[34,53,90,96,98,100,101,147,150,185,202,224,235]
	Speed	[90,104,191]
	Performance	[101,104,164,202,236]
	Privacy	[53,92,191]
	Scalability	[51,116,206]
	Robustness	[53,236]
	Reliability	[92]
Smart Cities	Security	[93,94,95]
	Scalability	[91]
5G/6G	Security	[30,42,49,126,129,130]
	Lightweight	[42,49]
	Privacy	[30,130]
	Scalability	[126]
	Performance	[125,130]
	Speed	[129]
	Resilience	[129]
Vehicular	Privacy	[135,144]
	Security	[135,137,143,188]
	Speed	[137]
	Lightweight	[143]
IoE/Smart Grid	Full Scale	[24]
	Privacy	[31,157]
	Performance	[154]
	Scalability	[31,157]
UAVs	Security	[159]
	Performance	[159]
	Privacy	[159]
	Integrity	[159]

**Table 18:** Domain-wise summary of AI techniques adopted in B-SDN cybersecurity solutions, classifying approaches as either AI-Driven or AI-Assisted.

Ref.	Domain	AI Interaction	AI Technique
[239]	General	AI-Driven	DL (CNN)
[42]			DFNN
[232]			ResNet
[226]			Unsupervised Learning
[219]			FL
[225]			DNN + Poaching Raptor
[126]			GAN, Hybrid Neural DT
[125]			DL + Heuristics
[197]			RL + Stackelberg Game
[102]	IoT	AI-Driven	ANN
[215]			Learning Agents
[118]			DL + SMO
[210]		AI-Assisted	DL
[100]	IIoT	AI-Driven	KNN, RSL
[92]			FL
[101]			CNN
[137]	VANET	AI-Driven	RL (Q-learning)
[142]		AI-Assisted	NLP
[127]			FL, Deep Opt., RL
[224]	Cyber-Physical	AI-Driven	Random Forest + PCA
[238]			RL
[119]	Smart Grid	AI-Driven	DL (Self-Attention)
[24]		AI-Assisted	FL
[159]	UAV	AI-Driven	AI-driven Flow Analysis
[27]		AI-Assisted	K-means Clustering
[165]	Smart City	AI-Driven	Bi-LSTM + Honey Badger
[49]	Edge	AI-Driven	DL, RL
[152]	Mobile Edge	AI-Driven	MADDPG (DRL)
[98]	Fog/Edge	AI-Assisted	DL
[121]	SDN	AI-Assisted	DL
[144]	IoV	AI-Driven	DRL
[223]	BGP	AI-Driven	DRL
[148]	Finance	AI-Driven	RL
[184]	Cloud-Edge	AI-Driven	AI Pattern Detection
[178]	Cloud	AI-Driven	GNN
[133]	SDN/Cloud	AI-Assisted	RNN, Naïve Bayes, Sea Lion
[194]	SD-IoT	AI-Assisted	ANN + Sea Lion Opt.
[146]	Industrial	AI-Driven	Deep Boltzmann Machine
[227]	Industry 4.0	AI-Driven	CNN, RNN, RL
[177]	Microservices	AI-Driven	CNN + Probabilistic Filter

**Table 19:** Quantitative performance comparison of representative B-SDN implementations. N/R: not reported; N/A: not applicable.

Ref.	Controller	BC	Consensus	Setup	Attack	Data	Delay	Throughput	Detection Ratio	Overhead	Limitation
[98]	Generic SDN/OVS	Ethereum private	N/R	Mininet	DDoS, ICMP TCP flood	NSL-KDD + synth.	5–12 ms Identification; 5–6 s Recovery	N/R	≈94–95%	N/R	No named controller; implemented consensus not specified; limited attack set.
[200]	Generic	Ethereum	M-DPOS	OMNeT++ INET	Spoofing, DoS/DDoS, replay	N/A	0.33–0.70 ns	18–42 kbps	N/A	0–70 msgs	Simulation only; no IDS dataset or detection-rate evaluation.
[114]	Ctrl. groups	Main/sub chains	PBFT discussed	Conceptual	Controller compromise, SPoF	N/A	N/R	N/R	N/R	N/R	Architecture-only study; no quantitative performance evaluation.
[231]	Generic	BloC-VNE	N/R	Custom simulator	Single-node failure	Synth.	N/A	N/A	N/A	CPU: 0.20–0.34 BW: 0.22–0.39	Evaluates VNE fault tolerance, not IDS or network QoS.
[28]	Multi-ctrl. concept	Private + alliance	N/R	Conceptual	Flow-rule injection, DoS, SPoF	N/A	N/R	N/R	N/R	N/R	No implementation, testbed, or numerical results.
[137]	Generic	Sidechain	PoW + Q-learning	VANET sim.	Sybil, DDoS, Smurf	Synth.	0.34–1.62 ms avg. 0.89 ms	444.5–1586.3 kbps	100% claimed	Energy ≈ 9.6 mJ	Detection claim lacks public dataset and confusion-matrix validation.
[79]	Generic 4 ctrl.	Bitcoin- based	PoW + LHS	NS3	Malicious packets, invalid ports	Synth.	0.2–1.0 ms; resp. ≈ 5.1 ms	50–130 bytes	97–99%; avg. ≈ 98%	10.3–12.0 s processing	Small synthetic setup; throughput reported in bytes, not rate.

## Data Availability

Data are contained within the article.

## References

[1] Saha S. (2026). Blockchain Technology Market Outlook (2026 to 2036). https://www.futuremarketinsights.com/reports/blockchain-technology-market.

[2] Saha S. (2025). Software Defined Networking Market Outlook (2025 to 2035). https://www.futuremarketinsights.com/reports/software-defined-networking-market.

[3] Turner S.W., Karakus M., Guler E., Uludag S. (2023). A Promising Integration of SDN and Blockchain for IoT Networks: A Survey. IEEE Access.

[4] Alhawamdeh M., Tahboub R. (2021). Enabling Security as a Service for IoT Emerging Technologies: A Survey. Proceedings of the 7th Annual International Conference on Arab Women in Computing in Conjunction with the 2nd Forum of Women in Research.

[5] Amari H., Houda Z.A.E., Khoukhi L., Belguith L.H. (2023). Trust Management in Vehicular Ad-Hoc Networks: Extensive Survey. IEEE Access.

[6] Varma I.M., Kumar N. (2023). A comprehensive survey on SDN and blockchain-based secure vehicular networks. Veh. Commun..

[7] Hayyolalam V., Zekiye A., Abuzahra H., Özkasap Ö., Karakus M., Guler E., Uludag S. (2024). Synergistic Integration of Blockchain and Software-Defined Networking in the Internet of Energy Systems. Proceedings of the 2024 6th International Conference on Blockchain Computing and Applications (BCCA).

[8] Bodkhe U., Tanwar S. (2023). Network management schemes for IoT environment towards 6G: A comprehensive review. Microprocess. Microsyst..

[9] Rahman A., Montieri A., Kundu D., Karim M., Islam M., Umme S., Nascita A., Pescapé A. (2022). On the Integration of Blockchain and SDN: Overview, Applications, and Future Perspectives. J. Netw. Syst. Manag..

[10] Nam Nguyen H., Anh Tran H., Fowler S., Souihi S. (2021). A survey of Blockchain technologies applied to software-defined networking: Research challenges and solutions. IET Wirel. Sens. Syst..

[11] Faruk M.J.H., Cheng J.Q. Integration of Blockchain in Computer Networking: Overview, Applications, and Future Perspectives for Software-defined Networking (SDN), Network Security, and Protocols. Proceedings of the 2023 Tenth International Conference on Software Defined Systems (SDS).

[12] Alharbi T. (2020). Deployment of Blockchain Technology in Software Defined Networks: A Survey. IEEE Access.

[13] Indrason N., Saha G. (2024). Exploring Blockchain-driven security in SDN-based IoT networks. J. Netw. Comput. Appl..

[14] Pohrmen F.H., Das R.K., Saha G. (2019). Blockchain-based security aspects in heterogeneous Internet-of-Things networks: A survey. Trans. Emerg. Telecommun. Technol..

[15] Alam I., Sharif K., Li F., Latif Z., Karim M.M., Biswas S., Nour B., Wang Y. (2020). A Survey of Network Virtualization Techniques for Internet of Things Using SDN and NFV. ACM Comput. Surv..

[16] Rahman A., Islam J., Kundu D., Karim R., Rahman Z., Band S.S., Sookhak M., Tiwari P., Kumar N. (2023). Impacts of blockchain in software-defined Internet of Things ecosystem with Network Function Virtualization for smart applications: Present perspectives and future directions. Int. J. Commun. Syst..

[17] Saha V., Anand G., Ghosh M., Singhal S. Analysis of Blockchain-Based Techniques for the Mitigation of DDoS Attacks in IoT Devices. Proceedings of the 2023 14th International Conference on Computing Communication and Networking Technologies (ICCCNT).

[18] Douha N.Y.R., Bhuyan M., Kashihara S., Fall D., Taenaka Y., Kadobayashi Y. (2022). A survey on blockchain, SDN and NFV for the smart-home security. Internet Things.

[19] Kumari A., Gupta R., Tanwar S., Kumar N. (2020). A taxonomy of blockchain-enabled softwarization for secure UAV network. Comput. Commun..

[20] Chaganti R., Bhushan B., Ravi V. (2023). A survey on Blockchain solutions in DDoS attacks mitigation: Techniques, open challenges and future directions. Comput. Commun..

[21] Muzafar S., Jhanjhi N., Khan N.A., Ashfaq F. DDoS Attack Detection Approaches in on Software Defined Network. Proceedings of the 2022 14th International Conference on Mathematics, Actuarial Science, Computer Science and Statistics (MACS).

[22] Dudukcu D., Karakus M. (2024). Security Dynamics of Blockchain-Enabled SDN Systems: A Taxonomic Approach. Proceedings of the 2024 9th International Conference on Computer Science and Engineering (UBMK).

[23] Page M.J., McKenzie J.E., Bossuyt P.M., Boutron I., Hoffmann T.C., Mulrow C.D., Shamseer L., Tetzlaff J.M., Akl E.A., Brennan S.E. (2021). The PRISMA 2020 statement: An updated guideline for reporting systematic reviews. BMJ.

[24] Radoglou-Grammatikis P., Liatifis A., Dalamagkas C., Lekidis A., Voulgaridis K., Lagkas T., Fotos N., Menesidou S.A., Krousarlis T., Alcazar P.R. (2023). ELECTRON: An Architectural Framework for Securing the Smart Electrical Grid with Federated Detection, Dynamic Risk Assessment and Self-Healing. Proceedings of the 18th International Conference on Availability, Reliability and Security (ARES ’23).

[25] Shabir M.M., Danish S.M., Zhang K. (2023). BlockQoS: Fair Monetization of On-demand Quality-of-Service using Blockchains. Distrib. Ledger Technol..

[26] Abbasi A.G., Khan Z. VeidBlock: Verifiable Identity Using Blockchain and Ledger in a Software Defined Network. Proceedings of the Companion Proceedings of the 10th International Conference on Utility and Cloud Computing.

[27] Gupta R., Patel M.M., Tanwar S., Kumar N., Zeadally S. (2021). Blockchain-Based Data Dissemination Scheme for 5G-Enabled Softwarized UAV Networks. IEEE Trans. Green Commun. Netw..

[28] Bai L., Liu L. Research on Software Defined Network Security Model Based on Blockchain. Proceedings of the 2021 6th International Conference on Intelligent Computing and Signal Processing (ICSP).

[29] Chattaraj D., Saha S., Bera B., Das A.K. On the Design of Blockchain-Based Access Control Scheme for Software Defined Networks. Proceedings of the IEEE INFOCOM 2020—IEEE Conference on Computer Communications Workshops (INFOCOM WKSHPS).

[30] Das D., Banerjee S., Dasgupta K., Chatterjee P., Ghosh U., Biswas U. Blockchain Enabled SDN Framework for Security Management in 5G Applications. Proceedings of the 24th International Conference on Distributed Computing and Networking.

[31] Ghosh U., Njilla L., Shetty S., Kamhoua C.A. A Decentralized Smart Grid Communication Framework Using SDN-Enabled Blockchain. Proceedings of the 2024 IEEE 21st Consumer Communications & Networking Conference (CCNC).

[32] Hakiri A., Dezfouli B. (2021). Towards a Blockchain-SDN Architecture for Secure and Trustworthy 5G Massive IoT Networks. Proceedings of the 2021 ACM International Workshop on Software Defined Networks & Network Function Virtualization Security.

[33] Indrason N., Pohrmen F.H., Marshoodulla S.Z., Saha G. Blockchain and SDN-IoT based secured voting system. Proceedings of the 2023 4th International Conference on Computing and Communication Systems (I3CS).

[34] Cao J., Wang X., Huang M., Yi B., He Q. (2021). A security-driven network architecture for routing in industrial Internet of Things. Trans. Emerg. Telecommun. Technol..

[35] Li J., Zhu M., Liu J., Liu W., Huang B., Liu R. (2024). Blockchain-based reliable task offloading framework for edge-cloud cooperative workflows in IoMT. Inf. Sci..

[36] Singh M.P., Singh A., Aujla G.S., Singh Bali R., Jindal A. Referenced Blockchain Approach for Road Traffic Monitoring in a Smart City using Internet of Drones. Proceedings of the ICC 2022—IEEE International Conference on Communications.

[37] Aldaej A., Atiquzzaman M., Ahanger T.A., Shukla P.K. (2023). Multidomain blockchain-based intelligent routing in UAV-IoT networks. Comput. Commun..

[38] Garg D., Bali R.S. (2024). Edge Supported QoS based Secure Data Communication mechanism for Software Defined Vehicular Networks. Proceedings of the 25th International Conference on Distributed Computing and Networking.

[39] Li J., Chen Y., Chen Y., Zhang W., Liu Z. (2023). A smart energy IoT model based on the Itsuku PoW technology. Results Eng..

[40] Podili P., Kataoka K. (2021). TRAQR: Trust aware End-to-End QoS routing in multi-domain SDN using Blockchain. J. Netw. Comput. Appl..

[41] Singh S., Mehla V., Nikolovski S. (2022). LSSDNF: A Lightweight Secure Software Defined Network Framework for Future Internet in 5G–6G. Future Internet.

[42] Abdulqadder I.H., Zhou S., Zou D., Aziz I.T., Akber S.M.A. Bloc-Sec: Blockchain-Based Lightweight Security Architecture for 5G/B5G Enabled SDN/NFV Cloud of IoT. Proceedings of the 2020 IEEE 20th International Conference on Communication Technology (ICCT).

[43] Bose A., Aujla G.S., Singh M., Kumar N., Cao H. Blockchain as a Service for Software Defined Networks: A Denial of Service Attack Perspective. Proceedings of the 2019 IEEE International Conference on Dependable, Autonomic and Secure Computing, International Conference on Pervasive Intelligence and Computing, International Conference on Cloud and Big Data Computing, International Conference on Cyber Science and Technology Congress (DASC/PiCom/CBDCom/CyberSciTech).

[44] Li Z., Ding Y., Gao H., Qu B., Wang Y., Li J. (2023). A Highly Compatible Verification Framework with Minimal Upgrades to Secure an Existing Edge Network. ACM Trans. Internet Technol..

[45] Xu R., Chen Y., Chen G., Blasch E. (2022). SAUSA: Securing Access, Usage, and Storage of 3D Point CloudData by a Blockchain-Based Authentication Network. Future Internet.

[46] Cheriet A., Mekhaznia T. Secure Mobile Data Offloading in Small Cell Networks. Proceedings of the 2022 4th International Conference on Pattern Analysis and Intelligent Systems (PAIS).

[47] Hasan K., Chowdhury M.J.M., Biswas K., Ahmed K., Islam M.S., Usman M. (2022). A blockchain-based secure data-sharing framework for Software Defined Wireless Body Area Networks. Comput. Netw..

[48] Ragu G., Ramamoorthy S. (2023). A blockchain-based cloud forensics architecture for privacy leakage prediction with cloud. Healthc. Anal..

[49] Abdulqadder I.H., Zou D., Aziz I.T. (2023). The DAG blockchain: A secure edge assisted honeypot for attack detection and multi-controller based load balancing in SDN 5G. Future Gener. Comput. Syst..

[50] Algarni S., Eassa F., Almarhabi K., Algarni A., Albeshri A. (2022). BCNBI: A Blockchain-Based Security Framework for Northbound Interface in Software-Defined Networking. Electronics.

[51] Asaithambi S., Ravi L., Kotb H., Milyani A.H., Azhari A.A., Nallusamy S., Varadarajan V., Vairavasundaram S. (2022). An Energy-Efficient and Blockchain-Integrated Software Defined Network for the Industrial Internet of Things. Sensors.

[52] Basnet S.R., Shakya S. (2017). BSS: Blockchain security over software defined network. Proceedings of the 2017 International Conference on Computing, Communication and Automation (ICCCA).

[53] Bhardwaj A., Chaudhary R., Aslam A.M., Budhiraja I. Blockchain-based Robust SDN Framework for Digital Twin-Enabled IoT Networks. Proceedings of the 2023 IEEE 98th Vehicular Technology Conference (VTC2023-Fall).

[54] Rodrigues B., Stiller B. The Cooperative DDoS Signaling based on a Blockchain-based System. Proceedings of the 2021 IFIP/IEEE International Symposium on Integrated Network Management (IM).

[55] Fan W., Park Y., Kumar S., Ganta P., Zhou X., Chang S.Y. Blockchain-Enabled Collaborative Intrusion Detection in Software Defined Networks. Proceedings of the 2020 IEEE 19th International Conference on Trust, Security and Privacy in Computing and Communications (TrustCom).

[56] Meng W., Li W., Zhou J. (2021). Enhancing the security of blockchain-based software defined networking through trust-based traffic fusion and filtration. Inf. Fusion.

[57] Matheu S.N., Robles Enciso A., Molina Zarca A., Garcia-Carrillo D., Hernández-Ramos J.L., Bernal Bernabe J., Skarmeta A.F. (2020). Security Architecture for Defining and Enforcing Security Profiles in DLT/SDN-Based IoT Systems. Sensors.

[58] Sezer S., Scott-Hayward S., Chouhan P., Fraser B., Lake D., Finnegan J., Viljoen N., Miller M., Rao N. (2013). Are we ready for SDN? Implementation challenges for software-defined networks. Commun. Mag. IEEE.

[59] Kirkpatrick K. (2013). Software-defined networking. Commun. ACM.

[60] de Oliveira Silva F., de Souza Pereira J., Rosa P., Kofuji S. Enabling Future Internet Architecture Research and Experimentation by Using Software Defined Networking. Proceedings of the 2012 European Workshop on Software Defined Networking (EWSDN).

[61] Goth G. (2011). Software-Defined Networking Could Shake up More than Packets. Internet Comput. IEEE.

[62] Xia W., Wen Y., Foh C., Niyato D., Xie H. (2014). A Survey on Software-Defined Networking. Commun. Surv. Tutor..

[63] Karakus M., Durresi A. A Scalability Metric for Control Planes in Software Defined Networks (SDNs). Proceedings of the Advanced Information Networking and Applications (AINA).

[64] Lin P., Bi J., Wolff S., Wang Y., Xu A., Chen Z., Hu H., Lin Y. (2015). A West-East Bridge Based SDN Inter-Domain Testbed. Commun. Mag. IEEE.

[65] Bakshi K. (2013). Considerations for Software Defined Networking (SDN): Approaches and use cases. Proceedings of the Aerospace Conference.

[66] Jarraya Y., Madi T., Debbabi M. (2014). A Survey and a Layered Taxonomy of Software-Defined Networking. IEEE Commun. Surv. Tutor..

[67] Nunes B.A.A., Mendonca M., Nguyen X.N., Obraczka K., Turletti T. (2014). A Survey of Software-Defined Networking: Past, Present, and Future of Programmable Networks. Commun. Surv. Tutor..

[68] McKeown N., Anderson T., Balakrishnan H., Parulkar G., Peterson L., Rexford J., Shenker S., Turner J. (2008). OpenFlow: Enabling Innovation in Campus Networks. SIGCOMM Comput. Commun. Rev..

[69] Hu F., Hao Q., Bao K. (2014). A Survey on Software Defined Networking (SDN) and OpenFlow: From Concept to Implementation. IEEE Commun. Surv. Tutor..

[70] Lara A., Kolasani A., Ramamurthy B. (2014). Network Innovation using OpenFlow: A Survey. Commun. Surv. Tutor..

[71] Vaughan-Nichols S.J. (2011). OpenFlow: The Next Generation of the Network?. IEEE Comput..

[72] ONF (2012). Software-Defined Networking: The New Norm for Networks.

[73] Open Networking Foundation (2015). OpenFlow Switch Specification (1.5.1). http://rfc.nop.hu/openflow/openflow-switch-v1.5.1.pdf.

[74] Gervais A., Karame G.O., Wüst K., Glykantzis V., Ritzdorf H., Capkun S. On the security and performance of proof of work blockchains. Proceedings of the 2016 ACM SIGSAC Conference on Computer and Communications Security.

[75] Saleh F. (2021). Blockchain without Waste: Proof-of-Stake. Rev. Financ. Stud..

[76] Yang F., Zhou W., Wu Q., Long R., Xiong N.N., Zhou M. (2019). Delegated Proof of Stake with Downgrade: A Secure and Efficient Blockchain Consensus Algorithm with Downgrade Mechanism. IEEE Access.

[77] De Angelis S., Aniello L., Baldoni R., Lombardi F., Margheri A., Sassone V. PBFT vs. proof-of-authority: Applying the CAP theorem to permissioned blockchain. Proceedings of the CEUR Workshop Proceedings.

[78] Castro M., Liskov B. (2002). Practical byzantine fault tolerance and proactive recovery. ACM Trans. Comput. Syst. (TOCS).

[79] Pourvahab M., Ekbatanifard G. (2019). An Efficient Forensics Architecture in Software-Defined Networking-IoT Using Blockchain Technology. IEEE Access.

[80] Cloudflare What Is the Mirai Botnet? 2020. https://www.cloudflare.com/learning/ddos/glossary/mirai-botnet/.

[81] Guerrero-Saade J.A. (2022). AcidRain|A Modem Wiper Rains Down on Europe. https://www.sentinelone.com/labs/acidrain-a-modem-wiper-rains-down-on-europe/.

[82] Team W.C. (2024). Wazirx Cyber Attack Daywise Report. https://wazirx.com/blog/wazirx-cyber-attack-day-wise-report/.

[83] Ai Q. What Happened with the 570 Million Binance (BNB) Hack? And What Does It Really Mean for Crypto Investors? 2022. https://www.forbes.com/sites/qai/2022/10/09/what-happened-with-the-570-million-binance-bnb-hack-and-what-does-it-really-mean-for-crypto-investors/.

[84] Faife C. (2022). Nomad Bridge 200 Million Chaotic Hack Smartcontract Cryptocurrency. https://www.theverge.com/2022/8/2/23288785/nomad-bridge-200-million-chaotic-hack-smart-contract-cryptocurrency.

[85] Reddick J.G.J. (2024). Courtroom Recording Software Compromised with Backdoor Installer. https://therecord.media/courtroom-recording-software-compromised-backdoor.

[86] Sharma A. (2024). Router Maker’s Support Portal Hacked, Replies with MetaMask Phishing. https://www.bleepingcomputer.com/news/security/router-makers-support-portal-hacked-replies-with-metamask-phishing/.

[87] Arghire I. Hackers Exploit Flaw in Squarespace Migration to Hijack Domains, 2024. https://www.securityweek.com/hackers-exploit-flaw-in-squarespace-migration-to-hijack-domains/.

[88] Gao S., Li Z., Xiao B., Wei G. (2018). Security Threats in the Data Plane of Software-Defined Networks. IEEE Netw..

[89] Febro A., Xiao H., Spring J., Christianson B. (2022). Synchronizing DDoS defense at network edge with P4, SDN, and Blockchain. Comput. Netw..

[90] El Houda Z.A., Hafid A., Khoukhi L. Co-IoT: A Collaborative DDoS Mitigation Scheme in IoT Environment Based on Blockchain Using SDN. Proceedings of the 2019 IEEE Global Communications Conference (GLOBECOM).

[91] Islam M.J., Rahman A., Kabir S., Karim M.R., Acharjee U.K., Nasir M.K., Band S.S., Sookhak M., Wu S. (2022). Blockchain-SDN-Based Energy-Aware and Distributed Secure Architecture for IoT in Smart Cities. IEEE IoT J..

[92] Duy P.T., Quyen N.H., Khoa N.H., Tran T.D., Pham V.H. (2023). FedChain-Hunter: A reliable and privacy-preserving aggregation for federated threat hunting framework in SDN-based IIoT. Internet Things.

[93] Kumar S., Singh A., Benslimane A., Chithaluru P., Albahar M.A., Rathore R.S., Álvarez R.M. (2023). An Optimized Intelligent Computational Security Model for Interconnected Blockchain-IoT System & Cities. Ad Hoc Netw..

[94] Rahman A., Islam M.J., Rahman Z., Reza M.M., Anwar A., Mahmud M.A.P., Nasir M.K., Noor R.M. (2020). DistB-Condo: Distributed Blockchain-Based IoT-SDN Model for Smart Condominium. IEEE Access.

[95] Rahman A., Islam M.J., Sunny F.A., Nasir M.K. DistBlockSDN: A Distributed Secure Blockchain Based SDN-IoT Architecture with NFV Implementation for Smart Cities. Proceedings of the 2019 2nd International Conference on Innovation in Engineering and Technology (ICIET).

[96] Rahman A., Islam M.J., Montieri A., Nasir M.K., Reza M.M., Band S.S., Pescape A., Hasan M., Sookhak M., Mosavi A. (2021). SmartBlock-SDN: An Optimized Blockchain-SDN Framework for Resource Management in IoT. IEEE Access.

[97] Razaque A., Yoo J., Bektemyssova G., Alshammari M., Chinibayeva T.T., Amanzholova S., Alotaibi A., Umutkulov D. (2023). Efficient Internet-of-Things Cyberattack Depletion Using Blockchain-Enabled Software-Defined Networking and 6G Network Technology. Sensors.

[98] Guha Roy D., Srirama S.N. (2021). A Blockchain-based Cyber Attack Detection Scheme for Decentralized Internet of Things using Software-Defined Network. Softw. Pract. Exper..

[99] Sumathi A.C., Ahalawat A., Rameshkumar A. (2022). Early detection of DDoS attack using integrated SDN-Blockchain architecture for IoT. Proceedings of the 2022 International Conference on Innovative Computing, Intelligent Communication and Smart Electrical Systems (ICSES).

[100] Derhab A., Guerroumi M., Gumaei A., Maglaras L., Ferrag M.A., Mukherjee M., Khan F.A. (2019). Blockchain and Random Subspace Learning-Based IDS for SDN-Enabled Industrial IoT Security. Sensors.

[101] Poorazad S.K., Benzaïd C., Taleb T. Blockchain and Deep Learning-Based IDS for Securing SDN-Enabled Industrial IoT Environments. Proceedings of the GLOBECOM 2023—2023 IEEE Global Communications Conference.

[102] Jmal R., Ghabri W., Guesmi R., Alshammari B.M., Alshammari A.S., Alsaif H. (2023). Distributed Blockchain-SDN Secure IoT System Based on ANN to Mitigate DDoS Attacks. Appl. Sci..

[103] Huo L., Jiang D., Qi S., Miao L. (2020). A Blockchain-Based Security Traffic Measurement Approach to Software Defined Networking. Mob. Netw. Appl..

[104] Su J., Jiang M. (2023). A Hybrid Entropy and Blockchain Approach for Network Security Defense in SDN-Based IIoT. Chin. J. Electron..

[105] Das D., Ghosh U., Evans N., Shetty S. (2024). Blockchain-enabled secure device-to-device communication in software-defined networking. Proceedings of the 2024 IEEE International Conference on Communications Workshops (ICC Workshops).

[106] Xu R., Nagothu D., Chen Y., Aved A., Ardiles-Cruz E., Blasch E. (2024). A Secure Interconnected Autonomous System Architecture for Multi-Domain IoT Ecosystems. IEEE Commun. Mag..

[107] Pawar P.P., Kumar D., Ananthan B., Pradeepa A.S., Selvi A.S. (2024). An efficient ddos attack detection using attention based hybrid model in blockchain based SDN-IOT. Proceedings of the 2024 3rd International Conference on Artificial Intelligence for Internet of Things (AIIoT).

[108] MathalaiRaj J., Sivaranjani S., Rajalakshmi J., Jayachandran T., Maheswari M., Chandrasekaran G. (2024). Block chain and Deep Learning Based Secure Communication using SAE-LSTM &Salp Swarm Optimizer for Multivariate Industrial IoT-oriented Infrastructure. Proceedings of the 2024 15th International Conference on Computing Communication and Networking Technologies (ICCCNT).

[109] Aguru A.D., Erukala S.B. (2024). Blockchain-based edge device authentication mechanism in sdn-enabled iot networks. Proceedings of the 2024 IEEE 9th International Conference for Convergence in Technology (I2CT).

[110] Gadekallu T.R., Fang K., Feng H., Pandya S., Murugan R., Maddikunta P.K.R., Bhattacharya S., Yenduri G. (2024). Blockchain for Securing Device-to-Device Communication in Software Defined Networks. Proceedings of the 2024 IEEE Globecom Workshops (GC Wkshps).

[111] Tian J., Shu Z., Chen S., Xie H., Liu X., Qiu C. (2024). Enhanced DDoS Defense in SDN: Double-Layered Strategy with Blockchain Integration. Proceedings of the 2024 13th International Conference on Communications, Circuits and Systems (ICCCAS).

[112] Jain A., Khatri D.K., Ayyagiri A., Mokkapati C., Bhimanapati V.B.R., Alzubaidi L.H. (2024). Secure and Scalable IoT Networks: Optimizing Blockchain and SDN for Smart Environments. Proceedings of the 2024 4th International Conference on Blockchain Technology and Information Security (ICBCTIS).

[113] Dautov D., Khayretdinov R., Vulfin A., Mironov K. Distributed Ledger Methods in Securing Software-Defined Networks. Proceedings of the 2021 3rd International Conference on Control Systems, Mathematical Modeling, Automation and Energy Efficiency (SUMMA).

[114] Liu L., Feng W., Chen C., Zhang Y., Lan D., Yuan X., Vashisht S. BS-IoT: Blockchain Based Software Defined Network Framework for Internet of Things. Proceedings of the IEEE INFOCOM 2020—IEEE Conference on Computer Communications Workshops (INFOCOM WKSHPS).

[115] Kumaran U., Swetha T., Sharma I., Keerthana R., Tejashwini V., Devarajan G.G. (2024). Enhancing cloud iot security with blockchain and sdn. Proceedings of the 2024 International Conference on Communication, Computer Sciences and Engineering (IC3SE).

[116] Yazdinejad A., Parizi R.M., Dehghantanha A., Zhang Q., Choo K.K.R. (2020). An Energy-Efficient SDN Controller Architecture for IoT Networks with Blockchain-Based Security. IEEE Trans. Serv. Comput..

[117] Hameed S., Shah S.A., Saeed Q.S., Siddiqui S., Ali I., Vedeshin A., Draheim D. (2021). A Scalable Key and Trust Management Solution for IoT Sensors Using SDN and Blockchain Technology. IEEE Sens. J..

[118] Manocha P.S., Kumar R. (2022). Improved spider monkey optimization-based multi-objective software-defined networking routing with block chain technology for Internet of Things security. Concurr. Comput. Pract. Exp..

[119] Kumar P., Kumar R., Aljuhani A., Javeed D., Jolfaei A., Islam A.K.M.N. (2023). Digital twin-driven SDN for smart grid: A deep learning integrated blockchain for cybersecurity. Sol. Energy.

[120] Ghamdi M.A.A. (2022). An Optimized and Secure Energy-Efficient Blockchain-Based Framework in IoT. IEEE Access.

[121] Mathalai Raj J., Siva Ranjani S. (2023). A secured blockchain method for multivariate industrial IoT-oriented infrastructure based on deep residual squeeze and excitation network with single candidate optimizer. Internet Things.

[122] Rani S., Babbar H., Srivastava G., Gadekallu T.R., Dhiman G. (2023). Security Framework for Internet-of-Things-Based Software-Defined Networks Using Blockchain. IEEE Internet Things J..

[123] Latif S.A., Wen F.B.X., Iwendi C., Wang L.L.-F., Mohsin S.M., Han Z., Band S.S. (2022). AI-empowered, blockchain and SDN integrated security architecture for IoT network of cyber physical systems. Comput. Commun..

[124] Akhyani J., Patel J., Desai V., Gupta R., Tanwar S., Bhatia J. (2024). GRACE: Blockchain and Game-Based Resource Allocation Scheme for SDN Controllers in ioT. Proceedings of the 2024 IEEE International Conference on Communications Workshops (ICC Workshops).

[125] Khan A.F., Nanda P. Hybrid blockchain-based Authentication Handover and Flow Rule Validation for Secure Software Defined 5G HetNets. Proceedings of the 2022 International Wireless Communications and Mobile Computing (IWCMC).

[126] Abdulqadder I.H., Zhou S. (2022). SliceBlock: Context-Aware Authentication Handover and Secure Network Slicing Using DAG-Blockchain in Edge-Assisted SDN/NFV-6G Environment. IEEE Internet Things J..

[127] Bhattacharya P., Shukla A., Tanwar S., Kumar N., Sharma R. (2022). 6Blocks: 6G-enabled trust management scheme for decentralized autonomous vehicles. Comput. Commun..

[128] Xie L., Ding Y., Yang H., Wang X. (2019). Blockchain-Based Secure and Trustworthy Internet of Things in SDN-Enabled 5G-VANETs. IEEE Access.

[129] Krishnan P., Jain K., Poojara S.R., Srirama S.N., Pandey T., Buyya R. (2024). eSIM and blockchain integrated secure zero-touch provisioning for autonomous cellular-IoTs in 5G networks. Comput. Commun..

[130] Shao S., Gong W., Yang H., Guo S., Chen L., Xiong A. (2023). Data Trusted Sharing Delivery: A Blockchain-Assisted Software-Defined Content Delivery Network. IEEE Internet Things J..

[131] Okon A.A., Sallam K.M., Hossain M.F., Jagannath N., Jamalipour A., Munasinghe K.S. (2024). Enhancing Multi-Operator Network Handovers with Blockchain-Enabled SDN Architectures. IEEE Access.

[132] Balachandran C., C P.A., Ramachandran G., Krishnamachari B. EDISON: A Blockchain-based Secure and Auditable Orchestration Framework for Multi-domain Software Defined Networks. Proceedings of the 2020 IEEE International Conference on Blockchain (Blockchain).

[133] Abdulqadder I.H., Zhou S., Aziz I.T., Zou D., Deng X., Abrar Akber S.M. An Effective Lightweight Intrusion Detection System with Blockchain to Mitigate Attacks in SDN/NFV Enabled Cloud. Proceedings of the 2021 6th International Conference for Convergence in Technology (I2CT).

[134] Bhattacharya P., Singh S.K., Liu H., Gadekallu T.R. (2024). Trusted SDN-enabled Resource Allocation Scheme in Edge-Cloud Interplay for 6G-assisted Smart Cities. Proceedings of the 2024 IEEE Globecom Workshops (GC Wkshps).

[135] Choudhary S., Dorle S. SDN based Blockchain Architecture for Security Performance of VANETs. Proceedings of the 2021 International Conference on Computational Intelligence and Computing Applications (ICCICA).

[136] Kaur K., Kaddoum G., Zeadally S. (2021). Blockchain-Based Cyber-Physical Security for Electrical Vehicle Aided Smart Grid Ecosystem. IEEE Trans. Intell. Transp. Syst..

[137] Choudhary S., Dorle S. (2022). A quality of service-aware high-security architecture design for software-defined network powered vehicular ad-hoc networks using machine learning-based blockchain routing. Concurr. Comput. Pract. Exp..

[138] Rahman A., Eidmum M.Z.A., Kundu D., Hossain M., Tashrif M.T.A., Karim M.A., Islam M.J. (2024). Distb-vnet: Distributed cluster-based blockchain vehicular ad-hoc networks through sdn-nfv for smart city. Proceedings of the 2024 27th International Conference on Computer and Information Technology (ICCIT).

[139] Ulhe P., Asole S. Design of an Improved Model for IoV Security Using Bioinspired Optimization & Scalable Blockchains. Proceedings of the 2024 2nd DMIHER International Conference on Artificial Intelligence in Healthcare, Education and Industry (IDICAIEI).

[140] Chaudhary R., Jindal A., Aujla G.S., Aggarwal S., Kumar N., Choo K.K.R. (2019). BEST: Blockchain-based secure energy trading in SDN-enabled intelligent transportation system. Comput. Secur..

[141] Gao J., Obour Agyekum K.O.B., Sifah E.B., Acheampong K.N., Xia Q., Du X., Guizani M., Xia H. (2020). A Blockchain-SDN-Enabled Internet of Vehicles Environment for Fog Computing and 5G Networks. IEEE Internet Things J..

[142] Wang Z., Xu Y., Liu J., Li Z., Li Z., Jia H., Wang D. (2022). An Efficient Data Sharing Scheme for Privacy Protection Based on Blockchain and Edge Intelligence in 6G-VANET. Wirel. Commun. Mob. Comput..

[143] Vishwakarma L., Nahar A., Das D. (2022). LBSV: Lightweight Blockchain Security Protocol for Secure Storage and Communication in SDN-Enabled IoV. IEEE Trans. Veh. Technol..

[144] Lin H., Garg S., Hu J., Kaddoum G., Peng M., Hossain M.S. (2021). Blockchain and Deep Reinforcement Learning Empowered Spatial Crowdsourcing in Software-Defined Internet of Vehicles. IEEE Trans. Intell. Transp. Syst..

[145] Swarup L. The Edge Cloud Computing for a Secure and QoS-Aware Internet of Things Vehicular Network. Proceedings of the 2023 3rd International Conference on Smart Generation Computing, Communication and Networking (SMART GENCON).

[146] Singh S.K., Jeong Y.S., Park J.H. (2020). A deep learning-based IoT-oriented infrastructure for secure smart City. Sustain. Cities Soc..

[147] Medhane D.V., Sangaiah A.K., Hossain M.S., Muhammad G., Wang J. (2020). Blockchain-Enabled Distributed Security Framework for Next-Generation IoT: An Edge Cloud and Software-Defined Network-Integrated Approach. IEEE Internet Things J..

[148] Saba T., Haseeb K., Rehman A., Jeon G. (2024). Blockchain-Enabled Intelligent IoT Protocol for High-Performance and Secured Big Financial Data Transaction. IEEE Trans. Comput. Soc. Syst..

[149] Sharma P.K., Chen M.Y., Park J.H. (2018). A Software Defined Fog Node Based Distributed Blockchain Cloud Architecture for IoT. IEEE Access.

[150] Rahman A., Islam M.J., Islam R., Aziz A., Kundu D., Sazzad S., Karim M.R., Hasan M., Rahman Z., Elnaffar S. (2022). Enhancing Data Security for Cloud Computing Applications through Distributed Blockchain-based SDN Architecture in IoT Networks. arXiv.

[151] Haritha K., Vellela S.S., D R., Vuyyuru L.R., Malathi N., Dalavai L. Distributed Blockchain-SDN Models for Robust Data Security in Cloud-Integrated IoT Networks. Proceedings of the 2024 3rd International Conference on Automation, Computing and Renewable Systems (ICACRS).

[152] Zhao D., Zhang D., Pei Q., Liu L., Yue P. (2024). Blockchain-Based Security Deployment and Resource Allocation in SDN-Enabled MEC System. IEEE Internet Things J..

[153] Sibiya K., Molefe M., Nleya B. (2024). A SDN multi-controller and blockchain enabled authentication framework for cloud computing. Proceedings of the 2024 International Conference on Electrical, Computer and Energy Technologies (ICECET).

[154] Khoshjahan M., Kezunovic M. (2022). Cybersecurity analysis of prosumer/aggregator communications VIA software defined networking emulators. Proceedings of the CIRED Porto Workshop 2022: E-Mobility and Power Distribution Systems.

[155] Xiong A., Tian H., He W., Zhang J., Meng H., Guo S., Wang X., Wu X., Kadoch M. (2021). A distributed security SDN cluster architecture for smart grid based on blockchain technology. Secur. Commun. Netw..

[156] Alsuwaidi N., Alharmoodi N., Al Hamadi H. (2024). Securing Smart Grid Infrastructures: Challenges, Defense Mechanisms, and Future Directions. Proceedings of the 2024 IEEE Future Networks World Forum (FNWF).

[157] Lu X., Shi L., Chen Z., Fan X., Guan Z., Du X., Guizani M. (2019). Blockchain-Based Distributed Energy Trading in Energy Internet: An SDN Approach. IEEE Access.

[158] Rifat M.H., Ananna A.I., Ahmed T.I., Akter S., Mansoor N. (2024). Blockchain-based controller recovery and SDN packet filtering scheme for softwarized UAVs. Proceedings of the 2024 International Conference on Advances in Computing, Communication, Electrical, and Smart Systems (iCACCESS).

[159] Kumar P., Kumar R., Kumar A., Franklin A.A., Jolfaei A. Blockchain and Deep Learning Empowered Secure Data Sharing Framework for Softwarized UAVs. Proceedings of the 2022 IEEE International Conference on Communications Workshops (ICC Workshops).

[160] Ren J., Li J., Liu H., Qin T. (2022). Task offloading strategy with emergency handling and blockchain security in SDN-empowered and fog-assisted healthcare IoT. Tsinghua Sci. Technol..

[161] Goel O., Gajbhiye B., Gangu K., Avancha S., Thumati P.R.R., Hussein L. (2024). A Secure and Efficient Blockchain Protocol for Protecting Electronic Health Records. Proceedings of the 2024 4th International Conference on Blockchain Technology and Information Security (ICBCTIS).

[162] Siddiqui S., Hameed S., Shah S.A., Khan A.K., Aneiba A. (2023). Smart contract-based security architecture for collaborative services in municipal smart cities. J. Syst. Archit..

[163] Rehman A., Haseeb K., Alam T., Alamri F.S., Saba T., Song H. (2023). Intelligent secured traffic optimization model for urban sensing applications with Software Defined Network. IEEE Sens. J..

[164] Jha M. (2022). Secure SDN Based IoT Network through Blockchain for Smart Architectures. Proceedings of the 2022 IEEE Region 10 Symposium (TENSYMP).

[165] Alotaibi J. (2025). A hybrid software-defined networking approach for enhancing IoT cybersecurity with deep learning and blockchain in smart cities. Peer-Netw. Appl..

[166] Aujla G.S., Singh M., Bose A., Kumar N., Han G., Buyya R. (2020). BlockSDN: Blockchain-as-a-Service for Software Defined Networking in Smart City Applications. IEEE Netw..

[167] Mukhejee A., Chatterjee R., Mandal J.K. (2022). A Proposed Technique for Management of Flow Rules in SDN using Blockchain. Proceedings of the 2022 International Conference on Electrical, Computer, Communications and Mechatronics Engineering (ICECCME).

[168] Tang C., Zhao B., Chen Y., Zhou H. (2024). DABS: A Distributed Attribute-Based Access Control Mechanism for SD-WAN Controllers Based on Smart Contracts. Proceedings of the 2024 International Conference on Meta Computing (ICMC).

[169] Fan W., Chang S.Y., Kumar S., Zhou X., Park Y. Blockchain-based Secure Coordination for Distributed SDN Control Plane. Proceedings of the 2021 IEEE 7th International Conference on Network Softwarization (NetSoft).

[170] Rivera J.J.D., Vilalta R., Muñoz R., Alemany P., Renom L.G. (2024). Distributed Trust for Collaborative Network Management: Leveraging DLT in Multi-SDN Controller Environments. Proceedings of the 2024 IEEE Conference on Network Function Virtualization and Software Defined Networks (NFV-SDN).

[171] Lokesh B., Rajagopalan N. A Blockchain-based security model for SDNs. Proceedings of the 2020 IEEE International Conference on Electronics, Computing and Communication Technologies (CONECCT).

[172] Latah M., Kalkan K. (2024). SDN-API-Sec: A Conflict-Free BC-Based Authorization for Cross-Domain SDNs. Proceedings of the 2024 8th Cyber Security in Networking Conference (CSNet).

[173] Yaseen F.A., Alkhalidi N.A., Al-Raweshidy H.S. (2023). Itor-sdn: Intelligent tor networks-based sdn for data forwarding management. IEEE Access.

[174] Rettore P.H., Zißner P., Lawisch M., Kloth S., de Freitas E.P., Sevenich P. (2024). Towards a resilient multi-agent controller: Securing and mitigating overhead in tactical sdn. Proceedings of the 2024 11th International Conference on Software Defined Systems (SDS).

[175] Bajpai A., Singh A., Kansal V., Prakash S., Yang T., Rathore R.S. (2024). Blockchain-Enabled Real-Time Intrusion Detection Framework for a Cyber-Physical System. Proceedings of the 2024 International Conference on Decision Aid Sciences and Applications (DASA).

[176] Erel-Ozcevik M. (2023). Sustainable fixed wireless access with blockchain secured software defined network. Pervasive Mob. Comput..

[177] Zhang Y., Li C., Chen N., Zhang P. Intelligent Requests Orchestration for Microservice Management Based on Blockchain in Software Defined Networking: A Security Guarantee. Proceedings of the 2022 IEEE International Conference on Communications Workshops.

[178] Khan Y., Verma S. (2021). An Intelligent Blockchain and Software-Defined Networking-Based Evidence Collection Architecture for Cloud Environment. Sci. Program..

[179] Tuan D.T., Duy P.T., Hau L.C., Pham V.H. A Blockchain-based Authentication and Access Control for Smart Devices in SDN-enabled Networks for Metaverse. Proceedings of the 2022 9th NAFOSTED Conference on Information and Computer Science (NICS).

[180] Chattaraj D., Bera B., Das A.K., Rodrigues J.J.P.C., Park Y. (2022). Designing Fine-Grained access Control for Software-Defined Networks Using Private Blockchain. IEEE Internet Things J..

[181] Duy P.T., Hoang H.D., Hien D.T.T., Nguyen A.G.T., Pham V.H. (2022). B-DAC: A decentralized access control framework on Northbound interface for securing SDN using blockchain. J. Inf. Secur. Appl..

[182] Jiang B., He Q., He M., Zhai Z., Zhao B. (2023). FACSC: Fine-Grained access Control Based on Smart Contract for Terminals in Software-Defined Network. Secur. Commun. Netw..

[183] Ren W., Sun Y., Luo H., Guizani M. (2021). SILedger: A Blockchain and ABE-based Access Control for Applications in SDN-IoT Networks. IEEE Trans. Netw. Serv. Manag..

[184] Reddy C.M.K., Chandrashekar R., Kannan K.N., Pal Thethi H., Dharmireddi S., Bajaj R. Smart Contracts and Anomaly Detection in SDN environment using Cloud-Edge Integration Model. Proceedings of the 2023 International Conference on Emerging Research in Computational Science (ICERCS).

[185] Rahman A., Islam M.J., Saikat Islam Khan M., Kabir S., Pritom A.I., Razaul Karim M. Block-SDoTCloud: Enhancing Security of Cloud Storage through Blockchain-based SDN in IoT Network. Proceedings of the 2020 2nd International Conference on Sustainable Technologies for Industry 4.0 (STI).

[186] Ming He L., He Q., Ya Guo N., Kuang Qin Y. ABSA: A Blockchain-based SDN Device Access Control Scheme. Proceedings of the 2021 10th International Conference on Internet Computing for Science and Engineering.

[187] Khalid M., Hameed S., Qadir A., Shah S.A., Draheim D. (2023). Towards SDN-based smart contract solution for IoT access control. Comput. Commun..

[188] Mendiboure L., Chalouf M.A., Krief F. A Scalable Blockchain-based Approach for Authentication and access Control in Software Defined Vehicular Networks. Proceedings of the 2020 29th International Conference on Computer Communications and Networks (ICCCN).

[189] Hu J., Reed M., Al-Naday M., Thomos N. Blockchain-Aided Flow Insertion and Verification in Software Defined Networks. Proceedings of the 2020 Global Internet of Things Summit (GIoTS).

[190] Latah M., Kalkan K. DPSec: A blockchain-based data plane authentication protocol for SDNs. Proceedings of the 2020 Second International Conference on Blockchain Computing and Applications (BCCA).

[191] Krishnan P., Jain K., Achuthan K., Buyya R. (2022). Software-Defined Security-by-Contract for Blockchain-Enabled MUD-Aware Industrial IoT Edge Networks. IEEE Trans. Ind. Inform..

[192] Li C. (2024). Research on Security Authentication Method for Distributed SDN Network Data Transmission Based on Alliance Blockchain. Proceedings of the 2024 4th International Conference on Computer Science, Electronic Information Engineering and Intelligent Control Technology (CEI).

[193] Lei X., Song Y., Lin J., Feng T., Li P., Wang Y., Yang C. Resilience In-Band Control Path Routing in Blockchain-Based Multi-Domain SDN. Proceedings of the 2023 International Wireless Communications and Mobile Computing (IWCMC).

[194] Ali J., Shan G., Gul N., Roh B.H. (2023). An Intelligent Blockchain-based Secure Link Failure Recovery Framework for Software-defined Internet-of-Things. J. Grid Comput..

[195] Zhang P., Liu F., Kumar N., Aujla G.S. Information Classification Strategy for Blockchain-based Secure SDN in IoT Scenario. Proceedings of the IEEE INFOCOM 2020—IEEE Conference on Computer Communications Workshops (INFOCOM WKSHPS).

[196] Martinello M., Gomes R.L., Borges E.S., Layber H.C., Bonella V.B., Dominicini C.K., Guimarães R., Ribeiro M., Barcellos M. (2024). PathSec: Path-Aware Secure Routing with Native Path Verification and Auditability. Proceedings of the 2024 IEEE Conference on Network Function Virtualization and Software Defined Networks (NFV-SDN).

[197] Vaggu N.M., Barpanda R.S. (2024). DBlock-RLB: An energy efficient framework for intelligent routing and trading based load balancing in SDWSN environment. Ad Hoc Netw..

[198] Latah M., Kalkan K. (2022). CWT-DPA: Component-wise waiting time for BC-enabled data plane authentication. Comput. Netw..

[199] Ahmed N. On The Practicality of Blockchain-based Security and Privacy for Next Generation SDN. Proceedings of the 2023 10th International Conference on Wireless Networks and Mobile Communications (WINCOM).

[200] Shashidhara R., Ahuja N., Lajuvanthi M., Akhila S., Das A.K., Rodrigues J.J.P.C. (2021). SDN-chain: Privacy-preserving protocol for software defined networks using blockchain. Secur. Priv..

[201] Sebbar A., Boulmalf M. BCDS-SDN: Privacy and Trusted Data Sharing Using Blockchain Based on a Software-Defined Network’s Edge Computing Architecture. Proceedings of the ICC 2023—IEEE International Conference on Communications.

[202] Gao Y., Chen Y., Hu X., Lin H., Liu Y., Nie L. (2021). Blockchain Based IIoT Data Sharing Framework for SDN-Enabled Pervasive Edge Computing. IEEE Trans. Ind. Inform..

[203] Jiang S., Yang L., Gao X., Zhou Y., Feng T., Song Y., Liu K., Cheng G. (2022). BSD-Guard: A Collaborative Blockchain-Based Approach for Detection and Mitigation of SDN-Targeted DDoS Attacks. Secur. Commun. Netw..

[204] Hajizadeh M., Afraz N., Ruffini M., Bauschert T. Collaborative Cyber Attack Defense in SDN Networks using Blockchain Technology. Proceedings of the 2020 6th IEEE Conference on Network Softwarization (NetSoft).

[205] Derhab A., Guerroumi M., Belaoued M., Cheikhrouhou O. (2021). BMC-SDN: Blockchain-Based Multicontroller Architecture for Secure Software-Defined Networks. Wirel. Commun. Mob. Comput..

[206] Al Sadi A., Mazzocca C., Melis A., Montanari R., Prandini M., Romandini N. (2023). P-IOTA: A Cloud-Based Geographically Distributed Threat Alert System That Leverages P4 and IOTA. Sensors.

[207] Moges E., Han T. DecOp: Decentralized Network Operations in Software Defined Networking using Blockchain. Proceedings of the IEEE INFOCOM 2020—IEEE Conference on Computer Communications Workshops (INFOCOM WKSHPS).

[208] Wang S., Zhu X., Zhao S. Blockchain-Based SDN Security Guarantee Model. Proceedings of the 2019 IEEE 19th International Conference on Communication Technology (ICCT).

[209] Das D., Landrum L.C., Chatterjee P., Ghosh U. (2024). A Scalable Blockchain Framework for Secure Data and Computational Resource Management in SDN. Proceedings of the 2024 IEEE Globecom Workshops (GC Wkshps).

[210] Rathore S., Wook Kwon B., Park J.H. (2019). BlockSecIoTNet: Blockchain-based decentralized security architecture for IoT network. J. Netw. Comput. Appl..

[211] Fernando P., Wei J. Blockchain-Powered Software Defined Network-Enabled Networking Infrastructure for Cloud Management. Proceedings of the 2020 IEEE 17th Annual Consumer Communications Networking Conference (CCNC).

[212] Hoang H.D., Duy P.T., Pham V.H. A Security-Enhanced Monitoring System for Northbound Interface in SDN Using Blockchain. Proceedings of the Tenth International Symposium on Information and Communication Technology.

[213] Duy P.T., Do Hoang H., Thu Hien D.T., Ba Khanh N., Pham V.H. SDNLog-Foren: Ensuring the Integrity and Tamper Resistance of Log Files for SDN Forensics using Blockchain. Proceedings of the 2019 6th NAFOSTED Conference on Information and Computer Science (NICS).

[214] Medury L., Kandah F. (2024). B2-C2: Blockchain-based Flow Control Consistency for Multi-Controller SDN Architecture. Proceedings of the 2024 IEEE International Conference on Consumer Electronics (ICCE).

[215] Sellami B., Hakiri A., Ben Yahia S. (2022). Deep Reinforcement Learning for energy-aware task offloading in join SDN-Blockchain 5G massive IoT edge network. Future Gener. Comput. Syst..

[216] Velmurugadass P., Dhanasekaran S., Shasi Anand S., Vasudevan V. (2021). Enhancing Blockchain security in cloud computing with IoT environment using ECIES and cryptography hash algorithm. Mater. Today Proc..

[217] Almakhour M., Wehby A., Sliman L., Samhat A.E., Mellouk A. Smart Contract Based Solution for Secure Distributed SDN. Proceedings of the 2021 11th IFIP International Conference on New Technologies, Mobility and Security (NTMS).

[218] Song Y., Feng T., Yang C., Mi X., Jiang S., Guizani M. (2023). IS2N: Intent-Driven Security Software-Defined Network with Blockchain. IEEE Netw..

[219] Houda Z.A.E., Hafid A.S., Khoukhi L. (2023). MiTFed: A Privacy Preserving Collaborative Network Attack Mitigation Framework Based on Federated Learning Using SDN and Blockchain. IEEE Trans. Netw. Sci. Eng..

[220] Giri N., Jaisinghani R., Kriplani R., Ramrakhyani T., Bhatia V. Distributed Denial of Service(DDoS) Mitigation in Software Defined Network using Blockchain. Proceedings of the 2019 Third International Conference on I-SMAC (IoT in Social, Mobile, Analytics and Cloud) (I-SMAC).

[221] Zhang X., Huang Z., Huang L., Yang H. (2024). Edge Computing-enabled Network Architecture Design for Space-Air-Ground Integrated Network. Proceedings of the 2024 IEEE 7th Advanced Information Technology, Electronic and Automation Control Conference (IAEAC).

[222] Ohri P., Neogi S.G., Sengupta S., Arockiam D., Muttoo S.K. (2024). Blockchain-Based Smart Contract Architecture for Inter-Domain SDN Controller Communication. Proceedings of the 2024 11th International Conference on Reliability, Infocom Technologies and Optimization (Trends and Future Directions) (ICRITO).

[223] Kayathri T., Kumaresan N., Vijayabhasker R. (2023). SDBGPChain: A decentralized low complexity framework to detect and prevent the BGPattacks using SDN with smart contract based Dendrimer tree blockchain. Comput. Netw..

[224] Wang S., Zhang J., Zhang T. (2023). AI-enabled blockchain and SDN-integrated IoT security architecture for cyber-physical systems. Adv. Control Appl..

[225] Ilyas B., Kumar A., Setitra M.A., Bensalem Z.A., Lei H. (2023). Prevention of DDoS attacks using an optimized deep learning approach in blockchain technology. Trans. Emerg. Telecommun. Technol..

[226] Gaba S., Budhiraja I., Makkar A., Garg D. Machine Learning for Detecting Security Attacks on Blockchain using Software Defined Networking. Proceedings of the 2022 IEEE International Conference on Communications Workshops (ICC Workshops).

[227] Goundar S. (2024). Blockchain-AI Integration for Resilient Real-time Cyber Security. Proceedings of the Global Congress on Emerging Technologies (GCET-2024).

[228] Li W., Tan J., Wang Y., Kutyłowski M., Zhang J., Chen C. (2020). A Framework of Blockchain-Based Collaborative Intrusion Detection in Software Defined Networking. Proceedings of the Network and System Security.

[229] Steichen M., Hommes S., State R. ChainGuard—A firewall for blockchain applications using SDN with OpenFlow. Proceedings of the 2017 Principles, Systems and Applications of IP Telecommunications (IPTComm).

[230] Yazdinejad A., Parizi R.M., Dehghantanha A., Choo K.K.R. (2020). P4-to-blockchain: A secure blockchain-enabled packet parser for software defined networking. Comput. Secur..

[231] Cao H., Hu Y., Wang Q., Wu S., Yang L. (2020). A Blockchain-Based Virtual Network Embedding Algorithm for Secure Software Defined Networking. Proceedings of the IEEE INFOCOM 2020—IEEE Conference on Computer Communications Workshops (INFOCOM WKSHPS).

[232] Fenil E., Mohan Kumar P. (2022). ShChain_3D-ResNet: Sharding Blockchain with 3D-Residual Network (3D-ResNet) Deep Learning Model for Classifying DDoS Attack in Software Defined Network. Symmetry.

[233] Garg S., Goyal S., Bhandari A. (2024). Exploring Blockchain in Collaborative DDoS Mitigation. Proceedings of the 2024 IEEE International Conference on Blockchain and Distributed Systems Security (ICBDS).

[234] Hassan M., Gregory M., Li S. Blockchain enhanced BGP4 Security for an SDN based Federation. Proceedings of the 2022 32nd International Telecommunication Networks and Applications Conference (ITNAC).

[235] Feng H., Yan X., Zhou N., Jiang Z., Liu Y. A Cross-domain Collaborative DDoS Defense Scheme Based on Blockchain-SDN in the IoT. Proceedings of the 2021 ACM International Conference on Intelligent Computing and Its Emerging Applications.

[236] Boukria S., Guerroumi M., Romdhani I. BCFR: Blockchain-based Controller Against False Flow Rule Injection in SDN. Proceedings of the 2019 IEEE Symposium on Computers and Communications (ISCC).

[237] Uludag M.K., Karakus M., Guler E., Uludag S. (2024). Adaptive Mitigation of Blackhole Attacks in Blockchain-Enhanced Software Defined Networks. Proceedings of the 2024 IEEE International Performance, Computing, and Communications Conference (IPCCC).

[238] Li C., Zhang Y., Zhang Y., Zhang P. Trusted Virtual Network Embedding in Blockchain-Based Smart Cyber-Physical Systems. Proceedings of the 2022 IEEE/ACM 15th International Conference on Utility and Cloud Computing (UCC).

[239] Alkhamisi A., Katib I., Buhari S.M. Blockchain-Assisted Hybrid Deep Learning-Based Secure Mechanism for Software Defined Networks. Proceedings of the 2023 IEEE International Conference on Consumer Electronics (ICCE).

